# Clinical Significance and Patterns of Potential Drug–Drug Interactions in Cardiovascular Patients: Focus on Low-Dose Aspirin and Angiotensin-Converting Enzyme Inhibitors

**DOI:** 10.3390/jcm13154289

**Published:** 2024-07-23

**Authors:** Nina D. Anfinogenova, Vadim A. Stepanov, Alexander M. Chernyavsky, Rostislav S. Karpov, Elena V. Efimova, Oksana M. Novikova, Irina A. Trubacheva, Alla Y. Falkovskaya, Aleksandra S. Maksimova, Nadezhda I. Ryumshina, Tatiana A. Shelkovnikova, Wladimir Y. Ussov, Olga E. Vaizova, Sergey V. Popov, Alexei N. Repin

**Affiliations:** 1 Cardiology Research Institute, Tomsk National Research Medical Center, Russian Academy of Sciences, 634012 Tomsk, Russia; 2 Research Institute of Medical Genetics, Tomsk National Research Medical Center, Russian Academy of Sciences, 634050 Tomsk, Russia; 3 Meshalkin National Medical Research Center, 630055 Novosibirsk, Russia; 4 Siberian State Medical University, Ministry of Health of the Russian Federation, 634050 Tomsk, Russia

**Keywords:** cardiovascular disease, polypharmacy, potential drug–drug interaction, aspirin, angiotensin-converting enzyme inhibitor, COVID-19

## Abstract

**Objective**: This study assessed the patterns and clinical significance of potential drug–drug interactions (pDDIs) in patients with diseases of the cardiovascular system. **Methods**: Electronic health records (EHRs), established in 2018–2023, were selected using the probability serial nested sampling method (*n* = 1030). Patients were aged 27 to 95 years (65.0% men). Primary diagnosis of COVID-19 was present in 17 EHRs (1.7%). Medscape Drug Interaction Checker was used to characterize pDDIs. The Mann–Whitney U test and chi-square test were used for statistical analysis. **Results**: Drug numbers per record ranged from 1 to 23 in T-List and from 1 to 20 in P-List. In T-List, 567 drug combinations resulted in 3781 pDDIs. In P-List, 584 drug combinations resulted in 5185 pDDIs. Polypharmacy was detected in 39.0% of records in T-List versus 65.9% in P-List (*p*-value < 0.05). The rates of serious and monitor-closely pDDIs due to ‘aspirin + captopril’ combinations were significantly higher in P-List than in T-List (*p*-value < 0.05). The rates of serious pDDIs due to ‘aspirin + enalapril’ and ‘aspirin + lisinopril’ combinations were significantly lower in P-List compared with the corresponding rates in T-List (*p*-value < 0.05). Serious pDDIs due to administration of aspirin with fosinopril, perindopril, and ramipril were detected less frequently in T-List (*p*-value < 0.05). **Conclusions**: Obtained data may suggest better patient adherence to ‘aspirin + enalapril’ and ‘aspirin + lisinopril’ combinations, which are potentially superior to the combinations of aspirin with fosinopril, perindopril, and ramipril. An abundance of high-order pDDIs in real-world clinical practice warrants the development of a decision support system aimed at reducing pharmacotherapy-associated risks while integrating patient pharmacokinetic, pharmacodynamic, and pharmacogenetic information.

## 1. Introduction

Global prevalence of multiple health conditions exceeds 37% [1] and increases the chances of clinically significant interactions between drugs administered for the treatment of coexisting conditions. Potential drug–drug interactions (pDDIs) may lead to adverse drug reactions (ADRs) [2]. In hospitalized patients with cardiovascular diseases (CVDs), pDDI prevalence reaches 97%, with most pDDIs of major severity and a pharmacodynamic basis [3,4,5]. The weighted prevalence of major to contraindicated pDDIs is nearly 30% in COVID-19 patients administered with ritonavir-containing therapy in the U.S. [6]. Over half of hospitalized cardiovascular patients experience one or more drug therapy problems including unnecessary drug therapy [7].

Drug combinations commonly reported in patients with cardiovascular diseases include antiplatelet drugs and anticoagulants responsible for the majority of serious pDDIs in cardiovascular patients [3,5,8,9]. For example, if combined, aspirin and clopidogrel increase each other’s toxicity by pharmacodynamic synergism, and personalized administration of antiplatelet treatment for cardiovascular disorders seems promising [10]. The common combination of clopidogrel with proton pump inhibitors (PPIs) decreases the effects of clopidogrel by interfering with the metabolism of hepatic enzyme CYP2C19, which is involved in the production of an active metabolite [11]. In the case of combining clopidogrel with PPIs, the safety and efficiency of the therapy may be improved by a pharmacogenomics-guided approach, especially in diabetic and elderly individuals [12]. Aspirin/clopidogrel, furosemide/aspirin, enoxaparin/clopidogrel, and lisinopril/aspirin are among the most frequently occurring interacting drug combinations [13]. A high incidence of pDDIs due to the combination of aspirin and angiotensin-converting enzyme (ACE) is also observed in cardiovascular patients [3]. The coadministration of aspirin with ACE inhibitors may significantly affect renal function and interfere with the antihypertensive effect of ACE inhibitors via the NSAID-mediated downregulation of vasodilating prostaglandin synthesis in the kidneys [14]. The situation worsens in the case of triple therapy [15] and may be even more severe in cases of hyper-polypharmacy, which commonly occur in older cardiovascular patients [3]. The most clinically important pDDIs in COVID-19 patients with CVDs result from the administration of hydroxychloroquine with metformin, beta-blockers, aspirin, and insulin. These pDDIs potentially increase risks of QT prolongation, hypoglycemia, and bleeding [9]. Though it is impossible to completely avoid pDDIs in real clinical practice, the careful choice and regular review of administered medications can reduce potential ADRs [16]. Dynamic monitoring of the international normalized ratio, kidney function, glycemia, blood pressure, heart rate, and acid–base balance where appropriate may significantly reduce potential ADRs in cardiovascular patients.

The Cardiovascular Pharmacotherapy Working Group, established by the European Society of Cardiology, recommends practicing a multidisciplinary team-work approach to address the problems associated with polypharmacy and pDDIs [17]. Implementation of deprescribing protocols in the presence of polypharmacy and serious pDDIs may require reconsidering the guidelines for vulnerable patients [18,19]. Establishing multidisciplinary teams to care for multimorbidity patients may improve patient safety [20,21,22]. Various algorithms analyzing pharmacotherapy administration are available to assess pDDIs [23,24,25,26,27]. The Medscape Interaction Checker allows stratifying the pDDIs into the categories of clinical significance and explains underlying pDDI mechanisms [28,29,30,31,32].

Real-world data (RWD) generating real-world evidence (RWE) represent the internationally recognized trends in the medical research revolutionizing health care [33,34]. RWD provide RWE, which may be used for medical research, drug development [35], risk assessment, pDDI management [36], dose titration in patients with genetic polymorphisms affecting drug metabolism, and regulatory and clinical decision making and as a parallel data source augmenting the findings of randomized clinical trials [33,34,37]. Real-world care for cardiovascular patients often results in the occurrence of numerous pDDIs despite compliance with current official guidelines for the treatment of diseases [19,38,39]. Electronic health records (EHRs) are a valuable RWD/RWE source [34,40].

The patterns of pDDIs due to pharmacotherapy administered to cardiovascular patients during the COVID-19 pandemic remain poorly understood considering that new drug combinations were introduced into clinical practice in recent years [41,42]. The aim of this study was to elucidate the patterns of pDDIs and polypharmacy in patients with CVDs of all ages based on data derived from medical charts recorded in a health information system in Tomsk Region in 2018–2023.

## 2. Materials and Methods

### 2.1. Ethics

This study was conducted in compliance with the standards of Good Clinical Practice and the Declaration of Helsinki. The protocol of this study was approved by the local Biomedical Ethics Committee on 28 June 2022 (record no. 230). This paper is the second planned article presenting data from the study registered at ClinicalTrials.gov (https://clinicaltrials.gov/study/NCT05336565 (accessed on 19 July 2024)) (NCT05336565). All data were depersonalized before the assessment.

### 2.2. Inclusion Criteria

This study analyzed patients with a group of disorders of the heart and blood vessels including coronary heart disease, cerebrovascular disease, hypertensive heart disease, peripheral arterial disease, rheumatic heart disease, congenital heart disease, deep vein thrombosis, and pulmonary embolism. Inclusion criteria were diagnosis of disease of the cardiovascular system verified by a cardiologist and the availability of an electronic health record in the medical information system.

### 2.3. Characteristics of Sample and Study Design

**Figure 1:**
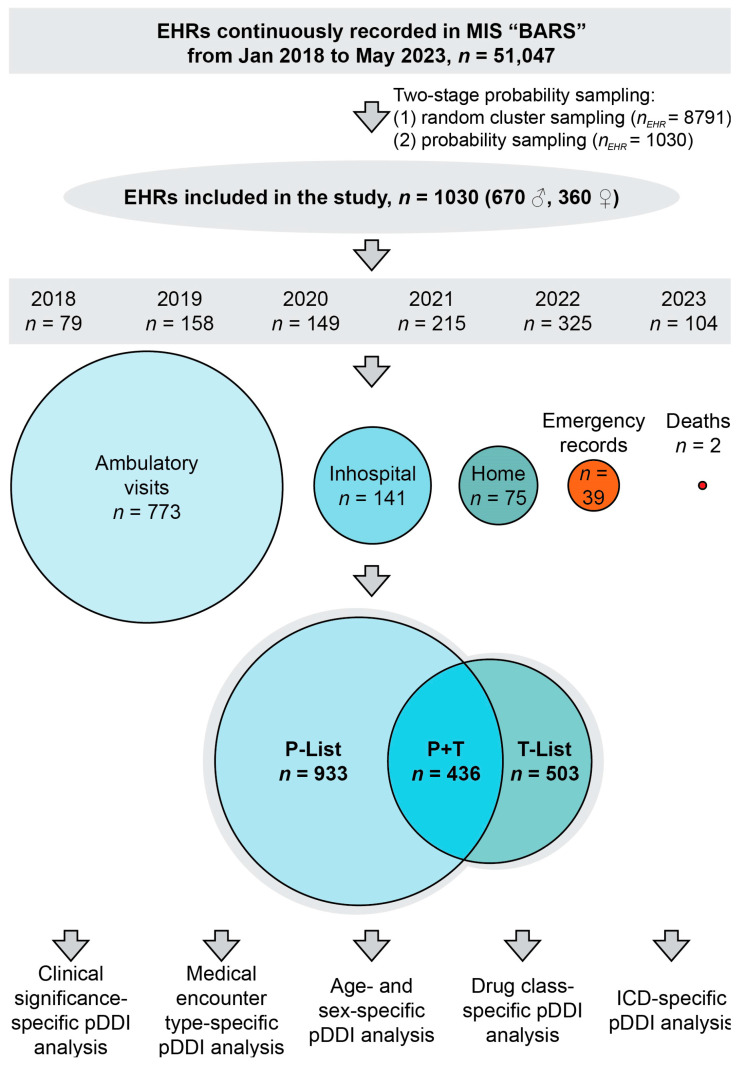
Observational cross-sectional study design. Note: EHRs—electronic health records, MIS—medical information system, pDDI—potential drug–drug interactions, P-List—the list of prescribed medications, T-List—the list of taken medications.

The EHRs were selected using the probability serial nested sampling method. Briefly, a total of 51,047 EHRs belonging to patients with diseases of the cardiovascular system, residing in Tomsk and Tomsk Region, were available in the health information system (JSC “BARS Group”, Novosibirsk, Russia) at the time of investigation. Probability serial nested sampling was performed as follows: first, a random cluster of records, established in 2018–2023, was selected depending on the first letter of the patient’s last name, and the records (*n* = 8791) were coded while deidentifying patients; then, 1030 records were randomly selected to be included in this study. The calculation of the sample size is described in Section 2.6.1. Patient sex was determined according to data present in a patient ID document used for personal identification at the time of establishing the medical record. The chart showing the study design of this observational cross-sectional study is presented in Figure 1. The diagnosis of cardiovascular disease in all patients was verified by a cardiologist in a specialized cardiovascular center. The EHRs were created by various health care specialists affiliated with 24 different healthcare institutions in Tomsk and Tomsk Region and recorded in the regional health information system in 2018–2023. Medical conditions other than diseases of the cardiovascular system in the study sample are presented in Supplementary Table S1. The unstructured text of a total 1030 EHRs was analyzed.

### 2.4. Medication Lists

Most EHRs consisted of unstructured text records specifying the pharmacotherapy administered to patients at the time of the medical care encounter. Two categories of medication lists were established, namely, the list of prescribed medications (P-List) and the list of medications taken by patients (T-List). These lists were analyzed at several levels ranging from the level of an entire study cohort to the level of an individual medical care encounter. The specific lists of taken and prescribed medications were established depending on patient age, sex, primary International Classification of Diseases (ICD) category, and secondary ICD category associated with a given medical care encounter. Individual combinations of medications within a single EHR were also assessed. The overlap of prescribed and taken medications was observed at an individual level, but drug combinations and pDDIs often differed between the T- and P-Lists within a single medical record. Data concerning medications were processed using international nonproprietary names (INNs). Data extraction from the P-/T-Lists showing drug combinations associated with contraindicated (prohibited) and serious (dangerous) pDDIs is given in Table 1.

### 2.5. Polypharmacy, pDDIs, and pDDI Index

Prevalence rates of pharmacotherapy, polypharmacy, and pDDIs were assessed as absolute numbers and percentages where appropriate. Polypharmacy was defined as pharmacotherapy involving the simultaneous use of five or more drugs.

pDDIs were identified on a pairwise basis using Medscape Drug Interaction Checker [43] and stratified into four categories including contraindicated (prohibited), serious (dangerous), requiring close monitoring, and minor drug interactions. Serious pDDIs involved clinically significantly pharmacodynamic antagonism or synergism; increased toxicity of each other; diminished therapeutic effects of each other; significantly decreased renal function; significantly affected metabolism of hepatic enzyme CYP2C19, hepatic/intestinal enzyme CYP3A4, and other enzymes resulting in the altered level or effect of each other; increased anticoagulation; prolonged QTc interval; increased risk of anaphylaxis and Stevens–Johnson syndrome; increased antihypertensive channel blocking; increased risk of bleeding; decreased antiplatelet effects of low-dose aspirin; enhanced risk of hemorrhage; increased risk for myopathy/rhabdomyolysis; increased risks of hypotension, hyperkalemia, and renal impairment; increased risk of infection; decreased renal clearance; and other unspecified interaction mechanisms. All pDDIs are defined in detailed in the Supplementary Materials (Tables S4–S11).

Considering that individual EHRs documented multiple pDDIs belonging to four different categories depending on their clinical significance, the pDDI index was calculated for each EHR to better characterize pDDI impact as previously described [3]. Briefly, four weight coefficients were introduced to characterize the clinical significance of drug interactions, as follows: ‘1’ for minor pDDIs, ‘2’ for monitor-closely pDDIs, ‘3’ for serious/dangerous pDDIs, and ‘4’ for contraindicated pDDIs. The values of the pDDI index were determined as the sum of the appropriate weight coefficients multiplied by the number of matching pDDIs using the following formula:

(1) $$pDDI\;index=\left(4\times n_{c}\right)+\left(3\times n_{s}\right)+\left(2\times n_{m-c}\right)+\left(1\times n_{m}\right),$$

where *n_c_*, *n_s_*, *n_m_*_−*c*_, and *n_m_* were the numbers of contraindicated (*c*), serious (*s*), monitor-closely (*m* − *c*), and minor (*m*) pDDIs, respectively, within a single unstructured text record of prescribed or taken medications in a given EHR.

### 2.6. Statistics

#### 2.6.1. Calculation of Sample Size

The prevalence of serious pDDIs was assumed to range from 17% to 81% [3,6,44,45,46]. The approximate population size, confidence level, and acceptable margin of error were considered 1,000,000, 95%, and 5%, respectively. Similar to a previous study [3], the response distribution was assumed to be 18%, and a sample size of 385 was considered sufficient to evaluate pharmacotherapy characteristics. A pilot study in our cohort showed that only ~37% of EHRs contained sufficient information regarding the presence of pDDIs between prescribed medications while providing detailed data on pharmacotherapy taken by patients. Taking into account these considerations, the sample size was increased to 1030.

#### 2.6.2. Statistical Data Analysis

Statistical data analysis was performed using STATISTICA 10 and Microsoft Excel 2010 software. Figures and illustrations were prepared using STATISTICA 10, Microsoft Excel 2010, and Adobe Illustrator. The Kolmogorov–Smirnov and Shapiro–Wilk tests were used as normality tests to determine whether sample data were drawn from a normal distribution. Data are presented as absolute numbers, percentages, and median with interquartile range when applicable. The Mann–Whitney U test was used to compare two samples of variables with a non-normal distribution. The chi-square test was applied to compare categorical variables using 2 × 2 contingency tables. Values were assumed statistically significant when the *p*-value was < 0.05.

## 3. Results

### 3.1. Sample Characteristics

**Figure 2:**
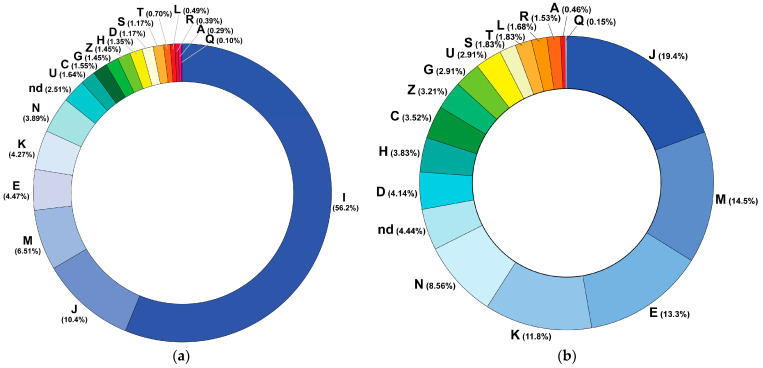
(**a**) The ICD structure of primary diagnoses (*n* = 1030). (**b**) ICD categories of morbidities other than circulatory system diseases (I). Only the first letters of ICD categories are provided. Note: J—diseases of the respiratory system; M—diseases of the musculoskeletal system and connective tissue; E—endocrine, nutritional, and metabolic diseases; K—diseases of the digestive system; N—diseases of the genitourinary system; ‘nd’—not defined; D—diseases of the blood and blood-forming organs and certain disorders involving the immune mechanism; H—diseases of the eye and adnexa and diseases of the ear and mastoid process; C—neoplasms; Z—factors influencing health status and contact with health services; G—diseases of the nervous system; I—diseases of the circulatory system; U—codes for special purposes; S and T—injury, poisoning, and certain other consequences of external causes; L—diseases of the skin and subcutaneous tissue; R—symptoms, signs, and abnormal clinical and laboratory findings not elsewhere classified; A—intestinal infectious diseases; Q—congenital malformations, deformations, and chromosomal abnormalities.

**Figure 3:**
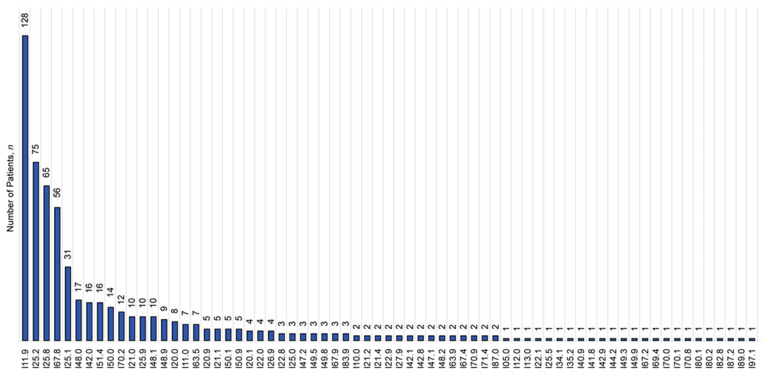
ICD structure defining diseases of the circulatory system (I00–I99) according to data derived from the electronic health records established in 2018–2023 (*n* = 1030). ICD codes are given along the horizontal axis as follows: I11.9—hypertensive heart disease without heart failure; I25.2—old myocardial infarction; I25.8—other forms of chronic ischemic heart disease; I67.8—other specified cerebrovascular diseases; I25.1—atherosclerotic heart disease of native coronary artery; I48.0—paroxysmal atrial fibrillation; I42.0—dilated cardiomyopathy; I51.4—myocarditis, unspecified; I50.0—congestive heart failure; I70.2—atherosclerosis of arteries of extremities; I21.0—acute transmural myocardial infarction of anterior wall; I25.9—chronic ischemic heart disease, unspecified; I48.1—persistent atrial fibrillation; I48.9—atrial fibrillation and atrial flutter; I20.0—unstable angina pectoris; I11.0—hypertensive heart disease with (congestive) heart failure; I63.5—cerebral infarction due to unspecified occlusion or stenosis of cerebral arteries; I20.9—angina pectoris, unspecified; I21.1—ST elevation (STEMI) myocardial infarction of inferior wall; I50.1—left ventricular failure; I50.9—heart failure, unspecified; I20.1—angina pectoris with documented spasm; I22.0—subsequent ST elevation (STEMI) myocardial infarction of anterior wall; I26.9—pulmonary embolism without mention of acute cor pulmonale; I22.8—subsequent myocardial infarction of other sites; I25.0—atherosclerotic cardiovascular disease; I47.2—ventricular tachycardia; I49.5—sick sinus syndrome; I49.8—other specified cardiac arrhythmias; I67.9—cerebrovascular disease, unspecified; I83.9—varicose veins of lower extremities; I10.0—benign essential hypertension; I21.2—ST elevation (STEMI) myocardial infarction of other sites; I21.4—non-ST elevation (NSTEMI) myocardial infarction; I22.9—subsequent myocardial infarction of unspecified site; I27.9—pulmonary heart disease, unspecified; I42.1—obstructive hypertrophic cardiomyopathy; I42.8—other cardiomyopathies; I47.1—supraventricular tachycardia; I48.2—chronic atrial fibrillation; I63.9—cerebral infarction, unspecified; I67.4—hypertensive encephalopathy; I70.9—generalized and unspecified atherosclerosis; I71.4—abdominal aortic aneurysm; I87.0—postthrombotic syndrome; I05.0—rheumatic mitral stenosis; I12.0—hypertensive chronic kidney disease; I13.0—hypertensive heart and chronic kidney disease with heart failure and stage 1 through stage 4 chronic kidney disease, or unspecified chronic kidney disease; I22.1—subsequent myocardial infarction of inferior wall; I25.5—ischemic cardiomyopathy; I34.1—nonrheumatic mitral (valve) prolapse; I35.2—nonrheumatic aortic (valve) stenosis with insufficiency; I40.9—acute myocarditis, unspecified; I41.8—myocarditis in other diseases classified elsewhere; I42.9—cardiomyopathy, unspecified; I44.2—atrioventricular block, complete; I49.3—ventricular premature depolarization; I49.9—cardiac arrhythmia, unspecified; I67.2—cerebral atherosclerosis; I69.4—sequelae of stroke, not specified as hemorrhage or infarction; I70.0—atherosclerosis of aorta; I70.1—atherosclerosis of renal artery; I70.8—atherosclerosis of other arteries; I80.1—phlebitis and thrombophlebitis of femoral vein; I80.2—phlebitis and thrombophlebitis of other deep vessels of lower extremities; I82.8—embolism and thrombosis of other specified veins; I87.2—venous insufficiency (chronic) (peripheral); I89.0—lymphedema, not elsewhere classified; I97.1—other postprocedural cardiac functional disturbances.

Out of a total of 1030 EHRs included in the assessment, 670 (65.0%) records belonged to men and 360 (35.0%) belonged to women. The records were created during ambulatory patient visits (*n* = 773, 75.1%), home visits by primary care physicians (*n* = 77, 7.5%), patient stays in emergency assessment units (*n* = 37, 3.6%), hospital discharge procedures (discharge epicrisis records, *n* = 141, 13.7%), and postmortem documentation (*n* = 2, 0.2%) from January 2018 to May 2023. EHRs contained individual lists of medications prescribed to patients and taken by patients in 933 (90.6%) and 503 (48.7%) records, respectively. The morbidity structure of the study cohort is presented in Figure 2 and Figure 3 and Supplementary Table S1. The baseline profile of patients is presented in Table 2. The median age of female patients significantly exceeded the corresponding value in men (*p*-value = 4.629 × 10^−12^). The number of comorbidities in patients ranged from 0 to 6.

### 3.2. Pharmacotherapy Patterns

Among the EHRs with available data on drug therapy, drug number in a single medical chart ranged from 1 to 23 in the T-List (Me = 5, IQR 3–8; *n* = 503) and from 1 to 20 in the P-List (Me = 6, IQR 4–9; *n* = 933). The median number of prescribed medications per single record significantly exceeded the corresponding median number of taken medications, *p*-value = 0.00134. The median number of medications per record in the P-List significantly exceeded the corresponding value in the T-List in men but not women, *p*-value = 0.004242. When assessed within age groups, the median number of prescribed medications significantly exceeded the corresponding number of taken drugs only in the 80–89-year-old group (*p*-value = 0.04596).

**Figure 4:**
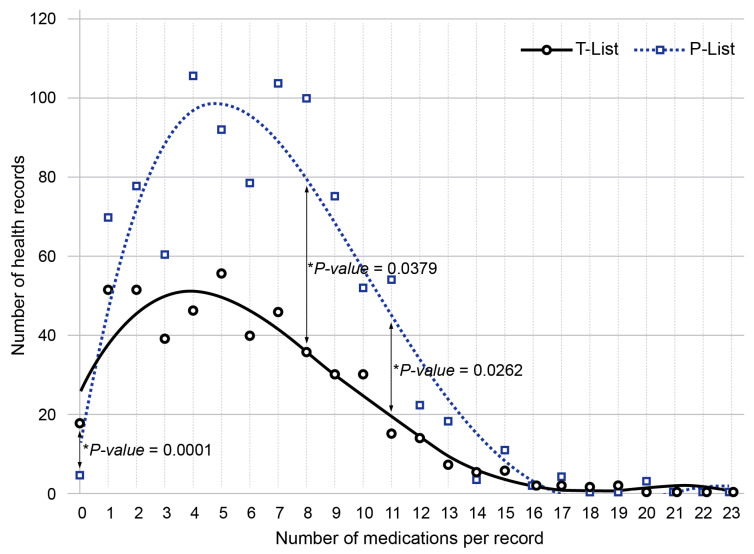
Distribution of patient numbers (vertical axis) depending on the number of medications per record (horizontal axis) in the records of taken (T-List, black circles) and prescribed (P-List, dark blue squares) drugs. The black solid line and dark blue dashed line are the polynomial trends for the corresponding drug lists.

Figure 4 shows a distribution of patients depending on the number of taken and prescribed medications recorded in their EHRs. The number of records with zero medications in the P-List was significantly lower than in the T-List (*p*-value = 0.0001), while the groups of patients prescribed with 8 or 11 medications were relatively larger than the corresponding groups of patients taking the same numbers of medications (*p*-value = 0.0379 and *p*-value = 0.0262, respectively).

Supplementary Table S2 provides the median numbers of drugs, pDDIs, and pDDI index values per record in the lists of taken drugs and prescribed medications in cardiovascular patients depending on the primary diagnosis category established during the medical encounter. Supplementary Table S3 provides the median numbers of serious, monitor-closely, and minor pDDIs per record in the records of taken and prescribed medications in patients depending on the primary diagnosis category established during the medical encounter.

### 3.3. Polypharmacy

Polypharmacy was detected significantly less often in the T-List (39.0% of records) than in the P-List (65.9% of records), *p*-value = 0.0107. However, in patients with polypharmacy, the median numbers of taken and prescribed drugs per record did not significantly differ: Me = 8, IQR 6–10 versus Me = 8, IQR 6–10 (*p*-value = 0.5189).

### 3.4. pDDIs and pDDI Index Values

The prevalence rates of pDDIs were 67.33% and 66.17% in the T- and P-Lists, respectively. The absolute number of pDDIs per single medical record varied from 0 to 54 in the T-List and from 0 to 39 in the P-List. The median pDDI numbers per single record were Me = 3, IQR 0–9 in the T-List versus Me = 3, IQR 0–8 in the P-List (*p*-value = 0.1059). The detailed mechanisms of pairwise pDDIs are described in Supplementary Tables S4–S11.

The differences in pDDI numbers between the P-List and T-List were insignificant in any clinical significance category of pDDIs in our cohort.

In the list of taken medications, we identified a total of 567 pairwise drug combinations associated with pDDIs, and the number of pDDI occurrences (*n_pDDI_*) reached 3781. These drug pairs included 454 drug combinations resulting in pDDIs requiring close monitoring (*n_pDDI_* = 3379); 106 medication pairs resulted in minor pDDIs (*n_pDDI_* = 355); 60 pairwise drug combinations were associated with serious pDDIs (*n_pDDI_* = 242); and 3 combinations were associated with contraindicated pDDIs (*n_pDDI_* = 4).

In the P-List, we identified a total of 584 drug combinations associated with pDDIs, and these drug combinations were associated with a nearly nine-fold number of pDDIs (*n_pDDI_* = 5185). These drug combinations involved 409 pairs resulting in pDDIs requiring close monitoring (*n_pDDI_* = 4213); 102 combinations resulted in minor pDDIs (*n_pDDI_* = 474); 69 drug pairs were associated with serious (dangerous) pDDIs (*n_pDDI_* = 486); and 4 combinations were contraindicated (*n_pDDI_* = 13).

The drug combinations associated with contraindicated pDDIs were indapamide + sotalol, amitriptyline + indapamide, and carbamazepine + linezolid in the T-List and captopril + sacubitril/valsartan, amitriptyline + indapamide, apixaban + carbamazepine, and lisinopril + sacubitril/valsartan in the P-List.

**Figure 5:**
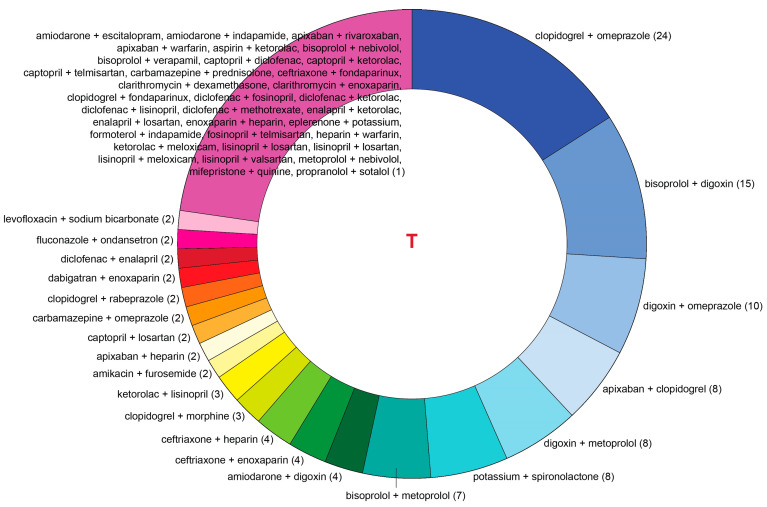
Pairs of taken medications (T-List) resulting in serious (dangerous) pDDIs except for the combinations of aspirin and angiotensin-converting enzyme (ACE) inhibitors, which are not shown in the figure but discussed below in more detail. Numbers in parentheses correspond to pDDI occurrences for each pairwise drug combination.

**Figure 6:**
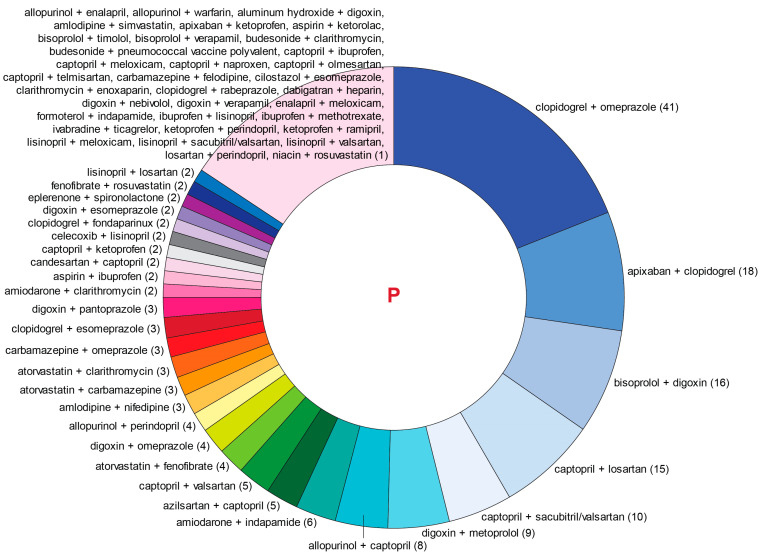
Pairs of prescribed medications (P-List) resulting in serious (dangerous) pDDIs except for the combinations of aspirin and angiotensin-converting enzyme (ACE) inhibitors, which are not shown in this figure but discussed below in more detail. Numbers in parentheses represent pDDI occurrences for each pairwise drug combination.

Figure 5 and Figure 6 show a distribution of drug combinations associated with serious (dangerous) pDDIs in the T-List and P-List, respectively, except for the most abundant combinations of aspirin and ACE inhibitors involving low-dose aspirin, which are characterized in the designated subsection below.

The most abundant combinations of low-dose aspirin and ACE inhibitors associated with serious pDDIs are characterized in the section to follow (Section 3.6). A comparison of the other top-five combinations resulting in serious pDDIs between the T-List and P-List showed significant differences only for the rates of the “digoxin + omeprazole” combination, which occurred five times more often in the T-List compared with the P-List (*p*-value = 0.0055).

The values of the pDDI indexes ranged from 0 to 105 in the T-List and from 0 to 77 in the P-List, respectively. The median values of the pDDI indexes did not significantly differ between the T-List and P-List in the study cohort. The top-five absolute values of the pDDI indexes were observed in I, A, J, N, and E ICD categories in the P-List and I, A, J, E, and K ICD categories in the T-List.

### 3.5. In-Hospital versus Ambulatory Patterns of Polypharmacy and pDDIs

**Figure 7:**
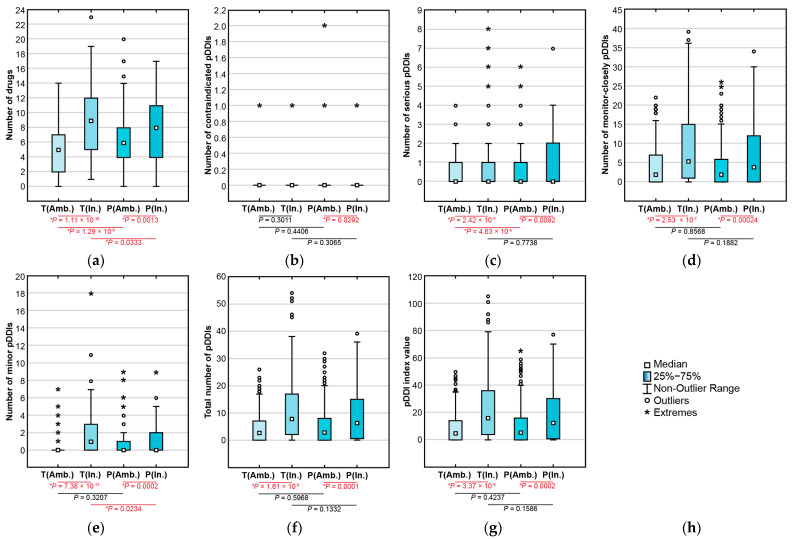
Median numbers of drugs (**a**), contraindicated pDDIs (**b**), serious pDDIs (**c**), monitor-closely pDDIs (**d**), minor pDDIs (**e**), total pDDIs (**f**), and pDDI index values (**g**) in the lists of taken and prescribed medications based on ambulatory and in-hospital electronic health records in cardiovascular patients. Subfigure (**h**) is the key explaining symbols used in the images (**a**–**g**). Note: T(Amb.)—the T-List derived from ambulatory records, T(In.)—the T-List derived from in-hospital records (discharge epicrises), P(Amb.)—the P-List derived from ambulatory records, P(In.)—the P-List derived from in-hospital records (discharge epicrises).

**Figure 8:**
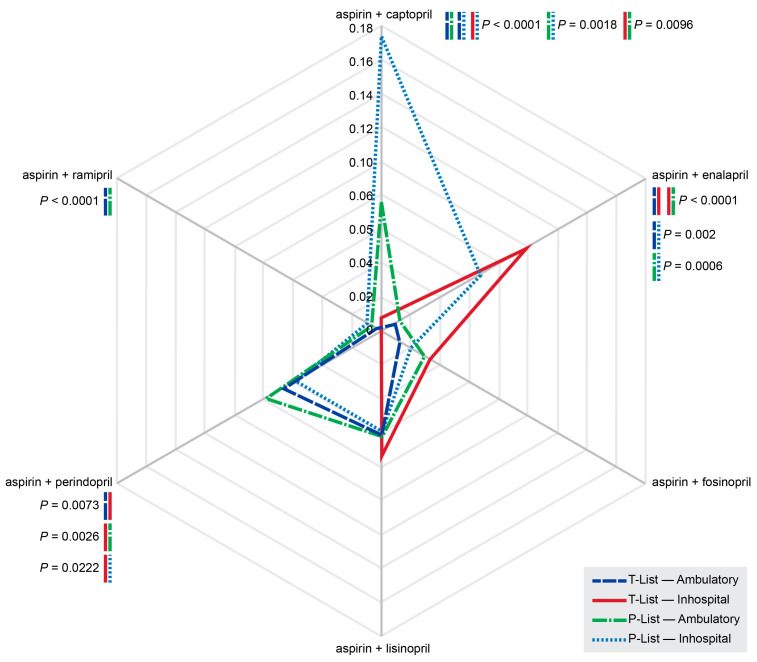
Probabilities of six different combinations of aspirin with ACE inhibitors in the T- and P-Lists in patients with diseases of the cardiovascular system based on ambulatory records (outpatient) and hospital discharge epicrises (inpatient). Note: T-List—the list of taken medications; P-List—the list of prescribed medications.

Significant differences were observed in the median numbers of medications, pDDI numbers, and pDDI index values between the T- and P-Lists derived from outpatient and inpatient EHRs (Figure 7). Significant differences were observed in the patterns of administered combinations of aspirin + ACE inhibitors between the in-hospital and ambulatory records (Figure 8).

### 3.6. ‘Aspirin + ACE Inhibitor’ Combinations

Six ACE inhibitors were assessed including captopril, enalapril, fosinopril, lisinopril, perindopril, and ramipril. In cases of serious pDDIs, we observed 92 ‘aspirin + ACE inhibitor’ combinations in the T-List (out of a total 242 combinations associated with serious pDDIs) and 270 combinations in the P-List (out of 486). In cases of monitor-closely pDDIs, we found 158 combinations in the T-List (out of 3379) and 283 combinations in the P-List (out of 4213).

Table 3 and Table 4 show the rates of ‘aspirin + ACE inhibitor’ combination occurrences associated with serious (dangerous) pDDIs and pDDIs requiring close monitoring in the T-List versus the P-List. The rates of ‘aspirin + captopril’ combinations associated with both serious and monitor-closely pDDIs significantly exceeded the corresponding values in the P-List compared with the T-List (*p*-value < 0.0001 and = 0.0409 for the serious and monitor-closely pDDIs, respectively). On the contrary, the rates of ‘aspirin + enalapril’ and ‘aspirin + lisinopril’ combinations, associated with the serious pDDIs, were significantly lower in the P-List than in the T-List (*p*-value = 0.0020 and = 0.0014, respectively). The total occurrence of all six ‘aspirin + ACE inhibitor’ combinations, associated with serious and monitor-closely pDDIs, in the P-List significantly exceeded the corresponding occurrences in the T-List (*p*-value < 0.0001) (Table 3 and Table 4).

The omission of the ‘aspirin + captopril’ combination from the analysis did not change the overall pattern, and the prevalence of ‘aspirin + ACE inhibitor’ still significantly exceeded the corresponding rate in the P-List relative to the T-List (*p*-value = 0.0314 for serious pDDIs and *p*-value < 0.0001 for monitor-closely pDDIs).

The prevalence of aspirin administration associated with serious pDDIs among other drugs associated with serious pDDIs in the P-List (28.1%) significantly exceeded the corresponding rate in the T-List (19.2%) (*p*-value = 0.0003). A similar pattern was observed in the prevalence of aspirin administration associated with monitor-closely pDDIs: 20.7% in the T-List versus 13.6% in the T-List (*p*-value < 0.0001).

The probabilities of using different combinations of aspirin with ACE inhibitors significantly differed between the outpatient ambulatory records and inpatient discharge epicrises (Figure 8).

## 4. Discussion

The primary goal of our study was to identify the overall burden of polypharmacy and pDDIs in cardiovascular patients in various types of medical encounters at the premises of different healthcare institutions and during home visits. This study assessed the patterns and clinical significance of pDDIs in cardiovascular patients based on the unstructured text of electronic medical records created during the COVID-19 pandemic. This study demonstrated that despite compliance with current official guidelines for the treatment of diseases [17,38,39], real-world clinic care for cardiovascular patients may often result in the occurrence of numerous serious pDDIs.

This study showed a significantly higher prevalence of pDDIs in the P-List, but median values of pDDIs and the pDDI index did not significantly differ between the P-List and T-List in the entire cohort of adult cardiovascular patients, unlike in earlier findings in older patients with cardiovascular diseases [3]. The rates of polypharmacy and prevalence of serious pDDIs in our cohort agree with the reports of other research teams [19,48,49]. The rates of polypharmacy and pDDIs were significantly higher in the medication lists derived from the hospital discharge epicrises than from the ambulatory records.

Interestingly, the structure of ‘aspirin + ACE inhibitor’ combinations in our study significantly differed between the T-List and P-List. Significant differences were also observed in the probabilities of various ‘aspirin + ACE inhibitor’ combinations depending on medical encounter type, i.e., ambulatory medical charts versus hospital discharge epicrises. According to manufacturers’ instructions, the clinical impact of pDDIs of ‘aspirin + captopril’ and ‘aspirin + enalapril’ may be insignificant if a daily aspirin dose does not exceed 300 mg. However, the abundance of higher-order pDDIs, the controversial clinical significance of low-dose aspirin administration [31,50,51,52,53,54,55,56,57], and the administration of a wider array of ACE inhibitors, not limited to captopril or enalapril only, provided the rationale for the detailed characterization of ‘aspirin + ACE inhibitor’ combinations.

We assessed the administration of six ACE inhibitors (captopril, enalapril, fosinopril, lisinopril, perindopril, and ramipril) combined with aspirin. These combinations resulted in hundreds of potentially serious and monitor-closely drug interactions. The rates of serious and monitor-closely pDDIs due to the ‘aspirin + captopril’ combination were significantly higher in the prescribed medication records compared with those in the T-List. On the contrary, the rates of ‘aspirin + enalapril’ and ‘aspirin + lisinopril’ combinations, associated with serious pDDIs, were significantly lower in the P-List relative to the T-List. These data may suggest better safety and tolerability (and thus better patient adherence) of ‘aspirin + enalapril’ and ‘aspirin + lisinopril’ combinations, which in terms of adherence may be potentially superior to the combinations of aspirin with fosinopril, perindopril, and ramipril detected less frequently in the T-List in the study cohort. The differences in pDDIs between the T- and P-Lists are complex and could, at least partially, originate from patients’ nonadherence, which remains a global underappreciated and underaddressed health problem [58,59]. Significant asymmetry in the probability diagram for the administration of six different ‘aspirin + ACE inhibitor’ combinations (Figure 8) may suggest the presence of additional, previously unrecognized factors contributing to the overall pDDI burden in cardiovascular patients. These factors could potentially originate from poorly understood policies of prescribing in different clinical settings, e.g., inpatient versus outpatient care, as well as from undisclosed marketing processes. A better understanding of these factors is required to ensure pharmacotherapy safety.

Drug interaction checkers [23,24,25,26,27,60,61] usually stratify the clinical significance of pDDIs irrespective of genetic variations, which could markedly increase or ameliorate the severity of pDDIs. Current guidelines on pDDIs and polypharmacy do not provide straightforward recommendations on estimating the clinical effect of genetic polymorphisms affecting pDDIs. There is a lack of understanding the complex interactions caused by pDDIs and drug–gene interactions (DGIs) in the presence of various biotransformation pathways known as drug–gene–gene interactions (DGGIs) [62]. In cases of genetic variations in drug metabolism, the clinical significance of pDDIs may increase, potentially causing the transition of the pDDI category to a more serious level, for example, from monitor-closely to serious or from serious to the contraindicated category. The clinical relevance of such a situation increases in the presence of comorbidities and polypharmacy.

Genetic variations of patients may, at least partially, explain the observed differences between the T- and P-Lists. Most patients in our study were hypertensive, and hypertension is a polygenic disease [63]. Responses to angiotensin-converting enzyme inhibitors depend on pharmacogenetic variants [64], and there is a clinically significant interaction between genetic factors and response to aspirin. In particular, there is an interaction between a genetic variant in the guanylate cyclase soluble subunit alpha-3 (*GUCY1A3*) gene and the outcome of primary CVD prevention with aspirin [53]. Aspirin significantly reduces (21%) cardiovascular risk in the two-thirds of individuals homozygous for the *rs7692387* risk (*G*) allele. The other third of patients who are heterozygous (*G/A*) show a significant increase (by 39%) in the rates of major CVD when randomized to aspirin. Therefore, aspirin administration significantly contributes to primary prevention in homozygotes of CVD risk allele (*G*) but is associated with ~1.4-fold risk increase in carriers of the nonrisk allele (*A*) [53]. The coronary artery disease risk gene is associated with ischemic events after coronary intervention, suggesting an interaction between genotype stratum and aspirin intake [65]. A meta-analysis of data from the ISAR-ASPI registry, the PLATO trial, and the UCORBIO study biobank comprising thousands of patients showed that homozygous *GUCY1A3* risk allele carriers have a 1.7-fold greater risk for cardiovascular death or stent thrombosis after coronary intervention [54]. These data do not include data on bleeding outcomes.

There are numerous other genes significantly related to aspirin resistance [66,67]. One of the most dangerous complications of ACE inhibitor therapy is angioedema, and pDDIs may trigger this life-threatening condition [68]. Triple therapy involving an ACE inhibitor, a dipeptidyl peptidase-IV inhibitor, and a calcium channel blocker is reported to be associated with angioedema [69]. Pharmacogenetic markers of angioedema as a secondary side effect to enalapril is described in hypertensive patients [70]. Significant associations are found between enalapril-triggered dry cough and genetic polymorphisms [71]. Cytochrome P450 family 2 subfamily C member 9 gene (*CYP2C9*) polymorphisms affect the antihypertensive and hypouricemic effects of losartan in hypertensive patients [72].

The exceedance of the ‘digoxin + omeprazole’ combination in the T-List may be explained by higher rates of self-administration in patients taking polypharmacy, but this hypothesis requires further research.

Future efforts are needed to elucidate the clinically relevant effects of genetic variations on drug-metabolizing enzymes and to determine the epigenetic pathways involved in the responsiveness to antihypertensive and other cardiovascular medications [63,64,73,74,75]. A genetically guided approach to antihypertensive therapy using a multigene panel would allow healthcare professionals to avoid major adverse events and decrease medical expenses. According to estimations, such an approach may reduce total three-year costs by almost half, and almost 90% of these savings originate from preventing specific ADRs [74,76]. The pharmacogenomic approach to risk prevention in patients taking ACE inhibitors warrants further exploration. Real-world drug outcome data, augmented by genetic technologies and resources, may contribute to the discovery of previously unknown, clinically relevant drug–drug–gene interactions to establish highly sought medical decision support systems able to avert ADRs and improve treatment outcomes in the presence of polypharmacy [62,77]. Taking into account genetic variations may significantly improve pharmacotherapy safety in multimorbid patients.

Irrespective of pharmacogenomics, improving the safety of pharmacotherapy in patients with polypharmacy is vital. The assessment of pharmacotherapy patterns in a resuscitated sudden cardiac arrest population using forensic toxicology techniques allowed the researchers to identify the prescribed and nonprescribed drugs where polypharmacy (≥5 drugs) was found in 19% of individuals aged 18–90 years, and drugs associated with QT prolongation were found in 12% of patients. Though forensic approaches are of limited value, close attention should be paid to polypharmacy in the context of sudden cardiac arrest [78]. In our study, several drug combinations linked to an increased risk of QTc interval prolongation were observed including amiodarone + clarithromycin, amiodarone + escitalopram, amiodarone + formoterol, amiodarone + indapamide, amitriptyline + indapamide, fluconazole + levofloxacin, fluconazole + ondansetron, fluconazole + trimethoprim, formoterol + indapamide, indapamide + sotalol, levofloxacin + moxifloxacin, and mifepristone + quinine. The coadministration of QT-prolonging drugs should be avoided whenever possible.

A limitation of our study is the pairwise pDDI assessment due to the unavailability of resources for the evaluation of higher-order pDDIs, though research is ongoing to solve this issue [79].

## 5. Conclusions

The abundance and complexity of high-order pDDIs observed in cardiovascular patients with polypharmacy in real-world clinical practice warrants the development and implementation of a decision support system aimed at minimizing pharmacotherapy-associated risks while integrating patient pharmacokinetic, pharmacodynamic, and pharmacogenetic information. Knowledge of the patient’s genetic profile and of multidisciplinary team building may contribute to better patient compliance and adherence to treatment by reducing potential ADRs and enhancing pharmacotherapy efficacy.

## Figures and Tables

**Table 1:** Data extraction from the P-/T-Lists showing occurrences of contraindicated and serious pDDIs in alphabetical order.

Pairwise Drug Combination	T-List, *n*	P-List, *n*	Pairwise Drug Combination	T-List, *n*	P-List, *n*
**Contraindicated pDDIs**					
amitriptyline + indapamide	1	1	carbamazepine + linezolid	1	0
apixaban + carbamazepine	0	1	indapamide + sotalol	2	0
captopril + sacubitril/valsartan	0	10	lisinopril + sacubitril/valsartan	0	1
**Serious pDDIs**					
allopurinol + captopril	0	8	ceftriaxone + heparin	4	0
allopurinol + enalapril	0	1	celecoxib + lisinopril	0	2
allopurinol + perindopril	0	3	cilostazol + esomeprazole	0	1
allopurinol + warfarin	0	1	clarithromycin + dexamethasone	1	0
aluminum hydroxide + digoxin	0	1	clarithromycin + enoxaparin	1	1
amikacin + furosemide	2	0	clopidogrel + esomeprazole	0	3
amiodarone + clarithromycin	0	2	clopidogrel + fondaparinux	1	2
amiodarone + digoxin	4	0	clopidogrel + morphine	3	0
amiodarone + escitalopram	1	0	clopidogrel + omeprazole	24	41
amiodarone + indapamide	1	6	clopidogrel + rabeprazole	2	1
amlodipine + nifedipine	0	3	dabigatran + enoxaparin	2	0
amlodipine + simvastatin	0	1	dabigatran + heparin	0	1
apixaban + clopidogrel	8	18	diclofenac + enalapril	2	0
apixaban + heparin	2	0	diclofenac + fosinopril	1	0
apixaban + ketoprofen	0	1	diclofenac + ketorolac	1	0
apixaban + rivaroxaban	1	0	diclofenac + lisinopril	1	0
apixaban + warfarin	1	0	diclofenac + methotrexate	1	0
aspirin + captopril	3	84	digoxin + esomeprazole	0	2
aspirin + enalapril	18	20	digoxin + metoprolol	8	9
aspirin + fosinopril	10	28	digoxin + nebivolol	0	1
aspirin + ibuprofen	0	2	digoxin + omeprazole	10	4
aspirin + ketorolac	1	1	digoxin + pantoprazole	0	3
aspirin + lisinopril	36	58	digoxin + verapamil	0	1
aspirin + perindopril	25	74	enalapril + ketorolac	1	0
aspirin + ramipril	0	6	enalapril + losartan	1	0
atorvastatin + carbamazepine	0	3	enalapril + meloxicam	0	1
atorvastatin + clarithromycin	0	3	enoxaparin + heparin	1	0
atorvastatin + fenofibrate	0	4	eplerenone + potassium	1	0
azilsartan + captopril	0	5	eplerenone + spironolactone	0	2
bisoprolol + digoxin	15	16	fenofibrate + rosuvastatin	0	2
bisoprolol + metoprolol	7	0	fluconazole + ondansetron	2	0
bisoprolol + nebivolol	1	0	formoterol + indapamide	1	1
bisoprolol + timolol	0	1	fosinopril + telmisartan	1	0
bisoprolol + verapamil	1	1	heparin + warfarin	1	0
budesonide + clarithromycin	0	1	ibuprofen + lisinopril	0	1
budesonide + pneumococcal vaccine polyvalent	0	1	ibuprofen + methotrexate	0	1
candesartan + captopril	0	2	ivabradine + ticagrelor	0	1
captopril + diclofenac	1	0	ketoprofen + perindopril	0	1
captopril + ibuprofen	0	1	ketoprofen + ramipril	0	1
captopril + ketoprofen	0	2	ketorolac + lisinopril	3	0
captopril + ketorolac	1	0	ketorolac + meloxicam	1	0
captopril + losartan	2	15	levofloxacin + sodium bicarbonate	2	0
captopril + meloxicam	0	1	lisinopril + losartan	2	0
captopril + naproxen	0	1	lisinopril + losartan	0	2
captopril + olmesartan	0	1	lisinopril + meloxicam	1	1
captopril + sacubitril/valsartan	0	10	lisinopril + sacubitril/valsartan	0	1
captopril + telmisartan	1	1	lisinopril + valsartan	1	1
captopril + valsartan	0	5	losartan + perindopril	0	1
carbamazepine + felodipine	0	1	metoprolol + nebivolol	1	0
carbamazepine + omeprazole	2	3	mifepristone + quinine	1	0
carbamazepine + prednisolone	1	0	niacin + rosuvastatin	0	1
ceftriaxone + enoxaparin	4	0	potassium + spironolactone	8	0
ceftriaxone + fondaparinux	1	0	propranolol + sotalol	1	0

**Table 2:** Baseline characteristics of sample based on information derived from electronic health records.

Characteristic	Value
White/Caucasian, *n* (%)	1030 (100)
Men, *n* (%)	670 (65.0)
Women, *n* (%)	360 (35.0)
Age (males), median (IQR), years	63 (56; 71)
Age (females), median (IQR), years	71 (61; 77)
Outpatient visits, *n* (%)	773 (75.1)
Home consultations, *n* (%)	77 (7.5)
In-hospital care, *n* (%)	141 (13.7)
Emergency events, *n* (%)	37 (3)
Postmortem documentation, *n* (%)	2 (0.2)
Primary diagnosis of COVID-19, *n* (%)	17 (1.7)
Estimated history of COVID-19, *n* (%)	145 (14.1)
Time of creating health records	January 2018–May 2023

**Table 3:** ‘Aspirin + ACE inhibitor’ combination occurrences associated with serious (dangerous) pDDIs.

Aspirin + ACE Inhibitor * Combination Associated with Serious pDDIs	T-List, *n* (%)	P-List, *n* (%)	P-to-T-List Percentage Ratio	*p*-Value
Aspirin + captopril	3 (1.24)	84 (17.3)	13.94	<0.0001
Aspirin + enalapril	18 (7.44)	20 (4.12)	0.553	0.0020
Aspirin + fosinopril	10 (4.13)	28 (5.76)	1.394	1
Aspirin + lisinopril	36 (14.9)	58 (11.9)	0.802	0.0014
Aspirin + perindopril	25 (10.3)	74 (15.2)	1.474	0.9203
Aspirin + ramipril	0 (0.00)	6 (1.24)	n.a. **	0.3323
Aspirin + ACE inhibitor	92 (38.0)	270 (55.6)	1.461	<0.0001

* In the majority of cases, captopril was administered when blood pressure remained elevated despite taking other antihypertensive medications, in accordance with the recommendations of the Russian Medical Society on Arterial Hypertension (RMSAH) [47] for the treatment of uncomplicated hypertensive crisis. Digits in parentheses represent the percentage of potentially serious ‘aspirin + ACE inhibitor’ occurrences relative to the total number of serious pDDIs (T-List: *n* = 242; P-List: *n* = 486). ** n.a. stands for “not applicable” as dividing by zero is not allowed.

**Table 4:** ‘Aspirin + ACE inhibitor’ combination occurrences associated with pDDIs requiring close monitoring.

Aspirin + ACE Inhibitor Combination Associated with Monitor-Closely pDDIs	T-List, *n* (%)	P-List, *n* (%)	P-to-T-List Percentage Ratio	*p*-Value
Aspirin + captopril	32 (0.95)	84 (1.99)	2.105	0.0409
Aspirin + enalapril	22 (0.65)	22 (0.52)	0.802	0.0574
Aspirin + fosinopril	14 (0.41)	30 (0.71)	1.719	0.6714
Aspirin + lisinopril	48 (1.42)	63 (1.50)	1.053	0.0769
Aspirin + perindopril	40 (1.18)	78 (1.85)	1.564	0.6892
Aspirin + ramipril	2 (0.06)	6 (0.14)	2.406	0.7913
Aspirin + ACE inhibitor	158 (4.68)	283 (6.72)	1.437	<0.0001

Digits in parentheses represent the percentage of ‘aspirin + ACE inhibitor’ occurrences requiring close monitoring relative to the total number of monitor-closely pDDIs (T-List: *n* = 3379; P-List: *n* = 4213).

## Data Availability

The data that support the findings of this study are available from the corresponding author upon reasonable request.

## Supplementary Materials

The following supporting information can be downloaded at: https://www.mdpi.com/article/10.3390/jcm13154289/s1, Table S1. Medical conditions other than diseases of the cardiovascular system coded with ICD categories in study sample (*n* = 1001); Table S2. Median numbers of drugs and pDDIs per record and median values of pDDI index in the T-List and the P-List in patients depending on primary diagnosis category; Table S3. Median numbers of serious, monitor-closely, and minor pDDIs in the T-List and the P-List in patients depending on primary diagnosis category; Table S4: Drug combinations resulting in contraindicated potential drug–drug interactions in the list of taken medications in patients with cardiovascular diseases according to data derived from electronic health records (*n* = 1030) established in 2018–2023; Table S5: Drug combinations resulting in serious potential drug–drug interactions in the list of taken medications in patients with cardiovascular diseases according to data derived from electronic health records (*n* = 1030) established in 2018–2023; Table S6: Drug combinations resulting in potential monitor-closely drug–drug interactions in the list of taken medications in patients with cardiovascular diseases according to data derived from electronic health records (*n* = 1030) established in 2018–2023; Table S7: Drug combinations resulting in potential minor drug–drug interactions in the list of taken medications in patients with cardiovascular diseases according to data derived from electronic health records (*n* = 1030) established in 2018–2023; Table S8: Drug combinations resulting in potential contraindicated drug–drug interactions in the list of prescribed medications in patients with cardiovascular diseases according to data derived from electronic health records (*n* = 1030) established in 2018–2023; Table S9: Drug combinations resulting in potential serious drug–drug interactions in the list of prescribed medications in patients with cardiovascular diseases according to data derived from electronic health records (*n* = 1030) established in 2018–2023; Table S10: Drug combinations resulting in potential monitor-closely drug–drug interactions in the list of prescribed medications in patients with cardiovascular diseases according to data derived from electronic health records (*n* = 1030) established in 2018–2023; Table S11: Drug combinations resulting in potential minor drug–drug interactions in the list of prescribed medications in patients with cardiovascular diseases according to data derived from electronic health records (*n* = 1030) established in 2018–2023.
