# Supplementary material for: Clinical Significance and Patterns of Potential Drug–Drug Interactions in Cardiovascular Patients: Focus on Low-Dose Aspirin and Angiotensin-Converting Enzyme Inhibitors
Source: J Clin Med. 2024 Jul 23;13(15):4289. doi: 10.3390/jcm13154289 (PMC11313610; doi:10.3390/jcm13154289)
Supplement: Supplementary file 1 [file jcm-13-04289-s001.zip › jcm-3082610-supplementary.pdf]

Supplementary Material

Clinical Significance and Patterns of Potential Drug-Drug Interactions in Cardiovascular Patients: Observational Cross-Sectional Analytical Study

Nina D. Anfinogenova [1,*], Vadim A. Stepanov [2], Alexander M. Chernyavsky [3], Rostislav S. Karpov [1], Elena V. Efimova [1], Oksana M. Novikova [1], Irina A. Trubacheva [1], Alla Y. Falkovskaya [1], Aleksandra S. Maksimova [1], Nadezhda I. Ryumshina [1], Tatiana A. Shelkovnikova [1], Wladimir Y. Ussov [1,3], Olga A. Vaizova [4], Sergey V. Popov [1], and Alexei N. Repin [1]

1 Cardiology Research Institute, Tomsk National Research Medical Center, Russian Academy of Sciences, Tomsk, Russia; cardio.intl@gmail.com
2 Research Institute of Medical Genetics, Tomsk National Research Medical Center, Russian Academy of Sciences, Tomsk, Russia
3 Meshalkin National Medical Research Center, Novosibirsk, Russia
4 Siberian State Medical University, Ministry of Health of the Russian Federation, Tomsk, Russia
* Correspondence: Cardio.intl@gmail.com; Tel.: +73822554111 ext. 5321

Table S1. Medical conditions other than diseases of the cardiovascular system coded with ICD categories in study sample (*n* = 1030).

ICD category	Condition	Percentage, %
J06.9	Acute upper respiratory infection	3.6
E11.7	Type 2 diabetes mellitus with multiple complications	2.7
E11.9	Type 2 diabetes mellitus without complications	1.7
N40	Benign prostatic hyperplasia without lower urinary tract symptoms	1.6
U07.1	COVID-19	1.5
J06.8	Other acute upper respiratory infections of multiple sites	1.3
J12.8	Other viral pneumonia	1.3
J18.9	Pneumonia, unspecified organism	1.3
K29.9	Gastroduodenitis	1.1
M42.1	Adult osteochondrosis of spine	1.1
K81.1	Chronic cholecystitis	1
J44.8	Other specified chronic obstructive pulmonary disease	0.9
E04.2	Nontoxic multinodular goiter	0.8
E11.8	Type 2 diabetes mellitus with unspecified complications	0.8
K80.1	Calculus of gallbladder with other cholecystitis	0.8
M54.4	Lumbago with sciatica	0.8
N18.3	Chronic kidney disease, stage 3	0.8
J44.9	Chronic obstructive pulmonary disease, unspecified	0.7
D64.9	Anemia, Unspecified	0.6
E66.0	Obesity due to excess calories	0.6
J20.9	Acute Bronchitis, Unspecified	0.6

M19.9	Osteoarthritis, unspecified site	0.6
M51.1	Lumbar and other intervertebral disc disorders with radiculopathy	0.6
Z76.0	Encounter for issue of repeat prescription	0.6
C83.0	Small cell B-cell lymphoma	0.5
D50.9	Iron deficiency anemia, unspecified	0.5
E78.8	Other disorders of lipoprotein metabolism	0.5
N11.8	Other chronic tubulo-interstitial nephritis	0.5
E11.6	Type 2 diabetes mellitus With other specified complications	0.4
G62.8	Other specified polyneuropathies	0.4
G93.8	Other specified disorders of brain	0.4
H25.1	Age-related nuclear cataract	0.4
J02.9	Acute pharyngitis, unspecified	0.4
J42	Unspecified chronic bronchitis	0.4
J45.9	Other and unspecified asthma	0.4
K25.9	Gastric ulcer Unspecified as acute or chronic, without haemorrhage or perforation	0.4
K26.7	Chronic duodenal ulcer without hemorrhage or perforation	0.4
K29.6	Other gastritis	0.4
K76.0	Fatty (change of) liver, not elsewhere classified	0.4
K86.1	Other chronic pancreatitis	0.4
N28.1	Cyst of kidney, acquired	0.4
R73.9	Hyperglycemia, unspecified	0.4
T75.2	Effects of vibration	0.4
Z00.8	Encounter for other general examination	0.4
B18.2	Chronic viral hepatitis C	0.3
E06.3	Autoimmune thyroiditis	0.3
H35.3	Degeneration of macula and posterior pole	0.3
H90.3	Sensorineural hearing loss, bilateral	0.3
J41.0	Simple chronic bronchitis	0.3
J45.8	Mixed asthma	0.3
K21.0	Gastro-esophageal reflux disease with esophagitis, without bleeding	0.3
K29.5	Chronic gastritis	0.3
K52.9	Noninfective gastroenteritis and colitis	0.3
K91.5	Postcholecystectomy syndrome	0.3
L90.8	Other atrophic disorders of skin	0.3
M05.8	Other rheumatoid arthritis with rheumatoid factor	0.3
M15.9	Polyosteoarthritis, unspecified	0.3
M17.0	Bilateral primary osteoarthritis of knee	0.3
M50.3	Other cervical disc degeneration	0.3
M53.1	Cervicobrachial syndrome	0.3
M54.6	Pain in thoracic spine	0.3

M54.8	Other dorsalgia	0.3
M65.8	Other synovitis and tenosynovitis	0.3
N20.0	Calculus of kidney	0.3
C44.3	Other malignant neoplasms: Skin of other and unspecified parts of face	0.2
C54.1	Malignant neoplasm: Endometrium	0.2
C67.8	Malignant neoplasm of overlapping sites of bladder	0.2
D44.1	Neoplasm of uncertain or unknown behaviour: Adrenal gland	0.2
D64.8	Other specified anemias	0.2
E01.8	Other iodine-deficiency-related thyroid disorders and allied conditions	0.2
E06.5	Other chronic thyroiditis	0.2
E10.7	Type 1 diabetes mellitus With multiple complications	0.2
E11.2	Type 2 diabetes mellitus with kidney complications	0.2
E11.5	ype 2 diabetes mellitus with circulatory complications	0.2
H25.8	Other age-related cataract	0.2
J12.9	Viral pneumonia, unspecified	0.2
J16.8	Pneumonia due to other specified infectious organisms	0.2
J90	Pleural effusion, not elsewhere classified	0.2
K21.9	Gastro-oesophageal reflux disease	0.2
K29.4	Chronic atrophic gastritis	0.2
K29.7	Gastritis	0.2
K58.9	Irritable bowel syndrome without diarrhea	0.2
K59.0	Constipation	0.2
K59.9	Functional intestinal disorder, unspecified	0.2
K64.1	Second degree hemorrhoids	0.2
K64.8	Other hemorrhoids	0.2
K71.2	Toxic liver disease with acute hepatitis	0.2
K81.0	Acute cholecystitis	0.2
K85.9	Acute pancreatitis	0.2
M10.0	Idiopathic gout	0.2
M15.8	Other polyosteoarthritis	0.2
M16.5	Unilateral post-traumatic osteoarthritis of hip	0.2
M17.1	Unilateral primary osteoarthritis of knee	0.2
M17.2	Bilateral post-traumatic osteoarthritis of knee	0.2
M17.9	Osteoarthritis of knee, unspecified	0.2
M53.8	Other specified dorsopathies.	0.2
M54.2	Cervicalgia	0.2
M54.5	Low back pain	0.2
M54.9	Dorsalgia, unspecified	0.2
N18.2	Chronic kidney disease, stage 2 (mild)	0.2
N18.4	Chronic kidney disease, stage 4 (severe)	0.2

N41.1	Chronic prostatitis	0.2
R73.0	Abnormal glucose	0.2
S00.0	Unspecified superficial injury of scalp	0.2
S52.50	Fracture of lower end of radius: Unspecified	0.2
T91.1	Sequelae of fracture of spine	0.2
Z00.0	Encounter for general adult medical examination without abnormal findings	0.2
Z25.1	Need for immunization against influenza	0.2
A02.0	Salmonella enteritis	0.1
A41.1	Sepsis due to other specified staphylococcus	0.1
A46	Erysipelas	0.1
B25.9	Cytomegaloviral disease, unspecified	0.1
B66.0	Opisthorchiasis	0.1
B99	Other and unspecified infectious diseases	0.1
C18.0	Malignant neoplasm of cecum	0.1
C18.7	Malignant neoplasm: Sigmoid colon	0.1
C18.8	Malignant neoplasm: Overlapping lesion of colon	0.1
C20	Malignant neoplasm of rectum	0.1
C34.1	Malignant neoplasm: Upper lobe, bronchus or lung	0.1
C50.3	Malignant neoplasm: Lower-inner quadrant of breast	0.1
C50.4	Malignant neoplasm: Upper-outer quadrant of breast	0.1
C50.9	Malignant neoplasm: Breast, unspecified	0.1
C61	Malignant neoplasm of prostate	0.1
C67.4	Malignant neoplasm: Posterior wall of bladder	0.1
C78.0	Secondary malignant neoplasm of lung	0.1
C81.1	Nodular sclerosis (classical) Hodgkin lymphoma	0.1
D12.0	Benign neoplasm of cecum	0.1
D14.3	Benign neoplasm: Bronchus and lung	0.1
D23.5	Other benign neoplasm of skin of trunk	0.1
D30.0	Benign neoplasm of kidney	0.1
D33.4	Benign neoplasm of spinal cord	0.1
D37.7	Neoplasm of uncertain or unknown behaviour: Other digestive organs	0.1
D38.1	Neoplasm of uncertain behavior of trachea, bronchus and lung	0.1
D45	Polycythemia vera	0.1
D47.9	Neoplasm of uncertain or unknown behaviour of lymphoid, haematopoietic and related tissue, unspecified	0.1
D68.8	Other specified coagulation defects	0.1
D69.6	Thrombocytopenia, unspecified	0.1
D75.9	Disease of blood and blood-forming organs, unspecified	0.1
E03.8	Other specified hypothyroidism	0.1
E04.1	Nontoxic single thyroid nodule	0.1

E05.9	Thyrotoxicosis	0.1
E10.6	Type 1 diabetes mellitus With other specified complications	0.1
E11.3	Type 2 diabetes mellitus with ophthalmic complications	0.1
E11.4	Type 2 diabetes mellitus With neurological complications	0.1
E11.7	Type 2 diabetes mellitus With multiple complications	0.1
E16.8	Other specified disorders of pancreatic internal secretion	0.1
E21.1	Secondary hyperparathyroidism, not elsewhere classified	0.1
E66.8	Other obesity	0.1
E68	Sequelae of hyperalimentation	0.1
E78.0	Pure hypercholesterolemia	0.1
E78.5	Hyperlipidemia, Unspecified	0.1
E79.0	Hyperuricemia without signs of inflammatory arthritis and tophaceous disease	0.1
E87.5	Hyperkalemia	0.1
F10.3	Mental and behavioural disorders due to use of alcohol Withdrawal state	0.1
G00.9	Bacterial meningitis, unspecified	0.1
G20	Parkinson's disease	0.1
G40.2	Localization-related (focal)(partial) symptomatic epilepsy and epileptic syndromes with complex partial seizures	0.1
G40.3	Generalized idiopathic epilepsy and epileptic syndromes	0.1
G45.0	Vertebro-basilar artery syndrome	0.1
G50.0	Trigeminal neuralgia	0.1
G52.8	Disorders of other specified cranial nerves	0.1
G57.1	Meralgia paresthetica	0.1
G63.2	Diabetic polyneuropathy	0.1
G70.0	Myasthenia gravis	0.1
G91.8	Other hydrocephalus	0.1
G93.4	Other and unspecified encephalopathy	0.1
H00.0	Hordeolum and other deep inflammation of eyelid	0.1
H04.1	Other disorders of lacrimal gland	0.1
H10.4	Chronic conjunctivitis	0.1
H10.8	Other conjunctivitis	0.1
H26.2	Complicated cataract	0.1
H26.9	Unspecified cataract	0.1
H40.0	Glaucoma suspect	0.1
H40.8	Other glaucoma	0.1
H52.0	Hypermetropia	0.1
H52.4	Presbyopia	0.1
H60.5	Acute noninfective otitis externa	0.1
H68.0	Eustachian salpingitis	0.1
H81.1	Benign paroxysmal vertigo	0.1

H90.0	Conductive hearing loss	0.1
H90.8	Mixed conductive and sensorineural hearing loss, unspecified.	0.1
J00	Acute nasopharyngitis	0.1
J01.0	Acute maxillary sinusitis	0.1
J02.8	Acute pharyngitis due to other specified organisms	0.1
J32.9	Chronic sinusitis	0.1
J37.0	Chronic laryngitis	0.1
J94.8	Other specified pleural conditions	0.1
K10.2	Inflammatory conditions of jaws	0.1
K31.7	Polyp of stomach and duodenum	0.1
K31.9	Disease of stomach and duodenum	0.1
K35.8	Other and unspecified acute appendicitis	0.1
K40.9	Unilateral or unspecified inguinal hernia, without obstruction or gangrene	0.1
K51.9	Ulcerative colitis	0.1
K56.5	Intestinal adhesions [bands] with obstruction (postinfection)	0.1
K64.3	Fourth degree hemorrhoids	0.1
K65.9	Peritonitis, unspecified	0.1
K66.0	Peritoneal adhesions (postprocedural) (postinfection)	0.1
K75.9	Inflammatory liver disease, unspecified	0.1
K80.2	Calculus of gallbladder without cholecystitis	0.1
K80.8	Other cholelithiasis	0.1
K82.8	Other specified diseases of gallbladder	0.1
K86.0	Alcohol-induced chronic pancreatitis	0.1
L02.4	Cutaneous abscess, furuncle and carbuncle of limb	0.1
L20.9	Atopic dermatitis, unspecified	0.1
L30.1	Dyshidrosis	0.1
L30.9	Dermatitis, unspecified	0.1
L40.0	Psoriasis vulgaris	0.1
L40.8	Other psoriasis	0.1
L98.4	Chronic ulcer of skin, not elsewhere classified	0.1
L98.9	Disorder of the skin and subcutaneous tissue, unspecified	0.1
M10.9	Gout, Unspecified	0.1
M15.0	Primary generalized (osteo)arthritis	0.1
M16.0	Bilateral primary osteoarthritis of hip	0.1
M16.2	Bilateral osteoarthritis resulting from hip dysplasia	0.1
M16.3	Unilateral osteoarthritis resulting from hip dysplasia	0.1
M17.5	Other unilateral secondary osteoarthritis of knee	0.1
M19.0	Primary osteoarthritis of other joints	0.1
M19.8	Other specified arthrosis	0.1
M23.2	Derangement of meniscus due to old tear or injury	0.1

M24.5	Contracture of joint	0.1
M35.3	Polymyalgia rheumatica	0.1
M42.9	Spinal osteochondrosis	0.1
M45.0	Ankylosing spondylitis of multiple sites in spine	0.1
M50.1	Cervical disc disorder with radiculopathy	0.1
M51.0	Thoracic, thoracolumbar and lumbosacral intervertebral disc disorders with myelopathy	0.1
M53.0	Cervicocranial syndrome	0.1
M65.3	Trigger finger	0.1
M65.9	Synovitis and tenosynovitis, unspecified	0.1
M67.1	Other contracture of tendon (sheath)	0.1
M70.2	Olecranon bursitis	0.1
M75.1	Rotator cuff tear or rupture, not specified as traumatic	0.1
M75.8	Other shoulder lesions	0.1
M79.2	Neuralgia and neuritis	0.1
M81.8	Other osteoporosis without current pathological fracture	0.1
M87.0	Idiopathic aseptic necrosis of bone	0.1
N11.0	Nonobstructive reflux-associated chronic pyelonephriti	0.1
N11.9	Chronic tubulo-interstitial nephritis	0.1
N18.5	Chronic kidney disease, stage 5	0.1
N18.9	Chronic kidney disease	0.1
N20.1	Calculus of ureter	0.1
N20.2	Calculus of kidney with calculus of ureter	0.1
N20.9	Urinary calculus, unspecified	0.1
N23	Unspecified renal colic	0.1
N30.1	Interstitial cystitis	0.1
N30.2	Other chronic cystitis	0.1
N30.9	Cystitis, unspecified	0.1
N43.0	Encysted hydrocele	0.1
N45.9	Orchitis, epididymitis and epididymo-orchitis without abscess	0.1
N60.1	Diffuse cystic mastopathy	0.1
N60.8	Other benign mammary dysplasias	0.1
N63	Unspecified lump in breast	0.1
N85.0	Endometrial hyperplasia	0.1
N95.0	Postmenopausal bleeding	0.1
N95.2	Postmenopausal atrophic vaginitis	0.1
Q24.9	Congenital malformation of heart, unspecified	0.1
R09.1	Pleurisy	0.1
R51	Headache	0.1
R59.9	Enlarged lymph nodes, unspecified	0.1

R71	Abnormality of red blood cells	0.1
R73.9	Hyperglycemia, unspecified	0.1
S05.0	Injury of conjunctiva and corneal abrasion without mention of foreign body	0.1
S20.2	Contusion of thorax	0.1
S40.0	Contusion of shoulder and upper arm	0.1
S50.1	Contusion of forearm	0.1
S51.0	Open wound of elbow	0.1
S70.1	Contusion of thigh	0.1
S80.0	Contusion of knee	0.1
S93.4	Sprain of ankle	0.1
T15.1	Foreign body in conjunctival sac	0.1
T26.6	Chemical burn of cornea and conjunctival sac	0.1
T29.3	Burns of multiple regions, at least one burn of third degree mentioned	0.1
T31.1	Burns involving 10-19% of body surface	0.1
T78.3	Angioneurotic edema	0.1
T90.5	Sequelae of intracranial injury	0.1
T91.3	Sequelae of injury of spinal cord	0.1
U08.9	Personal history of COVID-19, unspecified	0.1
U09.9	Post–COVID-19 condition, unspecified	0.1
W00.4	Fall on same level involving ice and snow	0.1
W22.4	Striking against wall of swimming pool	0.1
Z01.4	Encounter for gynecological examination	0.1
Z03.1	Observation for suspected malignant neoplasm	0.1
Z03.8	Encounter for observation for other suspected diseases and conditions ruled out	0.1
Z54.8	Convalescence following other treatment	0.1
Z71.2	Person consulting for explanation of examination or test findings	0.1
Z96.1	Presence of intraocular lens	0.1

Table S2. Median numbers of drugs, pDDIs, and pDDI index values per record in the T-List and the P-List in cardiovascular patients depending on primary diagnosis category established during the medical encounter.

Clinical entity	Number of EHRs, *n*		Drug number, median (IQR)		*P*-value	pDDI number, median (IQR)		*P*-value	pDDI index, median (IQR)		*P*-value
	T-List	P-List	T-List	P-List		T-List	P-List		T-List	P-List	
A	2	3	15 (1-19)	6 (3.5-11)	.4000	27 (13.5-40.5)	0 (0-18)	.7469	51 (25-76)	0 (0-35)	.7469
C	6	11	2 (1.25-4.25)	4 (2-4)	.5723	0 (0-0)	0 (0-0.5)	.7354	0 (0-0)	0 (0-1)	.7354
D	5	9	4 (3-4)	4 (1-4)	1.000	2 (1-3)	1 (0-1)	.4475	4 (2-6)	2 (0-3)	.4939
E	35	38	3 (1-7)	3 (1-7)	.9469	1 (0-6)	0 (0-2.75)	.2485	2 (0-12)	0 (0-8.25)	.4630
G	6	16	6.5 (4.5-7)	5 (2-5)	.1640	2 (0.5-7.25)	0 (0-1)	.0898	4.5 (1-14)	0 (0-1)	.0553
H	1	14	7 (-7-7)	3 (2.25-4)	-	7 (7-7)	0 (0-0.75)	-	14 (14-14)s	0 (0-1.75)	-
I	292	536	6 (3-8)	7 (5-9)	**1.928e-7**	5 (1-10.25)	6 (2-11)	.0747	11 (1-20)	13 (4-21)	**.0291**
J	45	97	5 (2-9)	6 (4-9)	.0853	2 (0-9)	0 (0-3)	.0688	3 (0-18)	0 (0-6)	.0807
K	15	41	5 (1.5-6)	4 (2-6)	.7727	0 (0-4)	0 (0-1)	.1909	0 (0-7.5)	0 (0-2)	.1911
L	2	5	4.5 (2.75-6.25)	2 (2-3)	.8407	3 (1.5-4.5)	0 (0-0)	.2059	6.5 (3.25-9.75)	0 (0-0)	.2059
M	33	60	3 (1-5)	5 (2-7)	**.0291**	0 (0-3)	0 (0-1.25)	.8808	0 (0-6)	0 (0-2.25)	.9299
N	18	36	4 (2.25-7)	3.5 (1.75-5)	.2395	1.5 (0-7.5)	0 (0-0)	**.0048**	2.5 (0-15.25)	0 (0-0)	**.0041**
Q	1	1	8 (8-8)	9 (9-9)	-	3 (3-3)	0 (0-0)	-	6 (6-6)	12 (12-12)	-
R	1	4	9 (9-9)	7.5 (3.25-11)	-	12 (12-12)	6 (6-6)	-	24 (24-24)	7.5 (0.75-15)	-
S and T	1	4	14 (14-14)	1.5 (1-2)	-	23 (23-23)	7.5 (0.75-15)	-	43 (43-43)	0 (0-0.5)	-
U07.1	12	11	12.5 (8.75-16)	7 (5-13)	.1643	12 (4.5-15.25)	1 (0-2)	**.0010**	21.5 (8.75-27.5)	1 (0-4)	**.0153**
U (other than U07.1)	1	1	0 (0-0)	9 (9-9)	-	0 (0-0)	5 (5-5)	-	0 (0-0)	11 (1-11)	-
Z	3	14	2 (1.5-4.5)	4 (2-8)	.4426	0 (0-3)	1.5 (0-5)	.5564	0 (0-6.5)	2.5 (0-10.5)	.5566

Note: A—Intestinal infectious diseases; C—neoplasms; D—diseases of the blood and blood-forming organs and certain disorders involving the immune mechanism; E—endocrine, nutritional and metabolic diseases; G—diseases of the nervous system; H—diseases of the eye and adnexa and diseases of the ear and mastoid process; I—diseases of the circulatory system; J—diseases of the respiratory system; K—diseases of the digestive system; L—diseases of the skin and subcutaneous tissue; M—diseases of the musculoskeletal system and connective tissue; N—diseases of the genitourinary system; R—symptoms, signs and abnormal clinical and laboratory findings, not elsewhere classified; S and T—injury, poisoning and certain other consequences of external causes; U—codes for special purposes; Z—factors influencing health status and contact with health services.

Table S3. Median numbers of serious, monitor-closely, and minor pDDIs per record in the T-List and the P-List in patients depending on primary diagnosis category established during the medical encounter.

Clinical entity	Number of EHRs, *n*		Serious pDDIs, median (IQR)		*P*-value	Monitor-closely pDDIs, median (IQR)		*P*-value	Minor pDDIs, median (IQR)		*P*-value
	T-List	P-List	T-List	P-List		T-List	P-List		T-List	P-List	
A	2	3	2 (1-3)	0 (0-1)	.7469	20 (9.75-29.25)	0 (0-15)	.7469	5.5 (2.75-8.25)	0 (0-2)	.7469
C	6	11	0 (0-0)	0 (0-0)	.7878	0 (0-0)	0 (0-0.5)	.7354	0 (0-0)	0 (0-0)	.2184
D	5	9	0 (0-0)	0 (0-1)	.6709	2 (1-3)	0 (0-1)	.2844	0 (0-0)	0 (0-0)	.2330
E	35	38	0 (0-0)	0 (0-0)	.2403	1 (0-4)	0 (0-3)	.3920	0 (0-0)	0 (0-0)	.8679
G	6	16	0 (0-1)	0 (0-0)	.1247	1.5 (0.25-4.25)	0 (0-0.5)	.0742	0 (0-2.25)	0 (0-0)	.1401
H	1	14	0 (0-0)	0 (0-0)	-	7 (7-7)	0 (0-0.75)	-	0 (0-0)	0 (0-0)	-
I	292	536	0 (0-1)	1 (0-1)	**.0001**	4 (0-8)	5 (2-8)	.1033	0 (0-1)	0 (0-1)	.9340
J	45	97	0 (0-1)	0 (0-0)	.0703	1 (0-5)	0 (0-2)	.0635	0 (0-1)	0 (0-0)	.1072
K	15	41	0 (0-1)	0 (0-0)	**.0261**	0 (0-3.5)	0 (0-1)	.1839	0 (0-0)	0 (0-0)	.1679
L	2	5	0 (0-0)	0 (0-0)	-	2 (1-3)	0 (0-0)	.2059	0.5 (0.25-0.75)	0 (0-0)	.2059
M	33	60	0 (0-0)	0 (0-0)	.7537	0 (0-3)	0 (0-1)	.4857	0 (0-0)	0 (0-1)	.0778
N	18	36	0 (0-0)	0 (0-0)	**.0224**	1 (0-5.75)	0 (0-0)	**.0046**	0 (0-0)	0 (0-0)	.4619
Q	1	1	0 (0-0)	0 (0-0)	-	3 (3-3)	6 (6-6)	-	0 (0-0)	0 (0-0)	-
R	1	4	0 (0-0)	1 (0-2.25)	-	12 (12-12)	6 (0-12.25)	-	0 (0-0)	0.5 (0-1.25)	-
S and T	1	4	1 (1-1)	0 (0-0)	-	18 (18-18)	0 (0-0.25)	-	4 (4-4)	0 (0-0)	-
U07.1	12	11	0 (0-0)	0 (0-0)	.6685	9 (3.5-11.5)	0 (0-2)	**.0087**	2 (0.75-3.5)	0 (0-0.5)	**.0463**
U (other than U07.1)	1	1	0 (0-0)	1 (1-1)	-	0 (0-0)	4 (4-4)	-	0 (0-0)	0 (0-0)	-
Z	3	14	0 (0-1)	0 (0-0)	.7987	0 (0-2.5)	1 (0-3.75)	.5559	0 (0-0)	0 (0-0.75)	.3513

Note: A—Intestinal infectious diseases; C—neoplasms; D—diseases of the blood and blood-forming organs and certain disorders involving the immune mechanism; E—endocrine, nutritional and metabolic diseases; G—diseases of the nervous system; H—diseases of the eye and adnexa and diseases of the ear and mastoid process; I—diseases of the circulatory system; J—diseases of the respiratory system; K—diseases of the digestive system; L—diseases of the skin and subcutaneous tissue; M—diseases of the musculoskeletal system and connective tissue; N—diseases of the genitourinary system; R—symptoms, signs and abnormal clinical and laboratory findings, not elsewhere classified; S and T—injury, poisoning and certain other consequences of external causes; U—codes for special purposes; Z—factors influencing health status and contact with health services.

Table S4. Drug combinations resulting in contraindicated potential drug–drug interactions in the list of taken drugs in patients with cardiovascular diseases according to data derived from the electronic health records (*n* = 1030) established in 2018–2023.

**Drug combinations**	***n***	**Potential contraindicated drug-drug interactions (T-list)**
indapamide + sotalol	2	indapamide and sotalol both increase QTc interval. Contraindicated
amitriptyline + indapamide	1	amitriptyline and indapamide both increase QTc interval. Contraindicated
carbamazepine + linezolid	1	carbamazepine increases toxicity of linezolid by unknown mechanism. Contraindicated. D/C MAO inhibitor 2 weeks before

Table S5. Drug combinations resulting in serious potential drug–drug interactions in the list of taken medications in patients with cardiovascular diseases according to data derived from the electronic health records (n = 1030) established in 2018–2023.

Drug combinations	n	Potential serious drug-drug interactions (T-list)
aspirin + lisinopril	36	aspirin, lisinopril. pharmacodynamic antagonism. Avoid or Use Alternate Drug. Coadministration may result in a significant decrease in renal function. NSAIDs may diminish the antihypertensive effect of ACE inhibitors. The mechanism of these interactions is likely related to the ability of NSAIDs to reduce the synthesis of vasodilating renal prostaglandins
aspirin + perindopril	25	aspirin, perindopril. pharmacodynamic antagonism. Avoid or Use Alternate Drug. Coadministration may result in a significant decrease in renal function. NSAIDs may diminish the antihypertensive effect of ACE inhibitors. The mechanism of these interactions is likely related to the ability of NSAIDs to reduce the synthesis of vasodilating renal prostaglandins
clopidogrel + omeprazole	24	omeprazole decreases effects of clopidogrel by affecting hepatic enzyme CYP2C19 metabolism. Avoid or Use Alternate Drug. Clopidogrel efficacy may be reduced by drugs that inhibit CYP2C19. Inhibition of platelet aggregation by clopidogrel is entirely due to an active metabolite. Clopidogrel is metabolized to this active metabolite in part by CYP2C19
aspirin + enalapril *	18	aspirin, enalapril. pharmacodynamic antagonism. Avoid or Use Alternate Drug. Coadministration may result in a significant decrease in renal function. NSAIDs may diminish the antihypertensive effect of ACE inhibitors. The mechanism of these interactions is likely related to the ability of NSAIDs to reduce the synthesis of vasodilating renal prostaglandins
bisoprolol + digoxin	15	bisoprolol increases effects of digoxin by pharmacodynamic synergism. Use Caution/Monitor. Enhanced bradycardia
aspirin + fosinopril	10	aspirin, fosinopril. pharmacodynamic antagonism. Avoid or Use Alternate Drug. Coadministration may result in a significant decrease in renal function. NSAIDs may diminish the antihypertensive effect of ACE inhibitors. The mechanism of these interactions is likely related to the ability of NSAIDs to reduce the synthesis of vasodilating renal prostaglandins
digoxin + omeprazole	10	omeprazole will increase the level or effect of digoxin by increasing gastric pH. Applies only to oral form of both agents. Avoid or Use Alternate Drug
apixaban + clopidogrel	8	clopidogrel and apixaban both increase anticoagulation. Avoid or Use Alternate Drug
digoxin + metoprolol	8	digoxin increases toxicity of metoprolol by unspecified interaction mechanism. Avoid or Use Alternate Drug. Can increase risk of bradycardia
potassium + spironolactone	8	spironolactone and potassium [chloride] both increase serum potassium. Avoid or Use Alternate Drug
bisoprolol + metoprolol	7	bisoprolol and metoprolol both increase anti-hypertensive channel blocking. Avoid or Use Alternate Drug
amiodarone + digoxin	4	amiodarone will increase the level or effect of digoxin by P-glycoprotein (MDR1) efflux transporter. Avoid or Use Alternate Drug. Amiodarone increases PO digoxin serum concentrations by ~70% and IV digoxin by ~17%; measure digoxin levels before initiating amiodarone and reduce PO digoxin dose by 30-50%; decrease IV digoxin dose by 15-30%
		amiodarone will increase the level or effect of digoxin by basic (cationic) drug competition for renal tubular clearance. Avoid or Use Alternate Drug. Amiodarone increases PO digoxin serum

		concentrations by ~70% and IV digoxin by ~17%; measure digoxin levels before initiating amiodarone and reduce PO digoxin dose by 30-50%; decrease IV digoxin dose by 15-30%
ceftriaxone + enoxaparin	4	ceftriaxone increases effects of enoxaparin by anticoagulation. Avoid or Use Alternate Drug. cephalosporins may decrease prothrombin activity
ceftriaxone + heparin	4	ceftriaxone will increase the level or effect of heparin by anticoagulation. Avoid or Use Alternate Drug. cephalosporins may decrease prothrombin activity
aspirin + captopril *	3	aspirin, captopril. pharmacodynamic antagonism. Avoid or Use Alternate Drug. Coadministration may result in a significant decrease in renal function. NSAIDs may diminish the antihypertensive effect of ACE inhibitors. The mechanism of these interactions is likely related to the ability of NSAIDs to reduce the synthesis of vasodilating renal prostaglandins
clopidogrel + morphine	3	morphine will decrease the level or effect of clopidogrel by Other (see comment). Avoid or Use Alternate Drug. coadministration of opioid agonists delay and reduce absorption of clopidogrel, presumably because of slowed gastric emptying, resulting in reduced exposure to its metabolites; consider use of parenteral antiplatelet agents in acute coronary syndrome patients requiring coadministration of morphine or other opioid agonists
ketorolac + lisinopril	3	ketorolac, lisinopril. pharmacodynamic antagonism. Avoid or Use Alternate Drug. Coadministration may result in a significant decrease in renal function. NSAIDs may diminish the antihypertensive effect of ACE inhibitors. The mechanism of these interactions is likely related to the ability of NSAIDs to reduce the synthesis of vasodilating renal prostaglandins
amikacin + furosemide	2	furosemide, amikacin. Either increases toxicity of the other by Mechanism: pharmacodynamic synergism. Avoid or Use Alternate Drug. Increased risk of ototoxicity and nephrotoxicity
apixaban + heparin	2	heparin and apixaban both increase anticoagulation. Avoid or Use Alternate Drug
captopril + losartan	2	losartan, captopril. Either increases toxicity of the other by pharmacodynamic synergism. Avoid or Use Alternate Drug. Dual blockade of renin-angiotensin system increases risks of hypotension, hyperkalemia, and renal impairment
carbamazepine + omeprazole	2	carbamazepine will decrease the level or effect of omeprazole by affecting hepatic enzyme CYP2C19 metabolism. Avoid or Use Alternate Drug
clopidogrel + rabeprazole	2	rabeprazole decreases effects of clopidogrel by affecting hepatic enzyme CYP2C19 metabolism. Avoid or Use Alternate Drug. Clopidogrel efficacy may be reduced by drugs that inhibit CYP2C19. Inhibition of platelet aggregation by clopidogrel is entirely due to an active metabolite. Clopidogrel is metabolized to this active metabolite in part by CYP2C19
dabigatran + enoxaparin	2	dabigatran and enoxaparin both increase anticoagulation. Avoid or Use Alternate Drug. Caution is advised, both drugs have the potential to cause bleeding. Concomitant use may increase risk of bleeding
diclofenac + enalapril	2	diclofenac, enalapril. pharmacodynamic antagonism. Avoid or Use Alternate Drug. Coadministration may result in a significant decrease in renal function. NSAIDs may diminish the antihypertensive effect of ACE inhibitors. The mechanism of these interactions is likely related to the ability of NSAIDs to reduce the synthesis of vasodilating renal prostaglandins
fluconazole + ondansetron	2	fluconazole and ondansetron both increase QTc interval. Avoid or Use Alternate Drug. Avoid with congenital long QT syndrome; ECG monitoring recommended with concomitant medications that prolong QT interval, electrolyte abnormalities, CHF, or bradyarrhythmias. Combination may increase ondansetron levels

levofloxacin + sodium bicarbonate	2	sodium bicarbonate decreases levels of levofloxacin by inhibition of GI absorption. Applies only to oral form of both agents. Avoid or Use Alternate Drug. Separate by 2 hours
amiodarone + escitalopram	1	escitalopram increases toxicity of amiodarone by QTc interval. Avoid or Use Alternate Drug
amiodarone + indapamide	1	amiodarone and indapamide both increase QTc interval. Avoid or Use Alternate Drug
apixaban + rivaroxaban	1	rivaroxaban and apixaban both increase anticoagulation. Avoid or Use Alternate Drug
apixaban + warfarin	1	apixaban increases effects of warfarin by anticoagulation. Avoid or Use Alternate Drug. Avoid combined use once INR is established in the desired therapeutic range
aspirin + ketorolac	1	aspirin, ketorolac. Either increases toxicity of the other by pharmacodynamic synergism. Contraindicated
bisoprolol + nebivolol	1	bisoprolol and nebivolol both increase anti-hypertensive channel blocking. Avoid or Use Alternate Drug
bisoprolol + verapamil	1	bisoprolol and verapamil both increase anti-hypertensive channel blocking. Modify Therapy/Monitor Closely
captopril + diclofenac	1	captopril, diclofenac. Either increases toxicity of the other by Other (see comment). Use Caution/Monitor. Comment: May result in renal function deterioration, particularly in elderly or volume depleted individuals
captopril + ketorolac	1	captopril, ketorolac. Either increases toxicity of the other by Other (see comment). Use Caution/Monitor. Comment: May result in renal function deterioration, particularly in elderly or volume depleted individuals
captopril + telmisartan	1	telmisartan, captopril. Either increases toxicity of the other by pharmacodynamic synergism. Avoid or Use Alternate Drug. Dual blockade of renin-angiotensin system increases risks of hypotension, hyperkalemia, and renal impairment
carbamazepine + prednisolone	1	carbamazepine will decrease the level or effect of prednisolone by affecting hepatic/intestinal enzyme CYP3A4 metabolism. Avoid or Use Alternate Drug
ceftriaxone + fondaparinux	1	ceftriaxone increases effects of fondaparinux by anticoagulation. Avoid or Use Alternate Drug. cephalosporins may decrease prothrombin activity
clarithromycin + dexamethasone	1	clarithromycin will increase the level or effect of dexamethasone by affecting hepatic/intestinal enzyme CYP3A4 metabolism. Avoid or Use Alternate Drug
clarithromycin + enoxaparin	1	clarithromycin increases effects of enoxaparin by decreasing metabolism. Avoid or Use Alternate Drug
clopidogrel + fondaparinux	1	fondaparinux, clopidogrel. Either increases effects of the other by pharmacodynamic synergism. Contraindicated. Enhanced risk of hemorrhage
diclofenac + fosinopril	1	diclofenac, fosinopril. pharmacodynamic antagonism. Avoid or Use Alternate Drug. Coadministration may result in a significant decrease in renal function. NSAIDs may diminish the antihypertensive effect of ACE inhibitors. The mechanism of these interactions is likely related to the ability of NSAIDs to reduce the synthesis of vasodilating renal prostaglandins
diclofenac + ketorolac	1	diclofenac, ketorolac. Either increases toxicity of the other by pharmacodynamic synergism. Contraindicated
diclofenac + lisinopril	1	diclofenac, lisinopril. pharmacodynamic antagonism. Avoid or Use Alternate Drug. Coadministration may result in a significant decrease in renal function. NSAIDs may diminish the antihypertensive effect of ACE inhibitors. The mechanism of these interactions is likely related to the ability of NSAIDs to reduce the synthesis of vasodilating renal prostaglandins

diclofenac + methotrexate	1	diclofenac increases levels of methotrexate by decreasing renal clearance. Avoid or Use Alternate Drug. Concomitant administration of NSAIDs with high dose methotrexate has been reported to elevate and prolong serum methotrexate levels, resulting in deaths from severe hematologic and GI toxicity. NSAIDs may reduce tubular secretion of methotrexate and enhance toxicity
enalapril + ketorolac	1	enalapril, ketorolac. Either increases toxicity of the other by Other (see comment). Use Caution/Monitor. Comment: May result in renal function deterioration, particularly in elderly or volume depleted individuals
enalapril + losartan	1	losartan, enalapril. Either increases toxicity of the other by pharmacodynamic synergism. Avoid or Use Alternate Drug. Dual blockade of renin-angiotensin system increases risks of hypotension, hyperkalemia, and renal impairment
enoxaparin + heparin	1	enoxaparin and heparin both increase anticoagulation. Avoid or Use Alternate Drug
eplerenone + potassium	1	potassium chloride, eplerenone. Mechanism: pharmacodynamic synergism. Contraindicated. Hyperkalemia
formoterol + indapamide	1	formoterol and indapamide both increase QTc interval. Avoid or Use Alternate Drug
fosinopril + telmisartan	1	telmisartan, fosinopril. Either increases toxicity of the other by pharmacodynamic synergism. Avoid or Use Alternate Drug. Dual blockade of renin-angiotensin system increases risks of hypotension, hyperkalemia, and renal impairment
heparin + warfarin	1	heparin increases effects of warfarin by anticoagulation. Avoid or Use Alternate Drug. Avoid combined use once INR is established in the desired therapeutic range
ketorolac + meloxicam	1	ketorolac and meloxicam both increase anticoagulation. Use Caution/Monitor
lisinopril + losartan	2	losartan, lisinopril. Either increases toxicity of the other by pharmacodynamic synergism. Avoid or Use Alternate Drug. Dual blockade of renin-angiotensin system increases risks of hypotension, hyperkalemia, and renal impairment
lisinopril + meloxicam	1	lisinopril, meloxicam. Either increases toxicity of the other by Other (see comment). Use Caution/Monitor. Comment: May result in renal function deterioration, particularly in elderly or volume depleted individuals
lisinopril + valsartan	1	valsartan, lisinopril. Either increases toxicity of the other by pharmacodynamic synergism. Avoid or Use Alternate Drug. Dual blockade of renin-angiotensin system increases risks of hypotension, hyperkalemia, and renal impairment
metoprolol + nebivolol	1	metoprolol and nebivolol both increase anti-hypertensive channel blocking. Avoid or Use Alternate Drug
mifepristone + quinine	1	mifepristone will increase the level or effect of quinine by affecting hepatic/intestinal enzyme CYP3A4 metabolism. Avoid or Use Alternate Drug
propranolol + sotalol	1	propranolol and sotalol both increase anti-hypertensive channel blocking. Avoid or Use Alternate Drug

Note: Impact of drug–drug interactions associated with the combinations “aspirin + captopril” and “aspirin + enalapril” may be considered insignificant due to the use of low-dose aspirin in the majority of cases. Administration of aspirin at doses less than 300 mg per day has little effect on the effectiveness of captopril and enalapril. Administration of aspirin in higher doses reduces the effectiveness of captopril and enalapril.

Table S6. Drug combinations resulting in potential monitor-closely drug–drug interactions in the list of taken medications in patients with cardiovascular diseases according to data derived from the electronic health records (n = 1030) established in 2018–2023.

Drug combinations	n	Potential monitor-closely drug-drug interactions (T-list)
aspirin + bisoprolol	282	aspirin decreases effects of bisoprolol by pharmacodynamic antagonism. Use Caution/Monitor. Long term (>1 wk) NSAID use. NSAIDs decrease prostaglandin synthesis
aspirin + losartan	120	aspirin decreases effects of losartan by pharmacodynamic antagonism. Modify Therapy/Monitor Closely. NSAIDs decrease synthesis of vasodilating renal prostaglandins, and thus affect fluid homeostasis and may diminish antihypertensive effect
aspirin + spironolactone	120	aspirin decreases effects of spironolactone by unspecified interaction mechanism. Use Caution/Monitor. When used concomitantly, spironolactone dose may need to be titrated to higher maintenance dose and the patient should be observed closely to determine if the desired effect is obtained
aspirin + metoprolol	88	aspirin decreases effects of metoprolol by pharmacodynamic antagonism. Use Caution/Monitor. Long term (>1 wk) NSAID use. NSAIDs decrease prostaglandin synthesis
bisoprolol + losartan	72	bisoprolol, losartan. Mechanism: pharmacodynamic synergism. Use Caution/Monitor. Risk of fetal compromise if given during pregnancy
bisoprolol + torsemide	70	bisoprolol increases and torsemide decreases serum potassium. Effect of interaction is not clear, use caution. Use Caution/Monitor
spironolactone + torsemide	69	spironolactone increases and torsemide decreases serum potassium. Effect of interaction is not clear, use caution. Modify Therapy/Monitor Closely
bisoprolol + spironolactone	64	bisoprolol and spironolactone both increase serum potassium. Modify Therapy/Monitor Closely
digoxin + spironolactone	57	spironolactone, digoxin. Mechanism: decreasing renal clearance. Use Caution/Monitor. False digoxin assay results may be obtained
		spironolactone increases levels of digoxin by Other (see comment). Use Caution/Monitor. Comment: Spironolactone may cause false elevation of digoxin assay
		spironolactone and digoxin both increase serum potassium. Modify Therapy/Monitor Closely
aspirin + torsemide	56	aspirin increases and torsemide decreases serum potassium. Effect of interaction is not clear, use caution. Use Caution/Monitor
aspirin + clopidogrel	53	aspirin, clopidogrel. Either increases toxicity of the other by pharmacodynamic synergism. Use Caution/Monitor. The need for simultaneous use of low-dose aspirin and anticoagulant or antiplatelet agents are common for patients with cardiovascular disease; monitor closely
amlodipine + bisoprolol	51	bisoprolol, amlodipine. Either increases effects of the other by pharmacodynamic synergism. Use Caution/Monitor. Both drugs lower blood pressure
aspirin + lisinopril	48	lisinopril, aspirin. Either increases toxicity of the other by Other (see comment). Use Caution/Monitor. Comment: May result in renal function deterioration, particularly with high dose aspirin, in elderly or volume depleted individuals
amiodarone + atorvastatin	42	amiodarone will increase the level or effect of atorvastatin by P-glycoprotein (MDR1) efflux transporter. Use Caution/Monitor

aspirin + perindopril	40	perindopril, aspirin. Either increases toxicity of the other by Other (see comment). Use Caution/Monitor. Comment: May result in renal function deterioration, particularly with high doses of aspirin,in elderly or volume depleted individuals
furosemide + spironolactone	38	spironolactone increases and furosemide decreases serum potassium. Effect of interaction is not clear, use caution. Modify Therapy/Monitor Closely
aspirin + nitroglycerin	36	aspirin increases effects of nitroglycerin sublingual by additive vasodilation. Use Caution/Monitor. Vasodilatory and hemodynamic effects of NTG may be enhanced by coadministration with aspirin (additive effect desirable for emergent treatment)
amiodarone + bisoprolol	34	amiodarone, bisoprolol. Mechanism: pharmacodynamic synergism. Use Caution/Monitor. Risk of cardiotoxicity with bradycardia
amiodarone + losartan	32	amiodarone will increase the level or effect of losartan by affecting hepatic enzyme CYP2C9/10 metabolism. Use Caution/Monitor. May inhibit the conversion of losartan to its active metabolite E-3174. Importance of interaction not established; monitor individual therapeutic response to determine losartan dosage
aspirin + captopril *	32	captopril, aspirin. Either increases toxicity of the other by Other (see comment). Use Caution/Monitor. Comment: May result in renal function deterioration, particularly with high dose aspirin, elderly or volume depleted individuals
aspirin + indapamide	32	aspirin increases and indapamide decreases serum potassium. Effect of interaction is not clear, use caution. Use Caution/Monitor
bisoprolol + digoxin	32	bisoprolol increases effects of digoxin by pharmacodynamic synergism. Use Caution/Monitor. Enhanced bradycardia
aspirin + heparin	30	aspirin, heparin. Either increases toxicity of the other by anticoagulation. Use Caution/Monitor. The need for simultaneous use of low-dose aspirin and anticoagulant or antiplatelet agents are common for patients with cardiovascular disease; monitor closely
aspirin + furosemide	29	aspirin increases and furosemide decreases serum potassium. Effect of interaction is not clear, use caution. Use Caution/Monitor
		digoxin increases and torsemide decreases serum potassium. Effect of interaction is not clear, use caution. Use Caution/Monitor
losartan + torsemide	28	losartan increases and torsemide decreases serum potassium. Effect of interaction is not clear, use caution. Use Caution/Monitor
bisoprolol + indapamide	27	bisoprolol increases and indapamide decreases serum potassium. Effect of interaction is not clear, use caution. Use Caution/Monitor
lisinopril + spironolactone	26	lisinopril, spironolactone. Mechanism: pharmacodynamic synergism. Use Caution/Monitor. Risk of hyperkalemia
aspirin + ticagrelor	25	aspirin, ticagrelor. Other (see comment). Use Caution/Monitor. Comment: Maintenance doses of aspirin above 100 mg decreases effectiveness of ticagrelor. Therefore, after the initial loading dose of aspirin (usually 325 mg), use ticagrelor with a maintenance dose of aspirin of 75-100 mg
lisinopril + torsemide	25	lisinopril, torsemide. Mechanism: pharmacodynamic synergism. Use Caution/Monitor. Risk of acute hypotension, renal insufficiency
aspirin + telmisartan	24	aspirin decreases effects of telmisartan by pharmacodynamic antagonism. Modify Therapy/Monitor Closely. NSAIDs decrease synthesis of vasodilating renal prostaglandins, and thus affect fluid homeostasis and may diminish antihypertensive effect

bisoprolol + furosemide	24	bisoprolol increases and furosemide decreases serum potassium. Effect of interaction is not clear, use caution. Use Caution/Monitor
losartan + metoprolol	24	losartan and metoprolol both increase serum potassium. Use Caution/Monitor
aspirin + enalapril *	22	enalapril, aspirin. Either increases toxicity of the other by Other (see comment). Use Caution/Monitor. Comment: May result in renal function deterioration, particularly with high dose aspirin, in elderly or volume depleted individuals
aspirin + enoxaparin	22	aspirin, enoxaparin. Either increases toxicity of the other by pharmacodynamic synergism. Use Caution/Monitor. The need for simultaneous use of low-dose aspirin and anticoagulant or antiplatelet agents are common for patients with cardiovascular disease; monitor closely
metoprolol + spironolactone	22	metoprolol and spironolactone both increase serum potassium. Modify Therapy/Monitor Closely
perindopril + torsemide	22	perindopril, torsemide. Mechanism: pharmacodynamic synergism. Use Caution/Monitor. Risk of acute hypotension, renal insufficiency
losartan + spironolactone	21	losartan and spironolactone both increase serum potassium. Modify Therapy/Monitor Closely
amiodarone + rivaroxaban	19	amiodarone increases levels of rivaroxaban by affecting hepatic/intestinal enzyme CYP3A4 metabolism. Use Caution/Monitor. Patients with renal impairment receiving rivaroxaban with drugs that are combined P-gp and weak or moderate CYP3A4 inhibitors may have significant increases in exposure compared with patients with normal renal function and no inhibitor use, since both pathways of rivaroxaban elimination are affected. Since these increases may increase bleeding risk, use rivaroxaban in this situation only if the potential benefit justifies the potential risk
aspirin + insulin	19	aspirin increases effects of insulin detemir by pharmacodynamic synergism. Modify Therapy/Monitor Closely. Coadministration of insulin with high doses of salicylates (3 g/day or more) may increase risk for hypoglycemia. Insulin dose adjustment and increased frequency of glucose monitoring may be required
metformin + torsemide	19	torsemide decreases effects of metformin by pharmacodynamic antagonism. Use Caution/Monitor
digoxin + furosemide	18	digoxin increases and furosemide decreases serum potassium. Effect of interaction is not clear, use caution. Use Caution/Monitor
digoxin + metoprolol	18	metoprolol increases effects of digoxin by pharmacodynamic synergism. Use Caution/Monitor. Enhanced bradycardia
		metoprolol and digoxin both increase serum potassium. Use Caution/Monitor
amlodipine + nitroglycerin	17	amlodipine, nitroglycerin sublingual. Either increases toxicity of the other by additive vasodilation. Modify Therapy/Monitor Closely. Marked orthostatic hypotension reported with concomitant use
metoprolol + torsemide	17	metoprolol increases and torsemide decreases serum potassium. Effect of interaction is not clear, use caution. Use Caution/Monitor
atorvastatin + valsartan	16	atorvastatin will increase the level or effect of valsartan by Other (see comment). Use Caution/Monitor. The results from an in vitro study with human liver tissue indicate that valsartan is a substrate of the hepatic uptake transporter OATP1B1; coadministration with OATP1B1 inhibitors may increase valsartan systemic exposure

bisoprolol + telmisartan	16	bisoprolol, telmisartan. Mechanism: pharmacodynamic synergism. Use Caution/Monitor. Risk of fetal compromise if given during pregnancy
losartan + omeprazole	16	omeprazole will increase the level or effect of losartan by affecting hepatic enzyme CYP2C9/10 metabolism. Use Caution/Monitor. May inhibit the conversion of losartan to its active metabolite E-3174. Importance of interaction not established; monitor individual therapeutic response to determine losartan dosage
aspirin + rivaroxaban	15	aspirin, rivaroxaban. Either increases toxicity of the other by anticoagulation. Use Caution/Monitor. Both drugs have the potential to cause bleeding. The need for simultaneous use of low-dose aspirin (<100 mg/day) with anticoagulants are common for patients with cardiovascular disease, but may result in increased bleeding; monitor closely. Promptly evaluate any signs or symptoms of blood loss if treated concomitantly with low-dose aspirin. Avoid coadministration with chronic use of higher dose aspirin
captopril + torsemide	15	captopril, torsemide. Either increases toxicity of the other by Mechanism: pharmacodynamic synergism. Use Caution/Monitor. Risk of acute hypotension, renal insufficiency. Monitor blood pressure and renal function
clopidogrel + rivaroxaban	15	rivaroxaban, clopidogrel. Other (see comment). Use Caution/Monitor. Comment: Avoid concurrent administration of clopidogrel with rivaroxaban unless the benefit outweighs the risk of increased bleeding
aspirin + azilsartan	14	aspirin, azilsartan. Either increases toxicity of the other by Other (see comment). Use Caution/Monitor. Comment: May result in renal function deterioration, particularly in elderly or volume depleted individuals
		aspirin decreases effects of azilsartan by pharmacodynamic antagonism. Modify Therapy/Monitor Closely. NSAIDs decrease synthesis of vasodilating renal prostaglandins, and thus affect fluid homeostasis and may diminish antihypertensive effect
aspirin + fosinopril	14	fosinopril, aspirin. Either increases toxicity of the other by Other (see comment). Use Caution/Monitor. Comment: May result in renal function deterioration, particularly with high dose aspirin, in elderly or volume depleted individuals
furosemide + torsemide	14	furosemide and torsemide both decrease serum potassium. Use Caution/Monitor
enalapril + spironolactone	13	enalapril, spironolactone. Mechanism: pharmacodynamic synergism. Use Caution/Monitor. Risk of hyperkalemia
fosinopril + torsemide	13	fosinopril, torsemide. Mechanism: pharmacodynamic synergism. Use Caution/Monitor. Risk of acute hypotension, renal insufficiency
aspirin + candesartan	12	aspirin decreases effects of candesartan by pharmacodynamic antagonism. Modify Therapy/Monitor Closely. NSAIDs decrease synthesis of vasodilating renal prostaglandins, and thus affect fluid homeostasis and may diminish antihypertensive effect
aspirin + hydrochlorothiazide	12	aspirin increases and hydrochlorothiazide decreases serum potassium. Effect of interaction is not clear, use caution. Use Caution/Monitor
aspirin + valsartan	12	aspirin decreases effects of valsartan by pharmacodynamic antagonism. Modify Therapy/Monitor Closely. NSAIDs decrease synthesis of vasodilating renal prostaglandins, and thus affect fluid homeostasis and may diminish antihypertensive effect
atorvastatin + dexamethasone	12	atorvastatin will increase the level or effect of dexamethasone by P-glycoprotein (MDR1) efflux transporter. Use Caution/Monitor

enalapril + furosemide	12	enalapril, furosemide. Mechanism: pharmacodynamic synergism. Use Caution/Monitor. Risk of acute hypotension, renal insufficiency
eplerenone + perindopril	12	perindopril, eplerenone. Mechanism: pharmacodynamic synergism. Use Caution/Monitor. Risk of hyperkalemia
lisinopril + metformin	12	lisinopril increases toxicity of metformin by unspecified interaction mechanism. Use Caution/Monitor. Increases risk for hypoglycemia and lactic acidosis
perindopril + spironolactone	12	perindopril, spironolactone. Mechanism: pharmacodynamic synergism. Use Caution/Monitor. Risk of hyperkalemia
bisoprolol + hydrochlorothiazide	11	bisoprolol increases and hydrochlorothiazide decreases serum potassium. Effect of interaction is not clear, use caution. Use Caution/Monitor
enoxaparin + spironolactone	11	spironolactone, enoxaparin. Either increases toxicity of the other by serum potassium. Use Caution/Monitor. Both drugs may increase serum potassium levels
furosemide + metoprolol	11	metoprolol increases and furosemide decreases serum potassium. Effect of interaction is not clear, use caution. Use Caution/Monitor
rosuvastatin + warfarin	11	rosuvastatin increases effects of warfarin by anticoagulation. Use Caution/Monitor
amiodarone + metoprolol	10	amiodarone will increase the level or effect of metoprolol by affecting hepatic enzyme CYP2D6 metabolism. Use Caution/Monitor. Monitor cardiac function carefully and observe for signs of bradycardia or heart block when amiodarone and a beta adrenergic blocker are coadministered. Amiodarone should be used with caution in patients receiving a beta adrenergic blocker, particularly if there is suspicion of underlying dysfunction of the sinus node, such as bradycardia or sick sinus syndrome, or if there is partial AV block
amiodarone + metoprolol	10	amiodarone, metoprolol. Mechanism: pharmacodynamic synergism. Use Caution/Monitor. Risk of cardiotoxicity with bradycardia
amlodipine + metformin	10	amlodipine decreases effects of metformin by pharmacodynamic antagonism. Use Caution/Monitor. Patient should be closely observed for loss of blood glucose control; when drugs are withdrawn from a patient receiving metformin, patient should be observed closely for hypoglycemia
aspirin + carvedilol	10	aspirin decreases effects of carvedilol by pharmacodynamic antagonism. Use Caution/Monitor. Long term (>1 wk) NSAID use. NSAIDs decrease prostaglandin synthesis
aspirin + digoxin	10	aspirin and digoxin both increase serum potassium. Use Caution/Monitor
atorvastatin + digoxin	10	atorvastatin will increase the level or effect of digoxin by P-glycoprotein (MDR1) efflux transporter. Use Caution/Monitor
bisoprolol + candesartan	10	bisoprolol, candesartan. Mechanism: pharmacodynamic synergism. Use Caution/Monitor. Risk of fetal compromise if given during pregnancy
bisoprolol + potassium	10	bisoprolol and potassium chloride both increase serum potassium. Modify Therapy/Monitor Closely
digoxin + omeprazole	10	omeprazole will increase the level or effect of digoxin by increasing gastric pH. Applies only to oral form of both agents. Avoid or Use Alternate Drug
digoxin + omeprazole	10	omeprazole increases toxicity of digoxin by Other (see comment). Use Caution/Monitor. Comment: Prolonged use of PPIs may cause hypomagnesemia and increase risk for digoxin toxicity

aspirin + potassium	9	aspirin and potassium chloride both increase serum potassium. Modify Therapy/Monitor Closely
aspirin + sacubitril/valsartan	9	aspirin decreases effects of sacubitril/valsartan by pharmacodynamic antagonism. Modify Therapy/Monitor Closely. NSAIDs decrease synthesis of vasodilating renal prostaglandins, and thus affect fluid homeostasis and may diminish antihypertensive effect
atorvastatin + dabigatran	9	atorvastatin will increase the level or effect of dabigatran by P-glycoprotein (MDR1) efflux transporter. Use Caution/Monitor. Atrial fibrillation: Avoid coadministering dabigatran with P-gp inhibitors if CrCl <30 mL/min. DVT/PE treatment: Avoid coadministering dabigatran with P-gp inhibitors if CrCl <50 mL/min
budesonide + verapamil	9	budesonide will decrease the level or effect of verapamil by affecting hepatic/intestinal enzyme CYP3A4 metabolism. Use Caution/Monitor
captopril + indapamide	9	indapamide, captopril. Either increases effects of the other by pharmacodynamic synergism. Use Caution/Monitor. Both drugs lower blood pressure. Increased risk of nephrotoxicity. Monitor blood pressure and renal function
clopidogrel + pantoprazole	9	pantoprazole decreases effects of clopidogrel by affecting hepatic enzyme CYP2C19 metabolism. Use Caution/Monitor. Clopidogrel efficacy may be reduced by drugs that inhibit CYP2C19. Inhibition of platelet aggregation by clopidogrel is entirely due to the active clopidogrel metabolite. Clopidogrel is metabolized in part by CYP2C19. Pantoprazole prescribing information state that coadministration with clopidogrel had no clinically important effect on exposure to clopidogrel active metabolite; no dose adjustment of clopidogrel is required
enalapril + insulin	9	enalapril increases effects of insulin NPH by pharmacodynamic synergism. Use Caution/Monitor.
		enalapril increases effects of insulin detemir by pharmacodynamic synergism. Use Caution/Monitor
furosemide + losartan	9	losartan increases and furosemide decreases serum potassium. Effect of interaction is not clear, use caution. Use Caution/Monitor
furosemide + potassium	9	potassium increases and furosemide decreases serum potassium. Effect of interaction is not clear, use caution. Modify Therapy/Monitor Closely
acetazolamide + aspirin	8	acetazolamide, aspirin. Mechanism: passive renal tubular reabsorption due to increased pH. Use Caution/Monitor. Salicylate levels increased at moderate doses; risk of CNS toxicity. Salicylate levels decreased at large doses (d/t increased renal excretion of unchanged salicylic acid)
		acetazolamide, aspirin. Either increases levels of the other by Other (see comment). Use Caution/Monitor. Comment: Carbonic anhydrase inhibitors (CAIs) and salicylates inhibit each other's renal tubular secretion, resulting in increased plasma levels. CAIs also shift salicylates from plasma to the CNS, leading to potential neurotoxicity
amiodarone + warfarin	8	amiodarone will increase the level or effect of warfarin by affecting hepatic enzyme CYP2C9/10 metabolism. Modify Therapy/Monitor Closely. Coadministration increases INR by 100% after 3-4 days. Reduce warfarin dose by one-third to one-half and monitor INR
apixaban + aspirin	8	aspirin and apixaban both increase anticoagulation. Modify Therapy/Monitor Closely. Both drugs have the potential to cause bleeding. The need for simultaneous use of low-dose aspirin (<100 mg/day) with anticoagulants are common for patients with cardiovascular disease, but may result in increased bleeding; monitor closely. Promptly evaluate any signs or symptoms of

		blood loss if treated concomitantly with low-dose aspiriin. Avoid coadministration with chronic use of higher dose aspirin. In 1 trial (APPRAISE-2), therapy was terminated because of significantly increased bleeding when apixaban was administered with dual antiplatelet therapy (eg, aspirin plus clopidogrel) compared with single antiplatelet treatment
aspirin + warfarin	8	aspirin increases effects of warfarin by anticoagulation. Modify Therapy/Monitor Closely. Avoid coadministration of chronic high-dose aspirin. Aspirin's antiplatelet properties may increase anticoagulation effect of warfarin. The need for simultaneous use of low-dose aspirin and warfarin is common for patients with cardiovascular disease
atorvastatin + nifedipine	8	nifedipine will increase the level or effect of atorvastatin by affecting hepatic/intestinal enzyme CYP3A4 metabolism. Use Caution/Monitor
		nifedipine will decrease the level or effect of atorvastatin by P-glycoprotein (MDR1) efflux transporter. Use Caution/Monitor
bisoprolol + felodipine	8	bisoprolol and felodipine both increase anti-hypertensive channel blocking. Modify Therapy/Monitor Closely
bisoprolol + valsartan	8	bisoprolol, valsartan. Mechanism: pharmacodynamic synergism. Use Caution/Monitor. Risk of fetal compromise if given during pregnancy
clopidogrel + heparin	8	heparin, clopidogrel. Either increases effects of the other by pharmacodynamic synergism. Modify Therapy/Monitor Closely. Enhanced risk of hemorrhage; additive effects are intended when both drugs are prescribed as indicated for ACS
enalapril + metformin	8	enalapril increases toxicity of metformin by unspecified interaction mechanism. Use Caution/Monitor. Increases risk for hypoglycemia and lactic acidosis
enalapril + torsemide	8	enalapril, torsemide. Mechanism: pharmacodynamic synergism. Use Caution/Monitor. Risk of acute hypotension, renal insufficiency
indapamide + metoprolol	8	metoprolol increases and indapamide decreases serum potassium. Effect of interaction is not clear, use caution. Use Caution/Monitor
insulin + lisinopril	8	lisinopril increases effects of insulin detemir by pharmacodynamic synergism. Use Caution/Monitor
insulin + metformin	8	metformin, insulin regular human. Either increases effects of the other by pharmacodynamic synergism. Use Caution/Monitor. Antidiabetic agents are often used in combination; dosage adjustments may be required when initiating or discontinuing antidiabetic agents
losartan + warfarin	8	losartan will increase the level or effect of warfarin by affecting hepatic enzyme CYP2C9/10 metabolism. Use Caution/Monitor
amiodarone + metformin	7	amiodarone will increase the level or effect of metformin by basic (cationic) drug competition for renal tubular clearance. Use Caution/Monitor
aspirin + prednisolone	7	aspirin, prednisolone. Either increases toxicity of the other by pharmacodynamic synergism. Use Caution/Monitor. Increased risk of GI ulceration
bisoprolol + metoprolol	7	bisoprolol and metoprolol both increase serum potassium. Use Caution/Monitor
captopril + spironolactone	7	captopril, spironolactone. Either increases toxicity of the other by Mechanism: pharmacodynamic synergism. Use Caution/Monitor. Both drugs lower blood pressure. Risk of hyperkalemia. Monitor blood pressure and potassium

clopidogrel + enoxaparin	7	enoxaparin, clopidogrel. Either increases effects of the other by pharmacodynamic synergism. Modify Therapy/Monitor Closely. Enhanced risk of hemorrhage; additive effects are intended when both drugs are prescribed as indicated for ACS
dexamethasone + enoxaparin	7	dexamethasone, enoxaparin. Other (see comment). Use Caution/Monitor. Comment: Corticosteroids may decrease anticoagulant effects by increasing blood coagulability; conversely, they may impair vascular integrity, thus increasing bleeding risk. Monitor INR closely
fosinopril + spironolactone	7	fosinopril, spironolactone. Mechanism: pharmacodynamic synergism. Use Caution/Monitor. Risk of hyperkalemia
indapamide + spironolactone	7	spironolactone increases and indapamide decreases serum potassium. Effect of interaction is not clear, use caution. Modify Therapy/Monitor Closely
indapamide + torsemide	7	torsemide and indapamide both decrease serum potassium. Use Caution/Monitor
omeprazole + warfarin	7	omeprazole will increase the level or effect of warfarin by Other (see comment). Use Caution/Monitor. Warfarin's less potent R-enantiomer is metabolized in part by CYP3A4 (and also CYP1A2 and CYP2C19). Monitor INR more frequently if coadministered with inhibitors of these isoenzymes and adjust warfarin dose if needed
amiodarone + dexamethasone	6	amiodarone will increase the level or effect of dexamethasone by P-glycoprotein (MDR1) efflux transporter. Use Caution/Monitor
amiodarone + nebivolol	6	amiodarone will increase the level or effect of nebivolol by affecting hepatic enzyme CYP2D6 metabolism. Use Caution/Monitor. Monitor cardiac function carefully and observe for signs of bradycardia or heart block when amiodarone and a beta adrenergic blocker are coadministered. Amiodarone should be used with caution in patients receiving a beta adrenergic blocker, particularly if there is suspicion of underlying dysfunction of the sinus node, such as bradycardia or sick sinus syndrome, or if there is partial AV block
amiodarone + nebivolol	6	amiodarone, nebivolol. Mechanism: pharmacodynamic synergism. Use Caution/Monitor. Risk of cardiotoxicity with bradycardia
aspirin + sotalol	6	aspirin decreases effects of sotalol by pharmacodynamic antagonism. Use Caution/Monitor. Long term (>1 wk) NSAID use. NSAIDs decrease prostaglandin synthesis
atorvastatin + budesonide	6	atorvastatin will increase the level or effect of budesonide by P-glycoprotein (MDR1) efflux transporter. Use Caution/Monitor
atorvastatin + ranolazine	6	ranolazine will increase the level or effect of atorvastatin by P-glycoprotein (MDR1) efflux transporter. Use Caution/Monitor
		ranolazine increases toxicity of atorvastatin by Other (see comment). Modify Therapy/Monitor Closely. Comment: OATP1B1 inhibitors may increase risk of myopathy
atorvastatin + sacubitril/valsartan	6	atorvastatin will increase the level or effect of sacubitril/valsartan by Other (see comment). Use Caution/Monitor. The results from an in vitro study with human liver tissue indicate that valsartan is a substrate of the hepatic uptake transporter OATP1B1; coadministration with OATP1B1 inhibitors may increase valsartan systemic exposure
bisoprolol + formoterol	6	bisoprolol decreases effects of formoterol by pharmacodynamic antagonism. Use Caution/Monitor
		bisoprolol increases and formoterol decreases serum potassium. Effect of interaction is not clear, use caution. Use Caution/Monitor

bisoprolol + meloxicam	6	bisoprolol and meloxicam both increase serum potassium. Use Caution/Monitor
bisoprolol + sacubitril/valsartan	6	bisoprolol, sacubitril/valsartan. Mechanism: pharmacodynamic synergism. Use Caution/Monitor. Risk of fetal compromise if given during pregnancy
budesonide + omeprazole	6	omeprazole decreases effects of budesonide by increasing gastric pH. Applies only to oral form of both agents. Modify Therapy/Monitor Closely. Enteric-coated budesonide dissolves at pH >5.5. Also, dissolution of extended-release budesonide tablets is pH dependent. Coadministration with drugs that increase gastric pH may cause these budesonide products to prematurely dissolve, and possibly affect release properties and absorption of the drug in the duodenum
empagliflozin + torsemide	6	empagliflozin, torsemide. Either increases effects of the other by pharmacodynamic synergism. Use Caution/Monitor. Coadministration of empagliflozin with diuretics results in increased urine volume and frequency of voids, which might enhance the potential for volume depletion
indapamide + losartan	6	losartan increases and indapamide decreases serum potassium. Effect of interaction is not clear, use caution. Use Caution/Monitor
insulin + losartan	6	losartan increases effects of insulin glargine by unspecified interaction mechanism. Use Caution/Monitor. Concomitant use of insulin and ARBs may require insulin dosage adjustment and increased glucose monitoring
metformin + perindopril	6	perindopril increases toxicity of metformin by unspecified interaction mechanism. Use Caution/Monitor. Increases risk for hypoglycemia and lactic acidosis
metoprolol + sacubitril/valsartan	6	metoprolol, sacubitril/valsartan. Mechanism: pharmacodynamic synergism. Use Caution/Monitor. Risk of fetal compromise if given during pregnancy
potassium + torsemide	6	potassium chloride increases and torsemide decreases serum potassium. Effect of interaction is not clear, use caution. Modify Therapy/ Monitor Closely
amiodarone + captopril	5	amiodarone, captopril. Either increases effects of the other by pharmacodynamic synergism. Use Caution/Monitor. Both drugs lower blood pressure. Monitor blood pressure
amlodipine + sotalol	5	sotalol and amlodipine both increase anti-hypertensive channel blocking. Modify Therapy/Monitor Closely
aspirin + chlorthalidone	5	aspirin increases and chlorthalidone decreases serum potassium. Effect of interaction is not clear, use caution. Use Caution/Monitor
atorvastatin + prednisolone	5	atorvastatin will increase the level or effect of prednisolone by P-glycoprotein (MDR1) efflux transporter. Use Caution/Monitor
digoxin + enalapril	5	enalapril increases levels of digoxin by unspecified interaction mechanism. Use Caution/Monitor
digoxin + potassium	5	potassium chloride and digoxin both increase serum potassium. Modify Therapy/Monitor Closely
empagliflozin + spironolactone	5	empagliflozin, spironolactone. Either increases effects of the other by pharmacodynamic synergism. Use Caution/Monitor. Coadministration of empagliflozin with diuretics results in increased urine volume and frequency of voids, which might enhance the potential for volume depletion
enalapril + enoxaparin	5	enoxaparin increases toxicity of enalapril by Other (see comment). Use Caution/Monitor. Comment: Low molecular weight heparins may suppress adrenal aldosterone secretion, which can potentially cause hyperkalemia

enoxaparin + losartan	5	enoxaparin increases toxicity of losartan by Other (see comment). Use Caution/Monitor. Comment: Low molecular weight heparins may suppress adrenal aldosterone secretion, which can potentially cause hyperkalemia
furosemide + lisinopril	5	lisinopril, furosemide. Mechanism: pharmacodynamic synergism. Use Caution/Monitor. Risk of acute hypotension, renal insufficiency
heparin + lisinopril	5	heparin increases toxicity of lisinopril by Other (see comment). Use Caution/Monitor. Comment: Low molecular weight heparins may suppress adrenal aldosterone secretion, which can potentially cause hyperkalemia
Indapamide + losartan	5	losartan increases and indapamide decreases serum potassium. Effect of interaction is not clear, use caution. Use Caution/Monitor
insulin+ metformin	5	metformin, insulin aspart. Either increases effects of the other by pharmacodynamic synergism. Use Caution/Monitor. Antidiabetic agents are often used in combination; dosage adjustments may be required when initiating or discontinuing antidiabetic agents
		metformin, insulin glargine. Either increases effects of the other by pharmacodynamic synergism. Use Caution/Monitor. Antidiabetic agents are often used in combination; do sage adjustments may be required when initiating or discontinuing antidiabetic agents
ipratropium + tiotropium	5	ipratropium and tiotropium both decrease cholinergic effects/transmission. Use Caution/Monitor. Due to the poor systemic absorption of ipratropium, interaction unlikely at regularly recommended dosages
levofloxacin + prednisolone	5	prednisolone and levofloxacin both increase Other (see comment). Use Caution/Monitor. Coadministration of quinolone antibiotics and corticosteroids may increase risk of tendon rupture
metoprolol + nifedipine	5	metoprolol and nifedipine both increase anti-hypertensive channel blocking. Modify Therapy/Monitor Closely
amiodarone + tamsulosin	4	amiodarone increases levels of tamsulosin by affecting hepatic/intestinal enzyme CYP3A4 metabolism. Use Caution/Monitor. Dose reduction may be needed for coadministered drugs that are predominantly metabolized by CYP3A
		amiodarone increases levels of tamsulosin by affecting hepatic enzyme CYP2D6 metabolism. Use Caution/Monitor
amlodipine + carvedilol	4	carvedilol and amlodipine both increase anti-hypertensive channel blocking. Modify Therapy/Monitor Closely
amlodipine + nebivolol	4	nebivolol, amlodipine. Either increases effects of the other by pharmacodynamic synergism. Use Caution/Monitor. Both drugs lower blood pressure
amoxicillin + aspirin	4	amoxicillin, aspirin. Either increases levels of the other by plasma protein binding competition. Use Caution/Monitor
		amoxicillin, aspirin. Either increases levels of the other by decreasing renal clearance. Use Caution/Monitor
aspirin + dexamethasone	4	aspirin, dexamethasone. Either increases toxicity of the other by pharmacodynamic synergism. Use Caution/Monitor. Increased risk of GI ulceration
aspirin + diclofenac	4	aspirin and diclofenac both increase anticoagulation. Use Caution/Monitor
		aspirin and diclofenac both increase serum potassium. Use Caution/Monitor

aspirin + doxazosin	4	aspirin decreases effects of doxazosin by pharmacodynamic antagonism. Use Caution/Monitor. NSAIDs decrease prostaglandin synthesis
aspirin + ketorolac	4	aspirin and ketorolac both increase anticoagulation. Use Caution/Monitor
		aspirin and ketorolac both increase serum potassium. Use Caution/Monitor
aspirin + meloxicam	4	aspirin and meloxicam both increase anticoagulation. Use Caution/Monitor
		aspirin and meloxicam both increase serum potassium. Use Caution/Monitor
atorvastatin + telmisartan	4	telmisartan increases toxicity of atorvastatin by Other (see comment). Use Caution/Monitor. Comment: OATP1B1 inhibitors may increase risk of myopathy
atorvastatin + verapamil	4	verapamil will increase the level or effect of atorvastatin by affecting hepatic/intestinal enzyme CYP3A4 metabolism. Use Caution/Monitor
		verapamil will increase the level or effect of atorvastatin by P-glycoprotein (MDR1) efflux transporter. Use Caution/Monitor
bisoprolol + diclofenac	4	bisoprolol and diclofenac both increase serum potassium. Use Caution/Monitor
bisoprolol + ketorolac	4	bisoprolol and ketorolac both increase serum potassium. Use Caution/Monitor
bisoprolol + nifedipine	4	bisoprolol, nifedipine. Either decreases effects of the other by pharmacodynamic synergism. Use Caution/Monitor. Both drugs lower blood pressure
calcium carbonate + metoprolol	4	calcium carbonate decreases effects of metoprolol by unspecified interaction mechanism. Use Caution/Monitor
		calcium carbonate decreases levels of metoprolol by inhibition of GI absorption. Applies only to oral form of both agents. Use Caution/Monitor. Separate by 2 hours
candesartan + torsemide	4	candesartan increases and torsemide decreases serum potassium. Effect of interaction is not clear, use caution. Use Caution/Monitor
carvedilol + losartan	4	carvedilol, losartan. Mechanism: pharmacodynamic synergism. Use Caution/Monitor. Risk of fetal compromise if given during pregnancy
clopidogrel + ticagrelor	4	ticagrelor, clopidogrel. Either increases effects of the other by Other (see comment). Use Caution/Monitor. Comment: Increased risk of bleeding during concomitant use of medications that increase potential for bleeding
clopidogrel + warfarin	4	clopidogrel, warfarin. Either increases effects of the other by pharmacodynamic synergism. Modify Therapy/Monitor Closely. Drugs with antiplatelet properties may increase anticoagulation effect of warfarin
dapagliflozin + insulin	4	dapagliflozin, insulin aspart. Either increases effects of the other by pharmacodynamic synergism. Use Caution/Monitor. Antidiabetic agents are often used in combination; dosage adjustments may be required when initiating or discontinuing antidiabetic agents
		dapagliflozin, insulin detemir. Either increases effects of the other by pharmacodynamic synergism. Use Caution/Monitor. Antidiabetic agents are often used in combination; dosage adjustments may be required when initiating or discontinuing antidiabetic agents
digoxin + perindopril	4	perindopril increases levels of digoxin by unspecified interaction mechanism. Use Caution/Monitor
enalapril + potassium	4	enalapril increases levels of potassium chloride by decreasing elimination. Use Caution/Monitor. Risk of hyperkalemia
eplerenone + fosinopril	4	fosinopril, eplerenone. Mechanism: pharmacodynamic synergism. Use Caution/Monitor. Risk of hyperkalemia

eplerenone + losartan	4	losartan, eplerenone. Mechanism: pharmacodynamic synergism. Use Caution/Monitor. Risk of hyperkalemia
eplerenone + sacubitril/valsartan	4	sacubitril/valsartan, eplerenone. Mechanism: pharmacodynamic synergism. Use Caution/Monitor. Risk of hyperkalemia
formoterol + furosemide	4	formoterol and furosemide both decrease serum potassium. Use Caution/Monitor
ivabradine + metoprolol	4	ivabradine, metoprolol. Either increases effects of the other by pharmacodynamic synergism. Modify Therapy/Monitor Closely. Most patients receiving ivabradine will also be treated with a beta-blocker. The risk of bradycardia increases with coadministration of drugs that slow heart rate (eg, digoxin, amiodarone, beta-blockers). Monitor heart rate in patients taking ivabradine with other negative chronotropes
losartan + nebivolol	4	losartan and nebivolol both increase serum potassium. Use Caution/Monitor
losartan + sotalol	4	losartan and sotalol both increase serum potassium. Use Caution/Monitor
metoprolol + valsartan	4	metoprolol, valsartan. Mechanism: pharmacodynamic synergism. Use Caution/Monitor. Risk of fetal compromise if given during pregnancy
sacubitril/valsartan + sotalol	4	sacubitril/valsartan and sotalol both increase serum potassium. Use Caution/Monitor
sacubitril/valsartan + torsemide	4	sacubitril/valsartan increases and torsemide decreases serum potassium. Effect of interaction is not clear, use caution. Use Caution/Monitor
sotalol + telmisartan	4	sotalol, telmisartan. Mechanism: pharmacodynamic synergism. Use Caution/Monitor. Risk of fetal compromise if given during pregnancy
amiodarone + carvedilol	3	amiodarone will increase the level or effect of carvedilol by affecting hepatic enzyme CYP2C9/10 metabolism. Use Caution/Monitor
		amiodarone will increase the level or effect of carvedilol by affecting hepatic enzyme CYP2D6 metabolism. Use Caution/Monitor. Monitor for signs of bradycardia or heart block when amiodarone and a beta adrenergic blocker are coadministered. Amiodarone should be used with caution in patients receiving a beta adrenergic blocker, particularly if there is suspicion of underlying dysfunction of the sinus node, such as bradycardia or sick sinus syndrome, or if there is partial AV block
		amiodarone, carvedilol. Mechanism: pharmacodynamic synergism. Use Caution/Monitor. Risk of cardiotoxicity with bradycardia
amiodarone + dabigatran	3	amiodarone will increase the level or effect of dabigatran by P-glycoprotein (MDR1) efflux transporter. Use Caution/Monitor. Atrial fibrillation: Avoid coadministering dabigatran with P-gp inhibitors if CrCl <30 mL/min. DVT/PE treatment: Avoid coadministering dabigatran with P-gp inhibitors if CrCl <50 mL/min
aspirin + dabigatran	3	dabigatran and aspirin both increase anticoagulation. Modify Therapy/Monitor Closely. Both drugs have the potential to cause bleeding. The need for simultaneous use of low-dose aspirin (<100 mg/day) with anticoagulants are common for patients with cardiovascular disease, but may result in increased bleeding; monitor closely. Promptly evaluate any signs or symptoms of blood loss if treated concomitantly with low-dose aspirin. Avoid coadministration with chronic use of higher dose aspirin
aspirin + formoterol	3	aspirin increases and formoterol decreases serum potassium. Effect of interaction is not clear, use caution. Use Caution/Monitor

aspirin + olmesartan	3	aspirin decreases effects of olmesartan by pharmacodynamic antagonism. Modify Therapy/Monitor Closely. NSAIDs decrease synthesis of vasodilating renal prostaglandins, and thus affect fluid homeostasis and may diminish antihypertensive effect
bisoprolol + sodium bicarbonate	3	sodium bicarbonate decreases levels of bisoprolol by inhibition of GI absorption. Applies only to oral form of both agents. Use Caution/Monitor. Separate by 2 hours
budesonide + dexamethasone	3	budesonide will decrease the level or effect of dexamethasone by affecting hepatic/intestinal enzyme CYP3A4 metabolism. Use Caution/Monitor
captopril + chlorthalidone	3	captopril, chlorthalidone. Either increases toxicity of the other by pharmacodynamic synergism. Use Caution/ Monitor. Both drugs lower blood pressure. Increased risk of nephrotoxicity. Monitor blood pressure and renal function
captopril + furosemide	3	captopril, furosemide. Mechanism: pharmacodynamic synergism. Use Caution/Monitor. Risk of acute hypotension, renal insufficiency
carvedilol + torsemide	3	carvedilol increases and torsemide decreases serum potassium. Effect of interaction is not clear, use caution. Use Caution/Monitor
clopidogrel + felodipine	3	felodipine decreases effects of clopidogrel by decreasing metabolism. Use Caution/Monitor. Cytochrome P450 2C19 inhibitors decrease the conversion of clopidogrel to its active form
dexamethasone + heparin	3	dexamethasone, heparin. Other (see comment). Use Caution/Monitor. Comment: Corticosteroids may decrease anticoagulant effects by increasing blood coagulability; conversely, they may impair vascular integrity, thus increasing bleeding risk. Monitor INR closely
dexamethasone + verapamil	3	dexamethasone will decrease the level or effect of verapamil by affecting hepatic/intestinal enzyme CYP3A4 metabolism. Use Caution/Monitor
digoxin + losartan	3	losartan and digoxin both increase serum potassium. Use Caution/Monitor
digoxin + metformin	3	digoxin, metformin. Either increases levels of the other by basic (cationic) drug competition for renal tubular clearance. Use Caution/Monitor. Measure serum digoxin concentrations before initiating metformin. Monitor patients who take both metformin and digoxin for possible digoxin toxicity and lactic acidosis. Reduce the digoxin and/or metformin dose as necessary
empagliflozin + insulin	3	empagliflozin, insulin aspart. Either increases effects of the other by pharmacodynamic synergism. Modify Therapy/Monitor Closely. Consider a lower dose of insulin or insulin secretagogue to avoid hypoglycemia when coadministered with SGLT2 inhibitors
enoxaparin + fosinopril	3	enoxaparin increases toxicity of fosinopril by Other (see comment). Use Caution/Monitor. Comment: Low molecular weight heparins may suppress adrenal aldosterone secretion, which can potentially cause hyperkalemia
enoxaparin + lisinopril	3	enoxaparin increases toxicity of lisinopril by Other (see comment). Use Caution/Monitor. Comment: Low molecular weight heparins may suppress adrenal aldosterone secretion, which can potentially cause hyperkalemia
enoxaparin + rivaroxaban	3	rivaroxaban, enoxaparin. Either increases effects of the other by anticoagulation. Use Caution/Monitor. Avoid concurrent use of rivaroxaban with other anticoagulants due to increased bleeding risk other than during therapeutic transition periods where patients should be observed closely. Monitor for signs/symptoms of blood loss
eplerenone + losartan	3	losartan, eplerenone. Mechanism: pharmacodynamic synergism. Use Caution/Monitor. Risk of hyperkalemia

ferrous sulfate + omeprazole	3	omeprazole will decrease the level or effect of ferrous sulfate by increasing gastric pH. Applies only to oral form of both agents. Use Caution/Monitor
formoterol + spironolactone	3	spironolactone increases and formoterol decreases serum potassium. Effect of interaction is not clear, use caution. Modify Therapy/Monitor Closely
fosinopril + metformin	3	fosinopril increases toxicity of metformin by unspecified interaction mechanism. Use Caution/Monitor. Increases risk for hypoglycemia and lactic acidosis
furosemide + perindopril	3	perindopril, furosemide. Mechanism: pharmacodynamic synergism. Use Caution/Monitor. Risk of acute hypotension, renal insufficiency
heparin + rivaroxaban	3	rivaroxaban, heparin. Either increases effects of the other by anticoagulation. Use Caution/Monitor. Avoid concurrent use of rivaroxaban with other anticoagulants due to increased bleeding risk other than during therapeutic transition periods where patients should be observed closely. Monitor for signs/symptoms of blood loss
heparin + spironolactone	3	spironolactone, heparin. Either increases toxicity of the other by serum potassium. Use Caution/Monitor. Both drugs may increase serum potassium levels
heparin + ticagrelor	3	ticagrelor, heparin. Either increases effects of the other by anticoagulation. Use Caution/Monitor. Increased risk of bleeding during concomitant use of medications that increase potential for bleeding
ibuprofen + losartan	3	ibuprofen decreases effects of losartan by pharmacodynamic antagonism. Modify Therapy/Monitor Closely. NSAIDs decrease synthesis of vasodilating renal prostaglandins, and thus affect fluid homeostasis and may diminish antihypertensive effect
indomethacin + losartan	3	indomethacin decreases effects of losartan by pharmacodynamic antagonism. Modify Therapy/Monitor Closely. NSAIDs decrease synthesis of vasodilating renal prostaglandins, and thus affect fluid homeostasis and may diminish antihypertensive effect
ketorolac + lisinopril	3	ketorolac, lisinopril. pharmacodynamic antagonism. Avoid or Use Alternate Drug. Coadministration may result in a significant decrease in renal function. NSAIDs may diminish the antihypertensive effect of ACE inhibitors. The mechanism of these interactions is likely related to the ability of NSAIDs to reduce the synthesis of vasodilating renal prostaglandins
lisinopril + potassium	3	lisinopril increases levels of potassium chloride by decreasing elimination. Use Caution/Monitor. Risk of hyperkalemia
losartan + potassium	3	losartan and potassium chloride both increase serum potassium. Use Caution/Monitor
metoprolol + potassium	3	metoprolol and potassium acid phosphate both increase serum potassium. Modify Therapy/Monitor Closely
sacubitril/valsartan + spironolactone	3	sacubitril/valsartan and spironolactone both increase serum potassium. Modify Therapy/Monitor Closely
sotalol + torsemide	3	sotalol increases and torsemide decreases serum potassium. Effect of interaction is not clear, use caution. Use Caution/Monitor
torsemide + valsartan	3	valsartan increases and torsemide decreases serum potassium. Effect of interaction is not clear, use caution. Use Caution/Monitor
aceclofenac + aspirin	2	aceclofenac and aspirin both increase anticoagulation. Use Caution/Monitor
		aceclofenac and aspirin both increase serum potassium. Use Caution/Monitor
aceclofenac + bisoprolol	2	aceclofenac decreases effects of bisoprolol by pharmacodynamic antagonism. Use Caution/Monitor. Long term (>1 wk) NSAID use. NSAIDs decrease prostaglandin synthesis

amiodarone + budesonide	2	amiodarone will increase the level or effect of budesonide by P-glycoprotein (MDR1) efflux transporter. Use Caution/Monitor
amiodarone + hydrochlorothiazide	2	amiodarone will increase the level or effect of hydrochlorothiazide by basic (cationic) drug competition for renal tubular clearance. Use Caution/Monitor
amitriptyline + tramadol	2	amitriptyline and tramadol both increase serotonin levels. Modify Therapy/Monitor Closely
ampicillin + spironolactone	2	ampicillin increases effects of spironolactone by unspecified interaction mechanism. Use Caution/Monitor. Hyperkalemia
aspirin + betaxolol	2	aspirin decreases effects of betaxolol by pharmacodynamic antagonism. Use Caution/Monitor. Long term (>1 wk) NSAID use. NSAIDs decrease prostaglandin synthesis
aspirin + ibuprofen	2	aspirin and ibuprofen both increase anticoagulation. Use Caution/ Monitor
		aspirin and ibuprofen both increase serum potassium. Use Caution/ Monitor
aspirin + ketoprofen	2	aspirin and ketoprofen both increase serum potassium. Use Caution/Monitor
		aspirin and ketoprofen both increase anticoagulation. Use Caution/Monitor
aspirin + nebivolol	2	aspirin decreases effects of nebivolol by pharmacodynamic antagonism. Use Caution/Monitor. Long term (>1 wk) NSAID use. NSAIDs decrease prostaglandin synthesis
aspirin + prasugrel	2	aspirin, prasugrel. Either increases toxicity of the other by pharmacodynamic synergism. Use Caution/Monitor. The need for simultaneous use of low-dose aspirin and anticoagulant or antiplatelet agents are common for patients with cardiovascular disease; monitor closely
aspirin + ramipril	2	aspirin, ramipril. pharmacodynamic antagonism. Avoid or Use Alternate Drug. Coadministration may result in a significant decrease in renal function. NSAIDs may diminish the antihypertensive effect of ACE inhibitors. The mechanism of these interactions is likely related to the ability of NSAIDs to reduce the synthesis of vasodilating renal prostaglandins
aspirin + timolol	2	timolol and aspirin both increase serum potassium. Use Caution/ Monitor
atorvastatin + fluconazole	2	fluconazole will increase the level or effect of atorvastatin by affecting hepatic/intestinal enzyme CYP3A4 metabolism. Use Caution/Monitor
azilsartan + meloxicam	2	meloxicam, azilsartan. Either increases toxicity of the other by Other (see comment). Use Caution/Monitor. Comment: May result in renal function deterioration, particularly in elderly or volume depleted individuals
		meloxicam decreases effects of azilsartan by pharmacodynamic antagonism. Modify Therapy/Monitor Closely. NSAIDs decrease synthesis of vasodilating renal prostaglandins, and thus affect fluid homeostasis and may diminish antihypertensive effect
betaxolol + olmesartan	2	betaxolol, olmesartan. Mechanism: pharmacodynamic synergism. Use Caution/Monitor. Risk of fetal compromise if given during pregnancy
bisoprolol + calcium carbonate	2	calcium carbonate decreases effects of bisoprolol by unspecified interaction mechanism. Use Caution/Monitor
		calcium carbonate decreases levels of bisoprolol by inhibition of GI absorption. Applies only to oral form of both agents. Use Caution/Monitor. Separate by 2 hours
bisoprolol + chlorthalidone	2	bisoprolol increases and chlorthalidone decreases serum potassium. Effect of interaction is not clear, use caution. Use Caution/Monitor
bisoprolol + ibuprofen	2	bisoprolol and ibuprofen both increase serum potassium. Use Caution/Monitor
bisoprolol + ketoprofen	2	bisoprolol and ketoprofen both increase serum potassium. Use Caution/Monitor
bisoprolol + lornoxicam	2	bisoprolol and lornoxicam both increase serum potassium. Use Caution/Monitor

calcium carbonate + nifedipine	2	calcium carbonate decreases effects of nifedipine by pharmacodynamic antagonism. Use Caution/Monitor
canagliflozin + glimepiride	2	glimepiride, canagliflozin. Either increases effects of the other by pharmacodynamic synergism. Modify Therapy/Monitor Closely. Consider a lower dose of insulin or insulin secretagogue to avoid hypoglycemia when coadministered with canagliflozin
canagliflozin + lisinopril	2	lisinopril and canagliflozin both increase serum potassium. Use Caution/Monitor
canagliflozin + spironolactone	2	spironolactone and canagliflozin both increase serum potassium. Use Caution/Monitor
captopril + eplerenone	2	captopril, eplerenone. Either increases toxicity of the other by Mechanism: pharmacodynamic synergism. Use Caution/Monitor. Risk of hyperkalemia. Monitor potassium
captopril + potassium	2	captopril increases levels of potassium chloride by decreasing elimination. Use Caution/Monitor. Risk of hyperkalemia
		potassium chloride increases toxicity of captopril by Mechanism: unspecified interaction mechanism. Modify Therapy/Monitor Closely. Both drugs increase potassium. Monitor potassium
carbamazepine + enalapril	2	enalapril increases levels of carbamazepine by decreasing metabolism. Use Caution/Monitor
carbamazepine + omeprazole	2	carbamazepine will decrease the level or effect of omeprazole by affecting hepatic enzyme CYP2C19 metabolism. Avoid or Use Alternate Drug
		carbamazepine will decrease the level or effect of omeprazole by affecting hepatic/intestinal enzyme CYP3A4 metabolism. Use Caution/Monitor
carvedilol + spironolactone	2	carvedilol and spironolactone both increase serum potassium. Modify Therapy/Monitor Closely
cefotaxime + warfarin	2	cefotaxime increases effects of warfarin by unspecified interaction mechanism. Use Caution/Monitor
celecoxib + meloxicam	2	celecoxib and meloxicam both increase anticoagulation. Use Caution/Monitor
		celecoxib and meloxicam both increase serum potassium. Use Caution/Monitor
chlorthalidone + metoprolol	2	metoprolol increases and chlorthalidone decreases serum potassium. Effect of interaction is not clear, use caution. Use Caution/Monitor
clopidogrel + dexamethasone	2	dexamethasone will increase the level or effect of clopidogrel by affecting hepatic/intestinal enzyme CYP3A4 metabolism. Use Caution/Monitor. CYP3A4 inducers may increase the metabolism of clopidogrel to its active metabolite. Monitor patients for potential increase in antiplatelet effects when CYP3A4 inducers are used in combination with clopidogrel
dabigatran + clopidogrel	2	dabigatran, clopidogrel. Either increases effects of the other by pharmacodynamic synergism. Use Caution/Monitor. Both drugs have the potential to cause bleeding. Concomitant use may increase risk of bleeding
dabigatran + rivaroxaban	2	rivaroxaban, dabigatran. Either increases effects of the other by anticoagulation. Use Caution/Monitor. Avoid concurrent use of rivaroxaban with other anticoagulants due to increased bleeding risk other than during therapeutic transition periods where patients should be observed closely. Monitor for signs/symptoms of blood loss
dexamethasone + metronidazole	2	metronidazole will increase the level or effect of dexamethasone by affecting hepatic/intestinal enzyme CYP3A4 metabolism. Use Caution/Monitor

diclofenac + enalapril	2	enalapril, diclofenac. Either increases toxicity of the other by Other (see comment). Use Caution/Monitor. Comment: May result in renal function deterioration, particularly in elderly or volume depleted individuals
diclofenac + ketoprofen	2	diclofenac and ketoprofen both increase anticoagulation. Use Caution/Monitor
		diclofenac and ketoprofen both increase serum potassium. Use Caution/Monitor
		diclofenac will increase the level or effect of ketoprofen by acidic (anionic) drug competition for renal tubular clearance. Minor/Significance Unknown
diclofenac + ketorolac	2	diclofenac, ketorolac. Either increases toxicity of the other by pharmacodynamic synergism. Contraindicated
		diclofenac and ketorolac both increase anticoagulation. Use Caution/Monitor
		diclofenac and ketorolac both increase serum potassium. Use Caution/Monitor
diclofenac + lornoxicam	2	diclofenac and lornoxicam both increase anticoagulation. Use Caution/Monitor
		diclofenac and lornoxicam both increase serum potassium. Use Caution/Monitor
diclofenac + meloxicam	2	diclofenac and meloxicam both increase anticoagulation. Use Caution/Monitor
		diclofenac and meloxicam both increase serum potassium. Use Caution/Monitor
diclofenac + sotalol	2	diclofenac decreases effects of sotalol by pharmacodynamic antagonism. Use Caution/Monitor. Long term (>1 wk) NSAID use. NSAIDs decrease prostaglandin synthesis
digoxin + hydrochlorothiazide	2	digoxin will increase the level or effect of hydrochlorothiazide by basic (cationic) drug competition for renal tubular clearance. Use Caution/Monitor
digoxin + hydrochlorothiazide	2	digoxin increases and hydrochlorothiazide decreases serum potassium. Effect of interaction is not clear, use caution. Use Caution/Monitor
digoxin + indapamide	2	digoxin increases and indapamide decreases serum potassium. Effect of interaction is not clear, use caution. Use Caution/Monitor
digoxin + lisinopril	2	lisinopril increases levels of digoxin by unspecified interaction mechanism. Use Caution/Monitor
doxazosin + metoprolol	2	doxazosin and metoprolol both increase anti-hypertensive channel blocking. Modify Therapy/Monitor Closely
doxazosin + nifedipine	2	doxazosin and nifedipine both increase anti-hypertensive channel blocking. Use Caution/Monitor
empagliflozin + glimepiride	2	empagliflozin, glimepiride. Either increases effects of the other by pharmacodynamic synergism. Modify Therapy/Monitor Closely. Consider a lower dose of insulin or insulin secretagogue to avoid hypoglycemia when coadministered with SGLT2 inhibitors
enalapril + heparin	2	heparin increases toxicity of enalapril by Other (see comment). Use Caution/Monitor. Comment: Low molecular weight heparins may suppress adrenal aldosterone secretion, which can potentially cause hyperkalemia
enoxaparin + ketorolac	2	enoxaparin and ketorolac both increase anticoagulation. Modify Therapy/Monitor Closely
felodipine + metformin	2	felodipine decreases effects of metformin by pharmacodynamic antagonism. Use Caution/Monitor. Patient should be closely observed for loss of blood glucose control; when drugs are withdrawn from a patient receiving metformin, patient should be observed closely for hypoglycemia

felodipine + nitroglycerin	2	felodipine, nitroglycerin sublingual. Either increases toxicity of the other by additive vasodilation. Modify Therapy/Monitor Closely. Marked orthostatic hypotension reported with concomitant use
fluconazole + omeprazole	2	fluconazole will increase the level or effect of omeprazole by affecting hepatic enzyme CYP2C19 metabolism. Use Caution/Monitor
fluconazole + sulfamethoxazole	2	fluconazole will increase the level or effect of sulfamethoxazole by affecting hepatic enzyme CYP2C9/10 metabolism. Minor/Significance Unknown
fluconazole + trimethoprim	2	fluconazole and trimethoprim both increase QTc interval. Modify Therapy/Monitor Closely
furosemide + sacubitril/valsartan	2	sacubitril/valsartan increases and furosemide decreases serum potassium. Effect of interaction is not clear, use caution. Use Caution/Monitor
glimepiride + lisinopril	2	lisinopril increases effects of glimepiride by pharmacodynamic synergism. Use Caution/Monitor
heparin + losartan	2	heparin increases toxicity of losartan by Other (see comment). Use Caution/Monitor. Comment: Low molecular weight heparins may suppress adrenal aldosterone secretion, which can potentially cause hyperkalemia
heparin + prednisolone	2	prednisolone, heparin. Other (see comment). Use Caution/Monitor. Comment: Corticosteroids may decrease anticoagulant effects by increasing blood coagulability; conversely, they may impair vascular integrity, thus increasing bleeding risk. Monitor INR closely
hydrochlorothiazide + metoprolol	2	hydrochlorothiazide, metoprolol. Either increases toxicity of the other by Other (see comment). Modify Therapy/Monitor Closely. Comment: May cause idiosyncratic reaction, resulting in acute transient myopia and acute angle-closure glaucoma, which can lead to permanent vision loss
hydrochlorothiazide + telmisartan	2	telmisartan increases and hydrochlorothiazide decreases serum potassium. Effect of interaction is not clear, use caution. Use Caution/Monitor
hydrochlorothiazide + torsemide	2	torsemide and hydrochlorothiazide both decrease serum potassium. Use Caution/Monitor
indapamide + potassium	2	potassium chloride increases and indapamide decreases serum potassium. Effect of interaction is not clear, use caution. Modify Therapy/Monitor Closely
indapamide + sotalol	2	sotalol increases and indapamide decreases serum potassium. Effect of interaction is not clear, use caution. Use Caution/Monitor
insulin + levofloxacin	2	levofloxacin increases effects of insulin detemir by pharmacodynamic synergism. Use Caution/Monitor. Quinolone antibiotic administration may result in hyper- or hypoglycemia. Gatifloxacin is most likely to produce dysglycemia; moxifloxacin is least likely
ivabradine + bisoprolol	2	ivabradine, bisoprolol. Either increases effects of the other by pharmacodynamic synergism. Modify Therapy/Monitor Closely. Most patients receiving ivabradine will also be treated with a beta-blocker. The risk of bradycardia increases with coadministration of drugs that slow heart rate (eg, digoxin, amiodarone, beta-blockers). Monitor heart rate in patients taking ivabradine with other negative chronotropes
ketoprofen + meloxicam	2	ketoprofen and meloxicam both increase anticoagulation. Use Caution/Monitor
		ketoprofen and meloxicam both increase serum potassium. Use Caution/Monitor
ketoprofen + sotalol	2	ketoprofen decreases effects of sotalol by pharmacodynamic antagonism. Use Caution/Monitor. Long term (>1 wk) NSAID use. NSAIDs decrease prostaglandin synthesis

ketorolac + meloxicam	2	ketorolac and meloxicam both increase anticoagulation. Use Caution/Monitor
		ketorolac and meloxicam both increase serum potassium. Use Caution/Monitor
ketorolac + nebivolol	2	ketorolac decreases effects of nebivolol by pharmacodynamic antagonism. Use Caution/Monitor. Long term (>1 wk) NSAID use. NSAIDs decrease prostaglandin synthesis
levothyroxine + metformin	2	levothyroxine decreases effects of metformin by pharmacodynamic antagonism. Use Caution/Monitor. Patient should be closely observed for loss of blood glucose control; when drugs are withdrawn from a patient receiving metformin, patient should be observed closely for hypoglycemia
meloxicam + sotalol	2	meloxicam decreases effects of sotalol by pharmacodynamic antagonism. Use Caution/Monitor. Long term (>1 wk) NSAID use. NSAIDs decrease prostaglandin synthesis
metoprolol + ranolazine	2	ranolazine will increase the level or effect of metoprolol by affecting hepatic enzyme CYP2D6 metabolism. Use Caution/Monitor
metoprolol + telmisartan	2	metoprolol, telmisartan. Mechanism: pharmacodynamic synergism. Use Caution/Monitor. Risk of fetal compromise if given during pregnancy
nifedipine + nitroglycerin	2	nifedipine, nitroglycerin sublingual. Either increases toxicity of the other by additive vasodilation. Modify Therapy/ Monitor Closely. Marked orthostatic hypotension reported with concomitant use
perindopril + potassium	2	perindopril increases levels of potassium chloride by decreasing elimination. Use Caution/Monitor. Risk of hyperkalemia
phenylephrine + xylometazoline	2	phenylephrine and xylometazoline both decrease sedation. Use Caution/Monitor
		phenylephrine and xylometazoline both increase sympathetic (adrenergic) effects, including increased blood pressure and heart rate. Use Caution/Monitor
sotalol + valsartan	2	sotalol, valsartan. Mechanism: pharmacodynamic synergism. Use Caution/Monitor. Risk of fetal compromise if given during pregnancy
spironolactone + valsartan	2	valsartan and spironolactone both increase serum potassium. Modify Therapy/Monitor Closely
telmisartan + torsemide	2	telmisartan increases and torsemide decreases serum potassium. Effect of interaction is not clear, use caution. Use Caution/Monitor
aceclofenac + indapamide	1	aceclofenac increases and indapamide decreases serum potassium. Effect of interaction is not clear, use caution. Use Caution/Monitor
allopurinol + calcium carbonate	1	calcium carbonate decreases levels of allopurinol by inhibition of GI absorption. Applies only to oral form of both agents. Use Caution/Monitor. Separate by 2 hours
amikacin + digoxin	1	amikacin will increase the level or effect of digoxin by altering intestinal flora. Applies only to oral form of both agents. Use Caution/Monitor
amikacin + vancomycin	1	amikacin and vancomycin both increase nephrotoxicity and/or ototoxicity. Use Caution/Monitor
amiodarone + atenolol	1	amiodarone, atenolol. Mechanism: pharmacodynamic synergism. Use Caution/Monitor. Risk of cardiotoxicity with bradycardia
amiodarone + metronidazole	1	metronidazole will increase the level or effect of amiodarone by affecting hepatic/intestinal enzyme CYP3A4 metabolism. Use Caution/Monitor
amitriptyline + atorvastatin	1	atorvastatin will increase the level or effect of amitriptyline by P-glycoprotein (MDR1) efflux transporter. Use Caution/Monitor

amitriptyline + carbamazepine	1	carbamazepine will decrease the level or effect of amitriptyline by affecting hepatic/intestinal enzyme CYP3A4 metabolism. Use Caution/Monitor
amitriptyline + gabapentin	1	gabapentin, amitriptyline. Either increases effects of the other by pharmacodynamic synergism. Modify Therapy/Monitor Closely. Coadministration of CNS depressants can result in serious, life-threatening, and fatal respiratory depression. Use lowest dose possible and monitor for respiratory depression and sedation
amlodipine + betaxolol	1	betaxolol, amlodipine. Either increases effects of the other by pharmacodynamic synergism. Use Caution/Monitor. Both drugs lower blood pressure
amlodipine + clarithromycin	1	clarithromycin will increase the level or effect of amlodipine by affecting hepatic/intestinal enzyme CYP3A4 metabolism. Use Caution/Monitor. Increased effect of calcium channel blockers may lead to hypotension, edema, decreased HR, and acute kidney injury due to reduced renal blood flow
amlodipine + doxazosin	1	doxazosin and amlodipine both increase anti-hypertensive channel blocking. Use Caution/Monitor
amlodipine + magnesium supplement	1	magnesium supplement, amlodipine. Either increases toxicity of the other by pharmacodynamic synergism. Use Caution/Monitor. Calcium channel blockers may increase toxic effects of magnesium; magnesium may increase hypotensive effects of calcium channel blockers
ampicillin + aspirin	1	ampicillin, aspirin. Either increases levels of the other by plasma protein binding competition. Use Caution/Monitor
ampicillin + omeprazole	1	omeprazole will decrease the level or effect of ampicillin by increasing gastric pH. Applies only to oral form of both agents. Use Caution/Monitor
aspirin + escitalopram	1	escitalopram, aspirin. Either increases toxicity of the other by pharmacodynamic synergism. Use Caution/Monitor. Increased risk of upper GI bleeding. SSRIs inhib. serotonin uptake by platelets
aspirin + fondaparinux	1	fondaparinux and aspirin both increase anticoagulation. Modify Therapy/Monitor Closely
atenolol + chlorthalidone	1	atenolol increases and chlorthalidone decreases serum potassium. Effect of interaction is not clear, use caution. Use Caution/Monitor
atorvastatin + metronidazole	1	metronidazole will increase the level or effect of atorvastatin by affecting hepatic/intestinal enzyme CYP3A4 metabolism. Use Caution/Monitor
azithromycin + atorvastatin	1	azithromycin will increase the level or effect of atorvastatin by affecting hepatic/intestinal enzyme CYP3A4 metabolism. Use Caution/Monitor. If this combination is used, closely monitor for evidence of atorvastatin toxicity (eg, muscle aches or pains, renal dysfunction)
azithromycin + sodium bicarbonate	1	sodium bicarbonate decreases levels of azithromycin by inhibition of GI absorption. Applies only to oral form of both agents. Use Caution/Monitor. Separate by 2 hours
azithromycin + warfarin	1	azithromycin increases toxicity of warfarin by anticoagulation. Use Caution/Monitor. Postmarketing reports have suggested that concomitant administration of azithromycin may potentiate effects of oral warfarin but the interaction does not appear to alter prothrombin time
bisoprolol + doxazosin	1	doxazosin and bisoprolol both increase anti-hypertensive channel blocking. Modify Therapy/ Monitor Closely
bisoprolol + insulin	1	bisoprolol, insulin degludec. Other (see comment). Modify Therapy/Monitor Closely. Comment: Beta-blockers may either increase or decrease the blood glucose lowering effect of insulin; beta-blockers can prolong hypoglycemia (interference with glycogenolysis) or cause hyperglycemia (insulin secretion inhibited)

bisoprolol + nebivolol	1	bisoprolol and nebivolol both increase anti-hypertensive channel blocking. Avoid or Use Alternate Drug
bisoprolol + timolol	1	bisoprolol and timolol both increase serum potassium. Use Caution/ Monitor
bisoprolol + verapamil	1	bisoprolol and verapamil both increase anti-hypertensive channel blocking. Modify Therapy/Monitor Closely
budesonide + clarithromycin	1	clarithromycin will increase the level or effect of budesonide by P-glycoprotein (MDR1) efflux transporter. Use Caution/ Monitor
budesonide + clopidogrel	1	budesonide will increase the level or effect of clopidogrel by affecting hepatic/intestinal enzyme CYP3A4 metabolism. Use Caution/Monitor. CYP3A4 inducers may increase the metabolism of clopidogrel to its active metabolite. Monitor patients for potential increase in antiplatelet effects when CYP3A4 inducers are used in combination with clopidogrel
budesonide + enoxaparin	1	budesonide, enoxaparin. Other (see comment). Use Caution/Monitor. Comment: Corticosteroids may decrease anticoagulant effects by increasing blood coagulability; conversely, they may impair vascular integrity, thus increasing bleeding risk. Monitor INR closely
budesonide + esomeprazole	1	esomeprazole decreases effects of budesonide by increasing gastric pH. Applies only to oral form of both agents. Modify Therapy/Monitor Closely. Enteric-coated budesonide dissolves at pH >5.5. Also, dissolution of extended-release budesonide tablets is pH dependent. Coadministration with drugs that increase gastric pH may cause these budesonide products to prematurely dissolve, and possibly affect release properties and absorption of the drug in the duodenum
budesonide + heparin	1	budesonide, heparin. Other (see comment). Use Caution/Monitor. Comment: Corticosteroids may decrease anticoagulant effects by increasing blood coagulability; conversely, they may impair vascular integrity, thus increasing bleeding risk. Monitor INR closely
budesonide + ketorolac	1	ketorolac, budesonide. Either increases toxicity of the other by pharmacodynamic synergism. Use Caution/Monitor. Increased risk of GI ulceration
budesonide + theophylline	1	budesonide will decrease the level or effect of theophylline by affecting hepatic/intestinal enzyme CYP3A4 metabolism. Use Caution/Monitor
calcium carbonate + captopril	1	calcium carbonate decreases effects of captopril by unspecified interaction mechanism. Use Caution/Monitor. Calcium carbonate may decrease absorption of captopril
calcium carbonate + celecoxib	1	calcium carbonate decreases levels of celecoxib by inhibition of GI absorption. Applies only to oral form of both agents. Use Caution/Monitor. Separate by 2 hours
calcium carbonate + levothyroxine	1	calcium carbonate decreases levels of levothyroxine by inhibition of GI absorption. Applies only to oral form of both agents. Use Caution/Monitor. Separate administration by 4 hours
calcium carbonate + ramipril	1	calcium carbonate decreases effects of ramipril by unspecified interaction mechanism. Use Caution/Monitor
calcium carbonate + rosuvastatin	1	calcium carbonate decreases levels of rosuvastatin by inhibition of GI absorption. Applies only to oral form of both agents. Use Caution/Monitor. Separate by 2 hours
calcium carbonate + vitamin D	1	vitamin D, calcium carbonate. Other (see comment). Use Caution/Monitor. Comment: The concurrent use of vitamin D with calcium salts is generally beneficial; in some patients this combination may result in hypercalcemia
calcium gluconate + levothyroxine	1	calcium gluconate decreases levels of levothyroxine by inhibition of GI absorption. Applies only to oral form of both agents. Use Caution/Monitor. Separate administration by 4 hours

candesartan + eplerenone	1	candesartan, eplerenone. Mechanism: pharmacodynamic synergism. Use Caution/Monitor. Risk of hyperkalemia
candesartan + indapamide	1	candesartan increases and indapamide decreases serum potassium. Effect of interaction is not clear, use caution. Use Caution/Monitor
candesartan + insulin	1	candesartan increases effects of insulin detemir by unspecified interaction mechanism. Use Caution/Monitor. Concomitant use of insulin and ARBs may require insulin dosage adjustment and increased glucose monitoring
candesartan + spironolactone	1	candesartan and spironolactone both increase serum potassium. Modify Therapy/Monitor Closely
captopril + diclofenac	1	captopril, diclofenac. Either increases toxicity of the other by Other (see comment). Use Caution/Monitor. Comment: May result in renal function deterioration, particularly in elderly or volume depleted individuals
captopril + isosorbide dinitrate	1	isosorbide dinitrate, captopril. Either increases effects of the other by pharmacodynamic synergism. Use Caution/Monitor. Both drugs lower blood pressure. Monitor blood pressure
captopril + ketoprofen	1	captopril, ketoprofen. Either increases toxicity of the other by Other (see comment). Use Caution/Monitor. Comment: May result in renal function deterioration, particularly in elderly or volume depleted individuals
captopril + ketorolac	1	captopril, ketorolac. Either increases toxicity of the other by Other (see comment). Use Caution/Monitor. Comment: May result in renal function deterioration, particularly in elderly or volume depleted individuals
captopril + meloxicam	1	captopril, meloxicam. Either increases toxicity of the other by Other (see comment). Use Caution/Monitor. Comment: May result in renal function deterioration, particularly in elderly or volume depleted individuals
captopril + metformin	1	captopril increases toxicity of metformin by unspecified interaction mechanism. Use Caution/ Monitor. Increases risk for hypoglycemia and lactic acidosis
captopril + pentoxifylline	1	pentoxifylline, captopril. Either increases effects of the other by pharmacodynamic synergism. Use Caution/Monitor. Both drugs lower blood pressure. Monitor blood pressure
carbamazepine + heparin	1	carbamazepine decreases levels of heparin by increasing metabolism. Use Caution/Monitor
carbamazepine + linezolid	1	carbamazepine decreases levels of linezolid by increasing metabolism. Use Caution/Monitor
carbamazepine + rivaroxaban	1	carbamazepine decreases levels of rivaroxaban by affecting hepatic/intestinal enzyme CYP3A4 metabolism. Use Caution/Monitor. Avoid concomitant use of rivaroxaban with drugs that are combined P-gp and strong CYP3A4 inducers. Consider increasing the rivaroxaban dose if these drugs must be coadministered
carvedilol + hydrochlorothiazide	1	carvedilol increases and hydrochlorothiazide decreases serum potassium. Effect of interaction is not clear, use caution. Use Caution/ Monitor
carvedilol + indapamide	1	carvedilol increases and indapamide decreases serum potassium. Effect of interaction is not clear, use caution. Use Caution/Monitor
carvedilol + nifedipine	1	carvedilol and nifedipine both increase anti-hypertensive channel blocking. Modify Therapy/Monitor Closely
celecoxib + heparin	1	heparin and celecoxib both increase anticoagulation. Modify Therapy/Monitor Closely

celecoxib + indapamide	1	celecoxib increases and indapamide decreases serum potassium. Effect of interaction is not clear, use caution. Use Caution/Monitor
celecoxib + lisinopril	1	lisinopril, celecoxib. Either increases toxicity of the other by Other (see comment). Use Caution/Monitor. Comment: May result in renal function deterioration, particularly in elderly or volume depleted individuals
celecoxib + rivaroxaban	1	rivaroxaban, celecoxib. Other (see comment). Use Caution/Monitor. Comment: NSAIDs are known to increase bleeding. Bleeding risk may be increased when NSAIDs are used concomitantly with rivaroxaban. Monitor for signs/symptoms of blood loss
chlorthalidone + meloxicam	1	chlorthalidone will increase the level or effect of meloxicam by acidic (anionic) drug competition for renal tubular clearance. Minor/Significance Unknown
ciprofloxacin + diclofenac	1	diclofenac, ciprofloxacin. Other (see comment). Modify Therapy/Monitor Closely. Comment: Mechanism: unknown. Increased risk of CNS stimulation and seizures with high doses of fluoroquinolones
ciprofloxacin + omeprazole	1	omeprazole will decrease the level or effect of ciprofloxacin by unknown mechanism. Use Caution/Monitor. Absorption of the ciprofloxacin ER tablet was slightly diminished (20%) when coadministered with omeprazole
clarithromycin + dexamethasone	1	clarithromycin will increase the level or effect of dexamethasone by P-glycoprotein (MDR1) efflux transporter. Use Caution/Monitor
clopidogrel + diclofenac	1	clopidogrel, diclofenac. Either increases effects of the other by pharmacodynamic synergism. Modify Therapy/Monitor Closely. Clopidogrel and NSAIDs both inhibit platelet aggregation
clopidogrel + verapamil	1	verapamil will decrease the level or effect of clopidogrel by affecting hepatic/intestinal enzyme CYP3A4 metabolism. Use Caution/Monitor. Inhibition of CYP3A4 will reduce clopidogrel bioactivation
dapagliflozin + glimepiride	1	glimepiride, dapagliflozin. Either increases effects of the other by pharmacodynamic synergism. Modify Therapy/Monitor Closely. Consider a lower dose of insulin or insulin secretagogue to avoid hypoglycemia when coadministered with dapagliflozin
dexamethasone + fluconazole	1	fluconazole will increase the level or effect of dexamethasone by affecting hepatic/intestinal enzyme CYP3A4 metabolism. Use Caution/Monitor
dexamethasone + levofloxacin	1	dexamethasone and levofloxacin both increase Other (see comment). Use Caution/Monitor. Coadministration of quinolone antibiotics and corticosteroids may increase risk of tendon rupture
dexamethasone + rivaroxaban	1	dexamethasone decreases levels of rivaroxaban by affecting hepatic/intestinal enzyme CYP3A4 metabolism. Use Caution/Monitor. Avoid concomitant use of rivaroxaban with drugs that are combined P-gp and strong CYP3A4 inducers. Consider increasing the rivaroxaban dose if these drugs must be coadministered
dexamethasone + theophylline	1	dexamethasone will decrease the level or effect of theophylline by affecting hepatic/intestinal enzyme CYP3A4 metabolism. Use Caution/Monitor
diclofenac + enoxaparin	1	enoxaparin and diclofenac both increase anticoagulation. Modify Therapy/Monitor Closely
diclofenac + fosinopril	1	diclofenac, fosinopril. pharmacodynamic antagonism. Avoid or Use Alternate Drug. Coadministration may result in a significant decrease in renal function. NSAIDs may diminish the antihypertensive effect of ACE inhibitors. The mechanism of these interactions is likely related to the ability of NSAIDs to reduce the synthesis of vasodilating renal prostaglandins

diclofenac + lisinopril	1	diclofenac, lisinopril. pharmacodynamic antagonism. Avoid or Use Alternate Drug. Coadministration may result in a significant decrease in renal function. NSAIDs may diminish the antihypertensive effect of ACE inhibitors. The mechanism of these interactions is likely related to the ability of NSAIDs to reduce the synthesis of vasodilating renal prostaglandins
diclofenac + prednisolone	1	diclofenac, prednisolone. Either increases toxicity of the other by pharmacodynamic synergism. Use Caution/Monitor. Increased risk of GI ulceration
digoxin + esomeprazole	1	esomeprazole increases toxicity of digoxin by Other (see comment). Use Caution/Monitor. Comment: Prolonged use of PPIs may cause hypomagnesemia and increase risk for digoxin toxicity
digoxin + felodipine	1	felodipine will increase the level or effect of digoxin by P-glycoprotein (MDR1) efflux transporter. Use Caution/Monitor
digoxin + fosinopril	1	felodipine will increase the level or effect of digoxin by P-glycoprotein (MDR1) efflux transporter. Use Caution/Monitor
digoxin + levofloxacin	1	levofloxacin will increase the level or effect of digoxin by altering intestinal flora. Applies only to oral form of both agents. Use Caution/Monitor
digoxin + meloxicam	1	meloxicam and digoxin both increase serum potassium. Use Caution/Monitor
digoxin + memantine	1	digoxin will increase the level or effect of memantine by basic (cationic) drug competition for renal tubular clearance. Use Caution/Monitor
digoxin + metoclopramide	1	metoclopramide decreases levels of digoxin by inhibition of GI absorption. Applies only to oral form of both agents. Use Caution/Monitor
digoxin + moxifloxacin	1	moxifloxacin will increase the level or effect of digoxin by altering intestinal flora. Applies only to oral form of both agents. Use Caution/Monitor
digoxin + sacubitril/valsartan	1	sacubitril/valsartan and digoxin both increase serum potassium. Use Caution/Monitor
digoxin + salmeterol	1	digoxin increases and salmeterol decreases serum potassium. Effect of interaction is not clear, use caution. Use Caution/Monitor
digoxin + valsartan	1	valsartan will increase the level or effect of digoxin by decreasing renal clearance. Use Caution/Monitor. Monitor digoxin levels closely when coadministered with drugs that may decrease glomerular filtration or tubular secretion
digoxin + vancomycin	1	vancomycin will increase the level or effect of digoxin by altering intestinal flora. Applies only to oral form of both agents. Use Caution/Monitor
diltiazem + magnesium supplement	1	magnesium supplement, diltiazem. Either increases toxicity of the other by pharmacodynamic synergism. Use Caution/Monitor. Calcium channel blockers may increase toxic effects of magnesium; magnesium may increase hypotensive effects of calcium channel blockers
diphenhydramine + tamsulosin	1	diphenhydramine increases levels of tamsulosin by affecting hepatic enzyme CYP2D6 metabolism. Use Caution/Monitor
doxazosin + lisinopril	1	lisinopril, doxazosin. Mechanism: pharmacodynamic synergism. Use Caution/Monitor. Exaggerated first dose hypotensive response
doxazosin + perindopril	1	perindopril, doxazosin. Mechanism: pharmacodynamic synergism. Use Caution/Monitor. Exaggerated first dose hypotensive response

dulaglutide + metformin	1	dulaglutide, metformin. Either increases effects of the other by pharmacodynamic synergism. Use Caution/Monitor. Antidiabetic agents are often used in combination; dosage adjustments may be required when initiating or discontinuing antidiabetic agents
enalapril + eplerenone	1	enalapril, eplerenone. Mechanism: pharmacodynamic synergism. Use Caution/Monitor. Risk of hyperkalemia
enalapril + ketorolac	1	enalapril, ketorolac. Either increases toxicity of the other by Other (see comment). Use Caution/Monitor. Comment: May result in renal function deterioration, particularly in elderly or volume depleted individuals
enalapril + meloxicam	1	enalapril, meloxicam. Either increases toxicity of the other by Other (see comment). Use Caution/Monitor. Comment: May result in renal function deterioration, particularly in elderly or volume depleted individuals
enalapril + sodium bicarbonate	1	sodium bicarbonate decreases effects of enalapril by unspecified interaction mechanism. Use Caution/Monitor
enoxaparin + prednisolone	1	prednisolone, enoxaparin. Other (see comment). Use Caution/Monitor. Comment: Corticosteroids may decrease anticoagulant effects by increasing blood coagulability; conversely, they may impair vascular integrity, thus increasing bleeding risk. Monitor INR closely
enoxaparin + sacubitril/valsartan	1	enoxaparin increases toxicity of sacubitril/valsartan by Other (see comment). Use Caution/Monitor. Comment: Low molecular weight heparins may suppress adrenal aldosterone secretion, which can potentially cause hyperkalemia
enoxaparin + ticagrelor	1	ticagrelor, enoxaparin. Either increases effects of the other by anticoagulation. Use Caution/Monitor. Increased risk of bleeding during concomitant use of medications that increase potential for bleeding
eplerenone + lisinopril	1	lisinopril, eplerenone. Mechanism: pharmacodynamic synergism. Use Caution/Monitor. Risk of hyperkalemia
eplerenone + ramipril	1	ramipril, eplerenone. Mechanism: pharmacodynamic synergism. Use Caution/Monitor. Risk of hyperkalemia
eplerenone + verapamil	1	verapamil will increase the level or effect of eplerenone by affecting hepatic/intestinal enzyme CYP3A4 metabolism. Use Caution/Monitor. Plasma concentrations and pharmacologic or toxic effects of eplerenone may be increased by verapamil
escitalopram + omeprazole	1	omeprazole will increase the level or effect of escitalopram by affecting hepatic enzyme CYP2C19 metabolism. Use Caution/Monitor
felodipine + metoprolol	1	metoprolol and felodipine both increase anti-hypertensive channel blocking. Modify Therapy/Monitor Closely
ferrous sulfate + pantoprazole	1	pantoprazole will decrease the level or effect of ferrous sulfate by increasing gastric pH. Applies only to oral form of both agents. Use Caution/Monitor
fluconazole + levofloxacin	1	fluconazole and levofloxacin both increase QTc interval. Modify Therapy/Monitor Closely
fluconazole + rivaroxaban	1	fluconazole increases levels of rivaroxaban by affecting hepatic/intestinal enzyme CYP3A4 metabolism. Use Caution/Monitor. Patients with renal impairment receiving rivaroxaban with moderate CYP3A4 inhibitors may have significant increases in exposure compared with patients with normal renal function and no inhibitor use; increased may increase bleeding risk

formoterol + indapamide	1	formoterol and indapamide both decrease serum potassium. Use Caution/Monitor
formoterol + moxonidine	1	moxonidine increases and formoterol decreases sedation. Effect of interaction is not clear, use caution. Use Caution/Monitor
fosinopril + furosemide	1	fosinopril, furosemide. Mechanism: pharmacodynamic synergism. Use Caution/Monitor. Risk of acute hypotension, renal insufficiency
fosinopril + heparin	1	heparin increases toxicity of fosinopril by Other (see comment). Use Caution/Monitor. Comment: Low molecular weight heparins may suppress adrenal aldosterone secretion, which can potentially cause hyperkalemia
furosemide + insulin	1	furosemide decreases effects of insulin degludec by Other (see comment). Use Caution/Monitor. Comment: Diuretics may cause hyperglycemia and glycosuria in patients with diabetes mellitus, possibly by diuretic-induced hypokalemia
furosemide + lisinopril	1	lisinopril, furosemide. Mechanism: pharmacodynamic synergism. Use Caution/Monitor. Risk of acute hypotension, renal insufficiency
gentamicin + ketorolac	1	ketorolac increases and gentamicin decreases serum potassium. Effect of interaction is not clear, use caution. Use Caution/Monitor
heparin + meloxicam	1	heparin and meloxicam both increase anticoagulation. Modify Therapy/Monitor Closely
heparin + perindopril	1	heparin increases toxicity of perindopril by Other (see comment). Use Caution/Monitor. Comment: Low molecular weight heparins may suppress adrenal aldosterone secretion, which can potentially cause hyperkalemia
heparin + telmisartan	1	heparin increases toxicity of telmisartan by Other (see comment). Use Caution/Monitor. Comment: Low molecular weight heparins may suppress adrenal aldosterone secretion, which can potentially cause hyperkalemia
heparin + valsartan	1	heparin increases toxicity of valsartan by Other (see comment). Use Caution/Monitor. Comment: Low molecular weight heparins may suppress adrenal aldosterone secretion, which can potentially cause hyperkalemia
hydrochlorothiazide + losartan	1	losartan increases and hydrochlorothiazide decreases serum potassium. Effect of interaction is not clear, use caution. Use Caution/Monitor
hydrochlorothiazide + potassium	1	potassium chloride increases and hydrochlorothiazide decreases serum potassium. Effect of interaction is not clear, use caution. Modify Therapy/Monitor Closely
hydrochlorothiazide + sotalol	1	sotalol increases and hydrochlorothiazide decreases serum potassium. Effect of interaction is not clear, use caution. Use Caution/Monitor
hydrochlorothiazide + spironolactone	1	spironolactone increases and hydrochlorothiazide decreases serum potassium. Effect of interaction is not clear, use caution. Modify Therapy/Monitor Closely
ibuprofen + torsemide	1	ibuprofen increases and torsemide decreases serum potassium. Effect of interaction is not clear, use caution. Use Caution/Monitor
ibuprofen + warfarin	1	ibuprofen, warfarin. Either increases effects of the other by pharmacodynamic synergism. Modify Therapy/Monitor Closely. Drugs with antiplatelet properties may increase anticoagulation effect of warfarin
indapamide + ketorolac	1	ibuprofen, warfarin. Either increases effects of the other by pharmacodynamic synergism. Modify Therapy/Monitor Closely. Drugs with antiplatelet properties may increase anticoagulation effect of warfarin

indapamide + timolol	1	timolol increases and indapamide decreases serum potassium. Effect of interaction is not clear, use caution. Use Caution/ Monitor
indapamide + valsartan	1	valsartan increases and indapamide decreases serum potassium. Effect of interaction is not clear, use caution. Use Caution/Monitor
insulin + perindopril	1	perindopril increases effects of insulin aspart by pharmacodynamic synergism. Use Caution/Monitor
insulin + telmisartan	1	telmisartan increases effects of insulin aspart by unspecified interaction mechanism. Use Caution/Monitor. Concomitant use of insulin and ARBs may require insulin dosage adjustment and increased glucose monitoring
insulin + torsemide	1	torsemide decreases effects of insulin degludec by Other (see comment). Use Caution/Monitor. Comment: Diuretics may cause hyperglycemia and glycosuria in patients with diabetes mellitus, possibly by diuretic-induced hpokalemia
ketoprofen + ramipril	1	ramipril, ketoprofen. Either increases toxicity of the other by Other (see comment). Use Caution/Monitor. Comment: May result in renal function deterioration, particularly in elderly or volume depleted individuals
ketorolac + levofloxacin	1	levofloxacin, ketorolac. Other (see comment). Modify Therapy/Monitor Closely. Comment: Risk of CNS stimulation/seizure. Mechanism: Displacement of GABA from receptors in brain
ketorolac + prednisolone	1	ketorolac, prednisolone. Either increases toxicity of the other by pharmacodynamic synergism. Use Caution/Monitor. Increased risk of GI ulceration
ketorolac + rivaroxaban	1	rivaroxaban, ketorolac. Other (see comment). Use Caution/Monitor. Comment: NSAIDs are known to increase bleeding. Bleeding risk may be increased when NSAIDs are used concomitantly with rivaroxaban. Monitor for signs/symptoms of blood loss
levocarnitine + warfarin	1	levocarnitine increases effects of warfarin by unspecified interaction mechanism. Use Caution/Monitor
levofloxacin + magnesium sulfate	1	magnesium sulfate decreases levels of levofloxacin by inhibition of GI absorption. Applies only to oral form of both agents. Use Caution/Monitor. Separate by 2 hours
levofloxacin + moxifloxacin	1	levofloxacin and moxifloxacin both increase QTc interval. Modify Therapy/Monitor Closely
levothyroxine + magnesium supplement	1	magnesium supplement will decrease the level or effect of levothyroxine by Other (see comment). Modify Therapy/Monitor Closely. Drug may adsorb to magnesium; may decrease absorption by the intestinal tract; applies to oral forms; may separate administration of drugs by 4 hr
levothyroxine + warfarin	1	levothyroxine increases effects of warfarin by unspecified interaction mechanism. Use Caution/Monitor
lisinopril + meloxicam	1	lisinopril, meloxicam. Either increases toxicity of the other by Other (see comment). Use Caution/Monitor. Comment: May result in renal function deterioration, particularly in elderly or volume depleted individuals
losartan + lornoxicam	1	losartan and lornoxicam both increase serum potassium. Use Caution/Monitor
losartan + sacubitril/valsartan	1	losartan and sacubitril/valsartan both increase serum potassium. Use Caution/Monitor
losartan + tizanidine	1	tizanidine increases effects of losartan by pharmacodynamic synergism. Use Caution/Monitor. Risk of hypotension

magnesium supplement + nifedipine	1	magnesium supplement, nifedipine. Either increases toxicity of the other by Other (see comment). Use Caution/Monitor. Comment: Calcium channel blockers may increase toxic effects of magnesium; magnesium may increase hypotensive effects of calcium channel blockers
magnesium supplement + potassium	1	magnesium supplement will decrease the level or effect of potassium phosphate by Other (see comment). Modify Therapy/Monitor Closely. Drug may form a chelate with divalent cations; may decrease absorption by the intestinal tract; applies to oral forms; separate administration of drugs to avoid interaction
meloxicam + torsemide	1	meloxicam increases and torsemide decreases serum potassium. Effect of interaction is not clear, use caution. Use Caution/Monitor
meloxicam + warfarin	1	meloxicam, warfarin. Either increases effects of the other by pharmacodynamic synergism. Modify Therapy/Monitor Closely. Drugs with antiplatelet properties may increase anticoagulation effect of warfarin
metformin + nifedipine	1	nifedipine decreases effects of metformin by pharmacodynamic antagonism. Use Caution/Monitor. Patient should be closely observed for loss of blood glucose control; when drugs are withdrawn from a patient receiving metformin, patient should be observed closely for hypoglycemia
metformin + ramipril	1	ramipril increases toxicity of metformin by unspecified interaction mechanism. Use Caution/Monitor. Increases risk for hypoglycemia and lactic acidosis
methotrexate + omeprazole	1	omeprazole increases levels of methotrexate by decreasing renal clearance. Use Caution/Monitor. Temporary withdrawal of PPI may be considered in some patients
metoprolol + nebivolol	1	metoprolol and nebivolol both increase anti-hypertensive channel blocking. Avoid or Use Alternate Drug
mifepristone + quinine	1	mifepristone, quinine. QTc interval. Modify Therapy/Monitor Closely. Use alternatives if available
moxifloxacin + prednisolone	1	prednisolone and moxifloxacin both increase Other (see comment). Use Caution/Monitor. Coadministration of quinolone antibiotics and corticosteroids may increase risk of tendon rupture
neostigmine + pyridostigmine	1	neostigmine and pyridostigmine both increase cholinergic effects/transmission. Use Caution/Monitor
nifedipine + sotalol	1	sotalol and nifedipine both increase anti-hypertensive channel blocking. Modify Therapy/Monitor Closely
nitroglycerin + verapamil	1	verapamil, nitroglycerin sublingual. Either increases toxicity of the other by additive vasodilation. Modify Therapy/Monitor Closely. Marked orthostatic hypotension reported with concomitant use
pentoxifylline + warfarin	1	pentoxifylline increases effects of warfarin by anticoagulation. Use Caution/Monitor
potassium + telmisartan	1	telmisartan and potassium chloride both increase serum potassium. Use Caution/Monitor
propranolol + sotalol	1	propranolol and sotalol both increase serum potassium. Use Caution/Monitor
rivaroxaban + saw palmetto	1	saw palmetto increases toxicity of rivaroxaban by unspecified interaction mechanism. Use Caution/Monitor. May increase risk of bleeding
rivaroxaban + verapamil	1	verapamil increases levels of rivaroxaban by affecting hepatic/intestinal enzyme CYP3A4 metabolism. Use Caution/Monitor. Verapamil also inhibits P-gp activity, which can further increase rivaroxaban serum levels; since both pathways of rivaroxaban elimination are affected,

		patients with renal impairment receiving rivaroxaban with drugs that are combined P-gp and moderate CYP3A4 inhibitors may increase exposure compared to patients with normal renal function; monitor for bleeding
salmeterol + spironolactone	1	spironolactone increases and salmeterol decreases serum potassium. Effect of interaction is not clear, use caution. Modify Therapy/Monitor Closely
salmeterol + torsemide	1	salmeterol and torsemide both decrease serum potassium. Use Caution/Monitor

Note: Impact of drug–drug interactions associated with the combinations “aspirin + captopril” and “aspirin + enalapril” may be considered insignificant due to the use of low-dose aspirin in the majority of cases. Administration of aspirin at doses less than 300 mg per day has little effect on the effectiveness of captopril and enalapril. Administration of aspirin in higher doses reduces the effectiveness of captopril and enalapril.

Table S7. Drug combinations resulting in potential minor drug–drug interactions in the list of taken medications in patients with cardiovascular diseases according to data derived from the electronic health records (n = 1030) established in 2018–2023.

Drug combinations	n	Potential minor drug-drug interactions (T-list)
aspirin + furosemide	25	aspirin decreases effects of furosemide by pharmacodynamic antagonism. Minor/Significance Unknown. NSAIDs decrease prostaglandin synthesis
clopidogrel + torsemide	22	clopidogrel increases levels of torsemide by decreasing metabolism. Minor/Significance Unknown
magnesium hydroxide + spironolactone	21	spironolactone increases levels of magnesium hydroxide by decreasing renal clearance. Minor/Significance Unknown
magnesium hydroxide + torsemide	20	torsemide decreases levels of magnesium hydroxide by increasing renal clearance. Minor/Significance Unknown
aspirin + indapamide	18	indapamide will increase the level or effect of aspirin by acidic (anionic) drug competition for renal tubular clearance. Minor/Significance Unknown
aspirin + eplerenone	10	aspirin decreases effects of eplerenone by pharmacodynamic antagonism. Minor/Significance Unknown. NSAIDs decrease prostaglandin synthesis
dexamethasone + omeprazole	10	dexamethasone will decrease the level or effect of omeprazole by affecting hepatic/intestinal enzyme CYP3A4 metabolism. Minor/Significance Unknown
ascorbic acid + aspirin	9	ascorbic acid will increase the level or effect of aspirin by acidic (anionic) drug competition for renal tubular clearance. Minor/Significance Unknown
ascorbic acid + aspirin	9	ascorbic acid increases levels of aspirin by decreasing renal clearance. Minor/Significance Unknown
aspirin + hydrochlorothiazide	9	hydrochlorothiazide will increase the level or effect of aspirin by acidic (anionic) drug competition for renal tubular clearance. Minor/Significance Unknown
aspirin + prednisolone	7	prednisolone decreases levels of aspirin by increasing renal clearance. Minor/Significance Unknown
ceftriaxone + furosemide	7	ceftriaxone increases toxicity of furosemide by pharmacodynamic synergism. Minor/Significance Unknown. Increased risk of nephrotoxicity
furosemide + magnesium hydroxide	7	furosemide decreases levels of magnesium hydroxide by increasing renal clearance. Minor/Significance Unknown
furosemide + prednisolone	7	prednisolone, furosemide. Mechanism: pharmacodynamic synergism. Minor/Significance Unknown. Risk of hypokalemia, especially with strong glucocorticoid activity
hydrochlorothiazide + magnesium hydroxide	7	hydrochlorothiazide decreases levels of magnesium hydroxide by increasing renal clearance. Minor/Significance Unknown
indapamide + magnesium hydroxide	7	indapamide decreases levels of magnesium hydroxide by increasing renal clearance. Minor/Significance Unknown
indapamide + metformin	7	indapamide decreases effects of metformin by pharmacodynamic antagonism. Minor/Significance Unknown. Thiazide dosage >50 mg/day may increase blood glucose
furosemide + metformin	6	furosemide increases levels of metformin by unspecified interaction mechanism. Minor/Significance Unknown. Patient should be closely observed for loss of blood glucose control; when drugs are withdrawn from a patient receiving metformin, patient should be observed closely for hypoglycemia

acetaminophen + enoxaparin	5	acetaminophen increases effects of enoxaparin by unknown mechanism. Minor/Significance Unknown
budesonide + furosemide	5	budesonide, furosemide. Mechanism: pharmacodynamic synergism. Minor/Significance Unknown. Risk of hypokalemia, especially with strong glucocorticoid activity
dexamethasone + torsemide	5	dexamethasone, torsemide. Mechanism: pharmacodynamic synergism. Minor/Significance Unknown. Risk of hypokalemia, especially with strong glucocorticoid activity
prednisolone + torsemide	5	prednisolone, torsemide. Mechanism: pharmacodynamic synergism. Minor/Significance Unknown. Risk of hypokalemia, especially with strong glucocorticoid activity
acetaminophen + heparin	4	acetaminophen increases effects of heparin by unknown mechanism. Minor/Significance Unknown
acetazolamide + aspirin	4	aspirin will decrease the level or effect of acetazolamide by affecting hepatic/intestinal enzyme CYP3A4 metabolism. Minor/Significance Unknown
acetazolamide + omeprazole	4	acetazolamide will increase the level or effect of omeprazole by affecting hepatic/intestinal enzyme CYP3A4 metabolism. Minor/Significance Unknown
aspirin + dexamethasone	4	dexamethasone decreases levels of aspirin by increasing renal clearance. Minor/Significance Unknown
cyanocobalamin + omeprazole	4	omeprazole decreases levels of cyanocobalamin by inhibition of GI absorption. Applies only to oral form of both agents. Minor/Significance Unknown
dexamethasone + furosemide	4	dexamethasone, furosemide. Mechanism: pharmacodynamic synergism. Minor/Significance Unknown. Risk of hypokalemia, especially with strong glucocorticoid activity
dexamethasone + insulin	4	dexamethasone decreases effects of insulin regular human by pharmacodynamic antagonism. Minor/Significance Unknown
		dexamethasone decreases effects of insulin lispro by pharmacodynamic antagonism. Minor/Significance Unknown
		dexamethasone decreases effects of insulin detemir by pharmacodynamic antagonism. Minor/Significance Unknown
acetazolamide + rivaroxaban	3	acetazolamide will increase the level or effect of rivaroxaban by affecting hepatic/intestinal enzyme CYP3A4 metabolism. Minor/Significance Unknown
aspirin + chlorthalidone	3	chlorthalidone will increase the level or effect of aspirin by acidic (anionic) drug competition for renal tubular clearance. Minor/Significance Unknown
aspirin + verapamil	3	verapamil increases effects of aspirin by unknown mechanism. Minor/Significance Unknown. Enhanced antiplatelet activity
chlorthalidone + magnesium hydroxide	3	chlorthalidone decreases levels of magnesium hydroxide by increasing renal clearance. Minor/Significance Unknown
digoxin + magnesium hydroxide	3	digoxin decreases levels of magnesium hydroxide by increasing renal clearance. Minor/Significance Unknown
insulin + potassium	3	potassium chloride increases effects of insulin aspart by pharmacodynamic synergism. Minor/Significance Unknown. Interaction especially seen in the treatment of hypokalemia
acetazolamide + atorvastatin	2	acetazolamide will increase the level or effect of atorvastatin by affecting hepatic/intestinal enzyme CYP3A4 metabolism. Minor/Significance Unknown
acetazolamide + budesonide	2	acetazolamide will increase the level or effect of budesonide by affecting hepatic/intestinal enzyme CYP3A4 metabolism. Minor/Significance Unknown

amikacin + aspirin	2	aspirin increases levels of amikacin by decreasing renal clearance. Minor/Significance Unknown. Interaction mainly occurs in preterm infants
amlodipine + dexamethasone	2	dexamethasone will decrease the level or effect of amlodipine by affecting hepatic/intestinal enzyme CYP3A4 metabolism. Minor/Significance Unknown
aspirin + cyanocobalamin	2	aspirin decreases levels of cyanocobalamin by inhibition of GI absorption. Applies only to oral form of both agents. Minor/Significance Unknown
aspirin + diclofenac	2	aspirin and diclofenac both increase anticoagulation. Use Caution/Monitor
aspirin + ketorolac	2	aspirin will increase the level or effect of ketorolac by acidic (anionic) drug competition for renal tubular clearance. Minor/Significance Unknown
carbamazepine + omeprazole	2	omeprazole increases levels of carbamazepine by decreasing metabolism. Minor/Significance Unknown. Monitor plasma levels when used concomitantly
cyclophosphamide + ondansetron	2	cyclophosphamide will increase the level or effect of ondansetron by affecting hepatic/intestinal enzyme CYP3A4 metabolism. Minor/Significance Unknown
cyclophosphamide + trimethoprim	2	cyclophosphamide will increase the level or effect of trimethoprim by affecting hepatic/intestinal enzyme CYP3A4 metabolism. Minor/Significance Unknown
dexamethasone + indapamide	2	dexamethasone, indapamide. Mechanism: pharmacodynamic synergism. Minor/Significance Unknown. Risk of hypokalemia, especially with strong glucocorticoid activity
dextrose + magnesium sulfate	2	dextrose decreases levels of magnesium sulfate by increasing renal clearance. Minor/Significance Unknown
fluconazole + sulfamethoxazole	2	fluconazole will increase the level or effect of sulfamethoxazole by affecting hepatic enzyme CYP2C9/10 metabolism. Minor/Significance Unknown
furosemide + magnesium sulfate	2	furosemide decreases levels of magnesium sulfate by increasing renal clearance. Minor/Significance Unknown
indapamide + magnesium sulfate	2	indapamide decreases levels of magnesium sulfate by increasing renal clearance. Minor/Significance Unknown
insulin + prednisolone	2	prednisolone decreases effects of insulin aspart by pharmacodynamic antagonism. Minor/Significance Unknown
magnesium sulfate + spironolactone	2	spironolactone increases levels of magnesium sulfate by decreasing renal clearance. Minor/Significance Unknown
metformin + potassium	2	potassium chloride increases effects of metformin by pharmacodynamic synergism. Minor/Significance Unknown. Interaction especially seen in the treatment of hypokalemia
acetaminophen + metronidazole	1	metronidazole will increase the level or effect of acetaminophen by affecting hepatic enzyme CYP2E1 metabolism. Minor/Significance Unknown
acetazolamide + amiodarone	1	acetazolamide will increase the level or effect of amiodarone by affecting hepatic/intestinal enzyme CYP3A4 metabolism. Minor/Significance Unknown
acetazolamide + dexamethasone	1	acetazolamide will increase the level or effect of dexamethasone by affecting hepatic/intestinal enzyme CYP3A4 metabolism. Minor/Significance Unknown
acetazolamide + isosorbide dinitrate	1	acetazolamide will increase the level or effect of isosorbide dinitrate by affecting hepatic/intestinal enzyme CYP3A4 metabolism. Minor/Significance Unknown
acetazolamide + losartan	1	acetazolamide will increase the level or effect of losartan by affecting hepatic/intestinal enzyme CYP3A4 metabolism. Minor/Significance Unknown

acyclovir + indapamide	1	indapamide will increase the level or effect of acyclovir by acidic (anionic) drug competition for renal tubular clearance. Minor/Significance Unknown
amikacin + magnesium hydroxide	1	amikacin decreases levels of magnesium hydroxide by increasing renal clearance. Minor/Significance Unknown
amiodarone + diclofenac	1	amiodarone will increase the level or effect of diclofenac by affecting hepatic enzyme CYP2C9/10 metabolism. Minor/Significance Unknown
amitriptyline + carbamazepine	1	carbamazepine decreases levels of amitriptyline by increasing metabolism. Minor/Significance Unknown
amoxicillin + clarithromycin	1	clarithromycin decreases effects of amoxicillin by pharmacodynamic antagonism. Minor/Significance Unknown
aspirin + calcium carbonate	1	calcium carbonate, aspirin. Mechanism: passive renal tubular reabsorption due to increased pH. Minor/ Significance Unknown. Salicylate levels increased at moderate doses; salicylate levels decreased at large doses (d/ t increased renal excretion of unchanged salicylic acid)
aspirin + folic acid	1	aspirin decreases levels of folic acid by inhibition of GI absorption. Applies only to oral form of both agents. Minor/Significance Unknown
aspirin + meloxicam	1	aspirin will increase the level or effect of meloxicam by acidic (anionic) drug competition for renal tubular clearance. Minor/Significance Unknown
aspirin + sodium bicarbonate	1	sodium bicarbonate, aspirin. Mechanism: passive renal tubular reabsorption due to increased pH. Minor/Significance Unknown. Salicylate levels increased at moderate doses; salicylate levels decreased at large doses (d/t increased renal excretion of unchanged salicylic acid)
aspirin + vancomycin	1	aspirin increases levels of vancomycin by decreasing renal clearance. Minor/Significance Unknown. Interaction mainly occurs in neonates
atorvastatin + cyclophosphamide	1	cyclophosphamide will increase the level or effect of atorvastatin by affecting hepatic/intestinal enzyme CYP3A4 metabolism. Minor/Significance Unknown
budesonide + indapamide	1	budesonide, indapamide. Mechanism: pharmacodynamic synergism. Minor/Significance Unknown. Risk of hypokalemia, especially with strong glucocorticoid activity
budesonide + insulin	1	budesonide decreases effects of insulin aspart by pharmacodynamic antagonism. Minor/Significance Unknown
budesonide + metformin	1	budesonide decreases effects of metformin by pharmacodynamic antagonism. Minor/Significance Unknown
calcium gluconate + torsemide	1	torsemide decreases levels of calcium gluconate by increasing renal clearance. Minor/Significance Unknown
carvedilol + omeprazole	1	omeprazole will increase the level or effect of carvedilol by affecting hepatic enzyme CYP2C9/10 metabolism. Minor/Significance Unknown
chlorthalidone + metformin	1	chlorthalidone decreases effects of metformin by pharmacodynamic antagonism. Minor/Significance Unknown. Thiazide dosage >50 mg/day may increase blood glucose
clarithromycin + dexamethasone	1	dexamethasone will decrease the level or effect of clarithromycin by affecting hepatic/intestinal enzyme CYP3A4 metabolism. Minor/Significance Unknown
cyanocobalamin + metformin	1	metformin decreases levels of cyanocobalamin by unspecified interaction mechanism. Minor/Significance Unknown. It may take several years of metformin therapy to develop vitamin B12 deficiency

dexamethasone + eplerenone	1	dexamethasone will decrease the level or effect of eplerenone by affecting hepatic/intestinal enzyme CYP3A4 metabolism. Minor/Significance Unknown
dexamethasone + esomeprazole	1	dexamethasone will decrease the level or effect of esomeprazole by increasing metabolism. Minor/Significance Unknown
dexamethasone + metformin	1	dexamethasone decreases effects of metformin by pharmacodynamic antagonism. Minor/Significance Unknown
dextrose + magnesium hydroxide	1	dextrose decreases levels of magnesium hydroxide by increasing renal clearance. Minor/Significance Unknown
diclofenac + ketoprofen	1	diclofenac will increase the level or effect of ketoprofen by acidic (anionic) drug competition for renal tubular clearance. Minor/Significance Unknown
diclofenac + ketorolac	1	diclofenac will increase the level or effect of ketorolac by acidic (anionic) drug competition for renal tubular clearance. Minor/Significance Unknown
diclofenac + meloxicam	1	diclofenac will increase the level or effect of meloxicam by acidic (anionic) drug competition for renal tubular clearance. Minor/Significance Unknown
diclofenac + metronidazole	1	metronidazole will increase the level or effect of diclofenac by affecting hepatic enzyme CYP2C9/10 metabolism. Minor/Significance Unknown
diclofenac topical + diclofenac	1	diclofenac topical, diclofenac. Either increases effects of the other by pharmacodynamic synergism. Minor/Significance Unknown. Although low, there is systemic exposure to diclofenac topical; theoretically, concomitant administration with systemic NSAIDS or aspirin may result in increased NSAID adverse effects
diclofenac topical + ketoprofen	1	diclofenac topical, ketoprofen. Either increases effects of the other by pharmacodynamic synergism. Minor/Significance Unknown. Although low, there is systemic exposure to diclofenac topical; theoretically, concomitant administration with systemic NSAIDS or aspirin may result in increased NSAID adverse effects
		diclofenac topical, meloxicam. Either increases effects of the other by pharmacodynamic synergism. Minor/Significance Unknown. Although low, there is systemic exposure to diclofenac topical; theoretically, concomitant administration with systemic NSAIDS or aspirin may result in increased NSAID adverse effects
digoxin + magnesium sulfate	1	digoxin decreases levels of magnesium sulfate by increasing renal clearance. Minor/Significance Unknown
eplerenone + metronidazole	1	metronidazole will increase the level or effect of eplerenone by affecting hepatic/intestinal enzyme CYP3A4 metabolism. Minor/Significance Unknown
esomeprazole + levothyroxine	1	esomeprazole decreases levels of levothyroxine by increasing gastric pH. Applies only to oral form of both agents. Minor/Significance Unknown. Conflicting evidence regarding this interaction exists
folic acid + torsemide	1	torsemide decreases levels of folic acid by increasing renal clearance. Minor/Significance Unknown
formoterol + indapamide	1	formoterol, indapamide. Mechanism: pharmacodynamic synergism. Minor/Significance Unknown. Hypokalemia
gentamicin + ketorolac	1	ketorolac increases levels of gentamicin by decreasing renal clearance. Minor/Significance Unknown. Interaction mainly occurs in preterm infants

hydrochlorothiazide + insulin	1	hydrochlorothiazide decreases effects of insulin aspart by pharmacodynamic antagonism. Minor/Significance Unknown. Thiazide dosage >50 mg/day may increase blood glucose
indapamide + insulin	1	indapamide decreases effects of insulin glargine by pharmacodynamic antagonism. Minor/Significance Unknown. Thiazide dosage >50 mg/day may increase blood glucose
indapamide + ketorolac	1	indapamide will increase the level or effect of aspirin by acidic (anionic) drug competition for renal tubular clearance. Minor/Significance Unknown
indapamide + vildagliptin	1	indapamide decreases effects of vildagliptin by pharmacodynamic antagonism. Minor/Significance Unknown. Thiazide dosage >50 mg/day may increase blood glucose
ketoprofen + meloxicam	1	ketoprofen will increase the level or effect of meloxicam by acidic (anionic) drug competition for renal tubular clearance. Minor/Significance Unknown
ketorolac + meloxicam	1	ketorolac will increase the level or effect of meloxicam by acidic (anionic) drug competition for renal tubular clearance. Minor/Significance Unknown
levothyroxine + omeprazole	1	omeprazole decreases levels of levothyroxine by increasing gastric pH. Applies only to oral form of both agents. Minor/Significance Unknown. Conflicting evidence regarding this interaction exists
lisinopril + spironolactone	1	lisinopril, spironolactone. Mechanism: pharmacodynamic synergism. Use Caution/Monitor. Risk of hyperkalemia
magnesium sulfate + torsemide	1	torsemide decreases levels of magnesium sulfate by increasing renal clearance. Minor/Significance Unknown
metformin + nifedipine	1	nifedipine increases levels of metformin by enhancing GI absorption. Applies only to oral form of both agents. Minor/Significance Unknown
metformin + prednisolone	1	prednisolone decreases effects of metformin by pharmacodynamic antagonism. Minor/Significance Unknown
omeprazole + theophylline	1	omeprazole increases toxicity of theophylline by Other (see comment). Minor/Significance Unknown. Comment: Prolonged use of proton pump inhibitors can cause hypochlorhydria, which in turn causes peristalsis in small intestine to increase and peristalsis in the proximal colon to decrease; monitor for toxicity
pyridoxine + theophylline	1	theophylline decreases levels of pyridoxine by altering metabolism. Minor/Significance Unknown

Table S8. Drug combinations resulting in potential contraindicated drug–drug interactions in the list of prescribed medications in patients with cardiovascular diseases according to data derived from the electronic health records (*n* = 1030) established in 2018–2023.

Drug combinations	n	Potential contraindicated drug-drug interactions (P-list)
captopril + sacubitril/valsartan	10	sacubitril/valsartan, captopril. Either increases toxicity of the other by Other (see comment). Contraindicated. Comment: Coadministration of neprilysin inhibitors (eg, sacubitril) with ACE inhibitors may increase angioedema risk; do not administer ACE inhibitors within 36 hr of switching to or from sacubitril/valsartan
amitriptyline + indapamide	1	amitriptyline and indapamide both increase QTc interval. Contraindicated
apixaban + carbamazepine	1	carbamazepine will decrease the level or effect of apixaban by affecting hepatic/intestinal enzyme CYP3A4 metabolism. Contraindicated. Reduces anticoagulant effect by decreasing apixaban systemic exposure
lisinopril + sacubitril/valsartan	1	sacubitril/valsartan, lisinopril. Either increases toxicity of the other by Other (see comment). Contraindicated. Comment: Coadministration of neprilysin inhibitors (eg, sacubitril) with ACE inhibitors may increase angioedema risk; do not administer ACE inhibitors within 36 hr of switching to or from sacubitril/valsartan

Table S9. Drug combinations resulting in potential serious drug–drug interactions in the list of prescribed medications in patients with cardiovascular diseases according to data derived from the electronic health records (*n* = 1030) established in 2018–2023.

Drug combinations	n	Potential serious drug-drug interactions (P-list)
aspirin + captopril *	84	aspirin, captopril. pharmacodynamic antagonism. Avoid or Use Alternate Drug. Coadministration may result in a significant decrease in renal function. NSAIDs may diminish the antihypertensive effect of ACE inhibitors. The mechanism of these interactions is likely related to the ability of NSAIDs to reduce the synthesis of vasodilating renal prostaglandins
aspirin + perindopril	74	aspirin, perindopril. pharmacodynamic antagonism. Avoid or Use Alternate Drug. Coadministration may result in a significant decrease in renal function. NSAIDs may diminish the antihypertensive effect of ACE inhibitors. The mechanism of these interactions is likely related to the ability of NSAIDs to reduce the synthesis of vasodilating renal prostaglandins
aspirin + lisinopril	58	aspirin, lisinopril. pharmacodynamic antagonism. Avoid or Use Alternate Drug. Coadministration may result in a significant decrease in renal function. NSAIDs may diminish the antihypertensive effect of ACE inhibitors. The mechanism of these interactions is likely related to the ability of NSAIDs to reduce the synthesis of vasodilating renal prostaglandins
clopidogrel + omeprazole	41	omeprazole decreases effects of clopidogrel by affecting hepatic enzyme CYP2C19 metabolism. Avoid or Use Alternate Drug. Clopidogrel efficacy may be reduced by drugs that inhibit CYP2C19. Inhibition of platelet aggregation by clopidogrel is entirely due to an active metabolite. Clopidogrel is metabolized to this active metabolite in part by CYP2C19
aspirin + fosinopril	28	aspirin, fosinopril. pharmacodynamic antagonism. Avoid or Use Alternate Drug. Coadministration may result in a significant decrease in renal function. NSAIDs may diminish the antihypertensive effect of ACE inhibitors. The mechanism of these interactions is likely related to the ability of NSAIDs to reduce the synthesis of vasodilating renal prostaglandins
aspirin + enalapril *	20	aspirin, enalapril. pharmacodynamic antagonism. Avoid or Use Alternate Drug. Coadministration may result in a significant decrease in renal function. NSAIDs may diminish the antihypertensive effect of ACE inhibitors. The mechanism of these interactions is likely related to the ability of NSAIDs to reduce the synthesis of vasodilating renal prostaglandins
apixaban + clopidogrel	18	clopidogrel and apixaban both increase anticoagulation. Avoid or Use Alternate Drug
bisoprolol + digoxin	16	bisoprolol increases effects of digoxin by pharmacodynamic synergism. Use Caution/Monitor. Enhanced bradycardia
captopril + losartan	15	losartan, captopril. Either increases toxicity of the other by pharmacodynamic synergism. Avoid or Use Alternate Drug. Dual blockade of renin-angiotensin system increases risks of hypotension, hyperkalemia, and renal impairment
captopril + sacubitril/valsartan	10	sacubitril/valsartan, captopril. Either increases toxicity of the other by pharmacodynamic synergism. Avoid or Use Alternate Drug. Dual blockade of renin-angiotensin system increases risks of hypotension, hyperkalemia, and renal impairment
digoxin + metoprolol	9	digoxin increases toxicity of metoprolol by unspecified interaction mechanism. Avoid or Use Alternate Drug. Can increase risk of bradycardia
allopurinol + captopril	8	captopril increases toxicity of allopurinol by Mechanism: unspecified interaction mechanism. Avoid or Use Alternate Drug. May increase risk for allergic or hypersensitivity reactions to

		allopurinol Monitor for symptoms of hypersensitivty reactions if both drugs must be used together
amiodarone + indapamide	6	amiodarone and indapamide both increase QTc interval. Avoid or Use Alternate Drug
aspirin + ramipril	6	aspirin, ramipril. pharmacodynamic antagonism. Avoid or Use Alternate Drug. Coadministration may result in a significant decrease in renal function. NSAIDs may diminish the antihypertensive effect of ACE inhibitors. The mechanism of these interactions is likely related to the ability of NSAIDs to reduce the synthesis of vasodilating renal prostaglandins
azilsartan + captopril	5	azilsartan, captopril. Either increases toxicity of the other by pharmacodynamic synergism. Avoid or Use Alternate Drug. Dual blockade of renin-angiotensin system increases risks of hypotension, hyperkalemia, and renal impairment
captopril + valsartan	5	sacubitril/valsartan, captopril. Either increases toxicity of the other by pharmacodynamic synergism. Avoid or Use Alternate Drug. Dual blockade of renin-angiotensin system increases risks of hypotension, hyperkalemia, and renal impairment
atorvastatin + fenofibrate	4	fenofibrate, atorvastatin. Either increases effects of the other by pharmacodynamic synergism. Avoid or Use Alternate Drug. Fenofibrate may further increase risk for rhabdomyolysis when added to optimal statin regimen to further decrease TG and increase HDLs
digoxin + omeprazole	4	omeprazole will increase the level or effect of digoxin by increasing gastric pH. Applies only to oral form of both agents. Avoid or Use Alternate Drug
allopurinol + perindopril	3	perindopril, allopurinol. Mechanism: unknown. Avoid or Use Alternate Drug. Risk of anaphylaxis, Stevens Johnson syndrome
amlodipine + nifedipine	3	amlodipine and nifedipine both increase anti-hypertensive channel blocking. Use Caution/Monitor
atorvastatin + carbamazepine	3	carbamazepine will decrease the level or effect of atorvastatin by affecting hepatic/intestinal enzyme CYP3A4 metabolism. Avoid or Use Alternate Drug
atorvastatin + clarithromycin	3	clarithromycin will increase the level or effect of atorvastatin by affecting hepatic/intestinal enzyme CYP3A4 metabolism. Avoid or Use Alternate Drug. Do not exceed atorvastatin dose of 20 mg/day when coadministered with clarithromycin
carbamazepine + omeprazole	3	carbamazepine will decrease the level or effect of omeprazole by affecting hepatic enzyme CYP2C19 metabolism. Avoid or Use Alternate Drug
clopidogrel + esomeprazole	3	esomeprazole decreases effects of clopidogrel by affecting hepatic enzyme CYP2C19 metabolism. Avoid or Use Alternate Drug. Clopidogrel efficacy may be reduced by drugs that inhibit CYP2C19. Inhibition of platelet aggregation by clopidogrel is entirely due to an active metabolite. Clopidogrel is metabolized to this active metabolite in part by CYP2C19
digoxin + pantoprazole	3	pantoprazole will increase the level or effect of digoxin by increasing gastric pH. Applies only to oral form of both agents. Avoid or Use Alternate Drug
amiodarone + clarithromycin	2	amiodarone and clarithromycin both increase QTc interval. Avoid or Use Alternate Drug
aspirin + ibuprofen	2	ibuprofen increases toxicity of aspirin by anticoagulation. Avoid or Use Alternate Drug. increases risk of bleeding
		ibuprofen decreases effects of aspirin by Other (see comment). Avoid or Use Alternate Drug. Comment: Ibuprofen decreases the antiplatelet effects of low-dose aspirin by blocking the active

		site of platelet cyclooxygenase. Administer ibuprofen 8 h before aspirin or at least 2-4 h after aspirin. The effect of other NSAIDs on aspirin is not established
candesartan + captopril	2	candesartan, captopril. Either increases toxicity of the other by pharmacodynamic synergism. Avoid or Use Alternate Drug. Dual blockade of renin-angiotensin system increases risks of hypotension, hyperkalemia, and renal impairment
captopril + ketoprofen	2	ketoprofen, captopril. pharmacodynamic antagonism. Avoid or Use Alternate Drug. Coadministration may result in a significant decrease in renal function. NSAIDs may diminish the antihypertensive effect of ACE inhibitors. The mechanism of these interactions is likely related to the ability of NSAIDs to reduce the synthesis of vasodilating renal prostaglandins
celecoxib + lisinopril	2	celecoxib, lisinopril. pharmacodynamic antagonism. Avoid or Use Alternate Drug. Coadministration may result in a significant decrease in renal function. NSAIDs may diminish the antihypertensive effect of ACE inhibitors. The mechanism of these interactions is likely related to the ability of NSAIDs to reduce the synthesis of vasodilating renal prostaglandins
clopidogrel + fondaparinux	2	fondaparinux, clopidogrel. Either increases effects of the other by pharmacodynamic synergism. Contraindicated. Enhanced risk of hemorrhage
digoxin + esomeprazole	2	esomeprazole will increase the level or effect of digoxin by increasing gastric pH. Applies only to oral form of both agents. Avoid or Use Alternate Drug
eplerenone + spironolactone	2	spironolactone, eplerenone. Mechanism: pharmacodynamic synergism. Contraindicated. Hyperkalemia
fenofibrate + rosuvastatin	2	fenofibrate, rosuvastatin. Either increases effects of the other by pharmacodynamic synergism. Avoid or Use Alternate Drug. Fenofibrate may further increase risk for rhabdomyolysis when added to optimal statin regimen to further decrease TG and increase HDLs
lisinopril + losartan	2	losartan, lisinopril. Either increases toxicity of the other by pharmacodynamic synergism. Avoid or Use Alternate Drug. Dual blockade of renin-angiotensin system increases risks of hypotension, hyperkalemia, and renal impairment
allopurinol + enalapril	1	enalapril, allopurinol. Mechanism: unknown. Avoid or Use Alternate Drug. Risk of anaphylaxis, Stevens Johnson syndrome
allopurinol + warfarin	1	allopurinol increases effects of warfarin by anticoagulation. Avoid or Use Alternate Drug
aluminum hydroxide + digoxin	1	aluminum hydroxide will increase the level or effect of digoxin by increasing gastric pH. Applies only to oral form of both agents. Avoid or Use Alternate Drug
amlodipine + simvastatin	1	amlodipine increases levels of simvastatin by Other (see comment). Avoid or Use Alternate Drug. Comment: Benefits of combination therapy should be carefully weighed against the potential risks of combination. Potential for increased risk of myopathy/rhabdomyolysis. Limit simvastatin dose to no more than 20 mg/day when used concurrently
apixaban + ketoprofen	1	ketoprofen and apixaban both increase anticoagulation. Avoid or Use Alternate Drug
aspirin + ketorolac	1	aspirin, ketorolac. Either increases toxicity of the other by pharmacodynamic synergism. Contraindicated
bisoprolol + timolol	1	bisoprolol and timolol both increase anti-hypertensive channel blocking. Avoid or Use Alternate Drug
bisoprolol + verapamil	1	verapamil, bisoprolol. Either increases toxicity of the other by unspecified interaction mechanism. Avoid or Use Alternate Drug. Can increase risk of bradycardia

budesonide + clarithromycin	1	clarithromycin will increase the level or effect of budesonide by affecting hepatic/intestinal enzyme CYP3A4 metabolism. Avoid or Use Alternate Drug
budesonide + pneumococcal vaccine polyvalent	1	budesonide decreases effects of pneumococcal vaccine polyvalent by pharmacodynamic antagonism. Contraindicated. Corticosteroids also increase risk of infection with concomitant live vaccines
captopril + ibuprofen	1	ibuprofen, captopril. pharmacodynamic antagonism. Avoid or Use Alternate Drug. Coadministration may result in a significant decrease in renal function. NSAIDs may diminish the antihypertensive effect of ACE inhibitors. The mechanism of these interactions is likely related to the ability of NSAIDs to reduce the synthesis of vasodilating renal prostaglandins
+ meloxicam	1	meloxicam, captopril. pharmacodynamic antagonism. Avoid or Use Alternate Drug. Coadministration may result in a significant decrease in renal function. NSAIDs may diminish the antihypertensive effect of ACE inhibitors. The mechanism of these interactions is likely related to the ability of NSAIDs to reduce the synthesis of vasodilating renal prostaglandins
captopril + naproxen	1	naproxen, captopril. pharmacodynamic antagonism. Avoid or Use Alternate Drug. Coadministration may result in a significant decrease in renal function. NSAIDs may diminish the antihypertensive effect of ACE inhibitors. The mechanism of these interactions is likely related to the ability of NSAIDs to reduce the synthesis of vasodilating renal prostaglandins
captopril + olmesartan	1	olmesartan, captopril. Either increases toxicity of the other by pharmacodynamic synergism. Avoid or Use Alternate Drug. Dual blockade of renin-angiotensin system increases risks of hypotension, hyperkalemia, and renal impairment
captopril + telmisartan	1	telmisartan, captopril. Either increases toxicity of the other by pharmacodynamic synergism. Avoid or Use Alternate Drug. Dual blockade of renin-angiotensin system increases risks of hypotension, hyperkalemia, and renal impairment
carbamazepine + felodipine	1	carbamazepine will decrease the level or effect of felodipine by affecting hepatic/intestinal enzyme CYP3A4 metabolism. Avoid or Use Alternate Drug
cilostazol + esomeprazole	1	esomeprazole increases toxicity of cilostazol by affecting hepatic enzyme CYP2C19 metabolism. Avoid or Use Alternate Drug. Decrease cilostazol dose by 50%; 3,4-dehydrocilostazol, an active metabolite, increased by 69% as a result of omeprazole inhibition of CYP2C19
clarithromycin + enoxaparin	1	clarithromycin increases effects of enoxaparin by decreasing metabolism. Avoid or Use Alternate Drug
clopidogrel + rabeprazole	1	rabeprazole decreases effects of clopidogrel by affecting hepatic enzyme CYP2C19 metabolism. Avoid or Use Alternate Drug. Clopidogrel efficacy may be reduced by drugs that inhibit CYP2C19. Inhibition of platelet aggregation by clopidogrel is entirely due to an active metabolite. Clopidogrel is metabolized to this active metabolite in part by CYP2C19
dabigatran + heparin	1	dabigatran and heparin both increase anticoagulation. Avoid or Use Alternate Drug. Both drugs have the potential to cause bleeding. Concomitant use may increase risk of bleeding
digoxin + nebivolol	1	digoxin, nebivolol. Either decreases toxicity of the other by unspecified interaction mechanism. Avoid or Use Alternate Drug. Can increase risk of bradycardia
digoxin + verapamil	1	verapamil increases levels of digoxin by decreasing renal clearance. Avoid or Use Alternate Drug
enalapril + meloxicam	1	meloxicam, enalapril. pharmacodynamic antagonism. Avoid or Use Alternate Drug. Coadministration may result in a significant decrease in renal function. NSAIDs may diminish

		the antihypertensive effect of ACE inhibitors. The mechanism of these interactions is likely related to the ability of NSAIDs to reduce the synthesis of vasodilating renal prostaglandins
formoterol + indapamide	1	formoterol and indapamide both increase QTc interval. Avoid or Use Alternate Drug
ibuprofen + lisinopril	1	ibuprofen, lisinopril. pharmacodynamic antagonism. Avoid or Use Alternate Drug. Coadministration may result in a significant decrease in renal function. NSAIDs may diminish the antihypertensive effect of ACE inhibitors. The mechanism of these interactions is likely related to the ability of NSAIDs to reduce the synthesis of vasodilating renal prostaglandins
ibuprofen + methotrexate	1	ibuprofen increases levels of methotrexate by decreasing renal clearance. Avoid or Use Alternate Drug. Concomitant administration of NSAIDs with high dose methotrexate has been reported to elevate and prolong serum methotrexate levels, resulting in deaths from severe hematologic and GI toxicity. NSAIDs may reduce tubular secretion of methotrexate and enhance toxicity
ivabradine + ticagrelor	1	ticagrelor will increase the level or effect of ivabradine by affecting hepatic/intestinal enzyme CYP3A4 metabolism. Avoid or Use Alternate Drug. Avoid coadministration of ivabradine with moderate CYP3A4 inhibitors
ketoprofen + perindopril	1	ketoprofen, perindopril. pharmacodynamic antagonism. Avoid or Use Alternate Drug. Coadministration may result in a significant decrease in renal function. NSAIDs may diminish the antihypertensive effect of ACE inhibitors. The mechanism of these interactions is likely related to the ability of NSAIDs to reduce the synthesis of vasodilating renal prostaglandins
ketoprofen + ramipril	1	ketoprofen, ramipril. pharmacodynamic antagonism. Avoid or Use Alternate Drug. Coadministration may result in a significant decrease in renal function. NSAIDs may diminish the antihypertensive effect of ACE inhibitors. The mechanism of these interactions is likely related to the ability of NSAIDs to reduce the synthesis of vasodilating renal prostaglandins
lisinopril + meloxicam	1	meloxicam, lisinopril. pharmacodynamic antagonism. Avoid or Use Alternate Drug. Coadministration may result in a significant decrease in renal function. NSAIDs may diminish the antihypertensive effect of ACE inhibitors. The mechanism of these interactions is likely related to the ability of NSAIDs to reduce the synthesis of vasodilating renal prostaglandins
lisinopril + sacubitril/valsartan	1	sacubitril/valsartan, lisinopril. Either increases toxicity of the other by pharmacodynamic synergism. Avoid or Use Alternate Drug. Dual blockade of renin-angiotensin system increases risks of hypotension, hyperkalemia, and renal impairment
lisinopril + valsartan	1	valsartan, lisinopril. Either increases toxicity of the other by pharmacodynamic synergism. Avoid or Use Alternate Drug. Dual blockade of renin-angiotensin system increases risks of hypotension, hyperkalemia, and renal impairment
losartan + perindopril	1	losartan, perindopril. Either increases toxicity of the other by pharmacodynamic synergism. Avoid or Use Alternate Drug. Dual blockade of renin-angiotensin system increases risks of hypotension, hyperkalemia, and renal impairment
niacin + rosuvastatin	1	niacin, rosuvastatin. Either increases toxicity of the other by pharmacodynamic synergism. Avoid or Use Alternate Drug. Increased risk of rhabdomyolysis (>1 g/day niacin)

Note: Impact of drug–drug interactions associated with the combinations "aspirin + captopril" and "aspirin + enalapril" may be considered insignificant due to the use of low-dose aspirin in the majority of cases. Administration of aspirin at doses less than 300 mg per day has little effect on the effectiveness of captopril and enalapril. Administration of aspirin in higher doses reduces the effectiveness of captopril and enalapril.

Table S10. Drug combinations resulting in potential monitor-closely drug–drug interactions in the list of prescribed medications in patients with cardiovascular diseases according to data derived from the electronic health records (n = 1030) established in 2018–2023.

Drug combinations	n	Potential monitor-closely drug-drug interactions (P-list)
aspirin + bisoprolol	412	aspirin decreases effects of bisoprolol by pharmacodynamic antagonism. Use Caution/Monitor. Long term (>1 wk) NSAID use. NSAIDs decrease prostaglandin synthesis
aspirin + spironolactone	140	spironolactone and aspirin both increase serum potassium. Modify Therapy/Monitor Closely
aspirin + losartan	129	aspirin decreases effects of losartan by pharmacodynamic antagonism. Modify Therapy/Monitor Closely. NSAIDs decrease synthesis of vasodilating renal prostaglandins, and thus affect fluid homeostasis and may diminish antihypertensive effect
bisoprolol + torsemide	117	bisoprolol increases and torsemide decreases serum potassium. Effect of interaction is not clear, use caution. Use Caution/Monitor
aspirin + metoprolol	112	aspirin decreases effects of metoprolol by pharmacodynamic antagonism. Use Caution/Monitor. Long term (>1 wk) NSAID use. NSAIDs decrease prostaglandin synthesis
aspirin + torsemide	103	aspirin increases and torsemide decreases serum potassium. Effect of interaction is not clear, use caution. Use Caution/Monitor
spironolactone + torsemide	94	spironolactone increases and torsemide decreases serum potassium. Effect of interaction is not clear, use caution. Modify Therapy/Monitor Closely
aspirin + captopril *	84	captopril, aspirin. Either increases toxicity of the other by Other (see comment). Use Caution/Monitor. Comment: May result in renal function deterioration, particularly with high dose aspirin, elderly or volume depleted individuals
aspirin + nitroglycerin	82	aspirin increases effects of nitroglycerin sublingual by additive vasodilation. Use Caution/Monitor. Vasodilatory and hemodynamic effects of NTG may be enhanced by coadministration with aspirin (additive effect desirable for emergent treatment)
aspirin + perindopril	78	perindopril, aspirin. Either increases toxicity of the other by Other (see comment). Use Caution/Monitor. Comment: May result in renal function deterioration, particularly with high doses of aspirin,in elderly or volume depleted individuals
bisoprolol + spironolactone	74	bisoprolol and spironolactone both increase serum potassium. Modify Therapy/Monitor Closely
amiodarone + atorvastatin	66	amiodarone will increase the level or effect of atorvastatin by P-glycoprotein (MDR1) efflux transporter. Use Caution/Monitor
amlodipine + bisoprolol	64	bisoprolol, amlodipine. Either increases effects of the other by pharmacodynamic synergism. Use Caution/Monitor. Both drugs lower blood pressure
aspirin + lisinopril	63	lisinopril, aspirin. Either increases toxicity of the other by Other (see comment). Use Caution/Monitor. Comment: May result in renal function deterioration, particularly with high dose aspirin, in elderly or volume depleted individuals
aspirin + clopidogrel	61	aspirin, clopidogrel. Either increases toxicity of the other by pharmacodynamic synergism. Use Caution/Monitor. The need for simultaneous use of low-dose aspirin and anticoagulant or antiplatelet agents are common for patients with cardiovascular disease; monitor closely
digoxin + spironolactone	60	spironolactone, digoxin. Mechanism: decreasing renal clearance. Use Caution/Monitor. False digoxin assay results may be obtained

captopril + torsemide	59	captopril, torsemide. Either increases toxicity of the other by Mechanism: pharmacodynamic synergism. Use Caution/Monitor. Risk of acute hypotension, renal insufficiency. Monitor blood pressure and renal function
aspirin + ticagrelor	58	aspirin, ticagrelor. Other (see comment). Use Caution/Monitor. Comment: Maintenance doses of aspirin above 100 mg decreases effectiveness of ticagrelor. Therefore, after the initial loading dose of aspirin (usually 325 mg), use ticagrelor with a maintenance dose of aspirin of 75-100 mg
bisoprolol + losartan	58	bisoprolol, losartan. Mechanism: pharmacodynamic synergism. Use Caution/Monitor. Risk of fetal compromise if given during pregnancy
atorvastatin + sacubitril/valsartan	48	losartan and bisoprolol both increase serum potassium. Use Caution/Monitor
amiodarone + bisoprolol	46	amiodarone, bisoprolol. Mechanism: pharmacodynamic synergism. Use Caution/Monitor. Risk of cardiotoxicity with bradycardia
captopril + spironolactone	45	captopril, spironolactone. Either increases toxicity of the other by Mechanism: pharmacodynamic synergism. Use Caution/Monitor. Both drugs lower blood pressure. Risk of hyperkalemia. Monitor blood pressure and potassium
metoprolol + torsemide	45	metoprolol increases and torsemide decreases serum potassium. Effect of interaction is not clear, use caution. Use Caution/Monitor
		perindopril, torsemide. Mechanism: pharmacodynamic synergism. Use Caution/Monitor. Risk of acute hypotension, renal insufficiency
digoxin + torsemide	42	digoxin increases and torsemide decreases serum potassium. Effect of interaction is not clear, use caution. Use Caution/Monitor
aspirin + indapamide	36	aspirin increases and indapamide decreases serum potassium. Effect of interaction is not clear, use caution. Use Caution/Monitor
aspirin + sacubitril/valsartan	33	aspirin decreases effects of sacubitril/valsartan by pharmacodynamic antagonism. Modify Therapy/Monitor Closely. NSAIDs decrease synthesis of vasodilating renal prostaglandins, and thus affect fluid homeostasis and may diminish antihypertensive effect
perindopril + spironolactone	33	perindopril, spironolactone. Mechanism: pharmacodynamic synergism. Use Caution/Monitor. Risk of hyperkalemia
amlodipine + nitroglycerin	32	amlodipine, nitroglycerin sublingual. Either increases toxicity of the other by additive vasodilation. Modify Therapy/Monitor Closely. Marked orthostatic hypotension reported with concomitant use
bisoprolol + digoxin	32	bisoprolol increases effects of digoxin by pharmacodynamic synergism. Use Caution/Monitor. Enhanced bradycardia
bisoprolol + indapamide	31	bisoprolol increases and indapamide decreases serum potassium. Effect of interaction is not clear, use caution. Use Caution/Monitor
lisinopril + torsemide	31	lisinopril, torsemide. Mechanism: pharmacodynamic synergism. Use Caution/Monitor. Risk of acute hypotension, renal insufficiency
amiodarone + losartan	30	amiodarone will increase the level or effect of losartan by affecting hepatic enzyme CYP2C9/10 metabolism. Use Caution/Monitor. May inhibit the conversion of losartan to its active metabolite E-3174. Importance of interaction not established; monitor individual therapeutic response to determine losartan dosage

amiodarone + metoprolol	30	amiodarone, metoprolol. Mechanism: pharmacodynamic synergism. Use Caution/Monitor. Risk of cardiotoxicity with bradycardia
		amiodarone will increase the level or effect of metoprolol by affecting hepatic enzyme CYP2D6 metabolism. Use Caution/Monitor. Monitor cardiac function carefully and observe for signs of bradycardia or heart block when amiodarone and a beta adrenergic blocker are coadministered. Amiodarone should be used with caution in patients receiving a beta adrenergic blocker, particularly if there is suspicion of underlying dysfunction of the sinus node, such as bradycardia or sick sinus syndrome, or if there is partial AV block
aspirin + fosinopril	30	fosinopril, aspirin. Either increases toxicity of the other by Other (see comment). Use Caution/Monitor. Comment: May result in renal function deterioration, particularly with high dose aspirin, in elderly or volume depleted individuals
losartan + metoprolol	30	losartan and metoprolol both increase serum potassium. Use Caution/Monitor
metoprolol + spironolactone	28	metoprolol and spironolactone both increase serum potassium. Modify Therapy/Monitor Closely
aspirin + valsartan	27	aspirin decreases effects of valsartan by pharmacodynamic antagonism. Modify Therapy/Monitor Closely. NSAIDs decrease synthesis of vasodilating renal prostaglandins, and thus affect fluid homeostasis and may diminish antihypertensive effect
aspirin + valsartan	27	valsartan and aspirin both increase serum potassium. Use Caution/Monitor
amiodarone + captopril	25	amiodarone, captopril. Either increases effects of the other by pharmacodynamic synergism. Use Caution/Monitor. Both drugs lower blood pressure. Monitor blood pressure
amiodarone + rivaroxaban	25	amiodarone increases levels of rivaroxaban by affecting hepatic/intestinal enzyme CYP3A4 metabolism. Use Caution/Monitor. Patients with renal impairment receiving rivaroxaban with drugs that are combined P-gp and weak or moderate CYP3A4 inhibitors may have significant increases in exposure compared with patients with normal renal function and no inhibitor use, since both pathways of rivaroxaban elimination are affected. Since these increases may increase bleeding risk, use rivaroxaban in this situation only if the potential benefit justifies the potential risk
losartan + torsemide	25	losartan increases and torsemide decreases serum potassium. Effect of interaction is not clear, use caution. Use Caution/Monitor
eplerenone + perindopril	24	perindopril, eplerenone. Mechanism: pharmacodynamic synergism. Use Caution/Monitor. Risk of hyperkalemia
fosinopril + torsemide	23	fosinopril, torsemide. Mechanism: pharmacodynamic synergism. Use Caution/Monitor. Risk of acute hypotension, renal insufficiency
aspirin + enalapril *	22	enalapril, aspirin. Either increases toxicity of the other by Other (see comment). Use Caution/Monitor. Comment: May result in renal function deterioration, particularly with high dose aspirin, in elderly or volume depleted individuals
aspirin + rivaroxaban	22	aspirin, rivaroxaban. Either increases toxicity of the other by anticoagulation. Use Caution/Monitor. Both drugs have the potential to cause bleeding. The need for simultaneous use of low-dose aspirin (<100 mg/day) with anticoagulants are common for patients with cardiovascular disease, but may result in increased bleeding; monitor closely. Promptly evaluate any signs or symptoms of blood loss if treated concomitantly with low-dose aspirin. Avoid coadministration with chronic use of higher dose aspirin

lisinopril + spironolactone	22	lisinopril, spironolactone. Mechanism: pharmacodynamic synergism. Use Caution/Monitor. Risk of hyperkalemia
aspirin + candesartan	21	aspirin decreases effects of candesartan by pharmacodynamic antagonism. Modify Therapy/Monitor Closely. NSAIDs decrease synthesis of vasodilating renal prostaglandins, and thus affect fluid homeostasis and may diminish antihypertensive effect
aspirin + candesartan	21	candesartan and aspirin both increase serum potassium. Use Caution/Monitor
sacubitril/valsartan + torsemide	21	sacubitril/valsartan increases and torsemide decreases serum potassium. Effect of interaction is not clear, use caution. Use Caution/Monitor
atorvastatin + valsartan	20	atorvastatin will increase the level or effect of valsartan by Other (see comment). Use Caution/Monitor. The results from an in vitro study with human liver tissue indicate that valsartan is a substrate of the hepatic uptake transporter OATP1B1; coadministration with OATP1B1 inhibitors may increase valsartan systemic exposure
		valsartan increases toxicity of atorvastatin by Other (see comment). Use Caution/Monitor. Comment: OATP1B1 inhibitors may increase risk of myopathy
atorvastatin + digoxin	19	atorvastatin will increase the level or effect of digoxin by P-glycoprotein (MDR1) efflux transporter. Use Caution/Monitor
captopril + eplerenone	19	captopril, eplerenone. Either increases toxicity of the other by Mechanism: pharmacodynamic synergism. Use Caution/Monitor. Risk of hyperkalemia. Monitor potassium
clopidogrel + pantoprazole	19	pantoprazole decreases effects of clopidogrel by affecting hepatic enzyme CYP2C19 metabolism. Use Caution/Monitor. Clopidogrel efficacy may be reduced by drugs that inhibit CYP2C19. Inhibition of platelet aggregation by clopidogrel is entirely due to the active clopidogrel metabolite. Clopidogrel is metabolized in part by CYP2C19. Pantoprazole prescribing information state that coadministration with clopidogrel had no clinically important effect on exposure to clopidogrel active metabolite; no dose adjustment of clopidogrel is required
furosemide + spironolactone	19	spironolactone increases and furosemide decreases serum potassium. Effect of interaction is not clear, use caution. Modify Therapy/Monitor Closely
bisoprolol + telmisartan	18	bisoprolol, telmisartan. Mechanism: pharmacodynamic synergism. Use Caution/Monitor. Risk of fetal compromise if given during pregnancy
		telmisartan and bisoprolol both increase serum potassium. Use Caution/Monitor
digoxin + metoprolol	18	metoprolol increases effects of digoxin by pharmacodynamic synergism. Use Caution/Monitor. Enhanced bradycardia
		metoprolol and digoxin both increase serum potassium. Use Caution/Monitor
metoprolol + sacubitril/valsartan	18	metoprolol, sacubitril/valsartan. Mechanism: pharmacodynamic synergism. Use Caution/Monitor. Risk of fetal compromise if given during pregnancy
		sacubitril/valsartan and metoprolol both increase serum potassium. Use Caution/Monitor
aspirin + azilsartan	16	aspirin, azilsartan. Either increases toxicity of the other by Other (see comment). Use Caution/Monitor. Comment: May result in renal function deterioration, particularly in elderly or volume depleted individuals
		aspirin decreases effects of azilsartan by pharmacodynamic antagonism. Modify Therapy/Monitor Closely. NSAIDs decrease synthesis of vasodilating renal prostaglandins, and thus affect fluid homeostasis and may diminish antihypertensive effect

bisoprolol + sacubitril/valsartan	16	bisoprolol, sacubitril/valsartan. Mechanism: pharmacodynamic synergism. Use Caution/Monitor. Risk of fetal compromise if given during pregnancy
		sacubitril/valsartan and bisoprolol both increase serum potassium. Use Caution/Monitor
clopidogrel + rivaroxaban	16	rivaroxaban, clopidogrel. Other (see comment). Use Caution/Monitor. Comment: Avoid concurrent administration of clopidogrel with rivaroxaban unless the benefit outweighs the risk of increased bleeding
eplerenone + sacubitril/valsartan	16	sacubitril/valsartan, eplerenone. Mechanism: pharmacodynamic synergism. Use Caution/Monitor. Risk of hyperkalemia
aspirin + furosemide	15	aspirin increases and furosemide decreases serum potassium. Effect of interaction is not clear, use caution. Use Caution/Monitor
		aspirin decreases effects of telmisartan by pharmacodynamic antagonism. Modify Therapy/Monitor Closely. NSAIDs decrease synthesis of vasodilating renal prostaglandins, and thus affect fluid homeostasis and may diminish antihypertensive effect
		telmisartan, aspirin. Either increases toxicity of the other by Other (see comment). Use Caution/Monitor. Comment: May result in renal function deterioration, particularly in elderly or volume depleted individuals
captopril + indapamide	15	indapamide, captopril. Either increases effects of the other by pharmacodynamic synergism. Use Caution/Monitor. Both drugs lower blood pressure. Increased risk of nephrotoxicity. Monitor blood pressure and renal function
ipratropium + tiotropium	15	ipratropium and tiotropium both decrease cholinergic effects/transmission. Use Caution/Monitor. Due to the poor systemic absorption of ipratropium, interaction unlikely at regularly recommended dosages
aspirin + carvedilol	14	aspirin decreases effects of carvedilol by pharmacodynamic antagonism. Use Caution/Monitor. Long term (>1 wk) NSAID use. NSAIDs decrease prostaglandin synthesis
		carvedilol and aspirin both increase serum potassium. Use Caution/Monitor
aspirin + hydrochlorothiazide	14	aspirin increases and hydrochlorothiazide decreases serum potassium. Effect of interaction is not clear, use caution. Use Caution/Monitor
aspirin + insulin	14	aspirin increases effects of insulin regular human by pharmacodynamic synergism. Modify Therapy/Monitor Closely. Coadministration of insulin with high doses of salicylates (3 g/day or more) may increase risk for hypoglycemia. Insulin dose adjustment and increased frequency of glucose monitoring may be required
bisoprolol + candesartan	14	bisoprolol, candesartan. Mechanism: pharmacodynamic synergism. Use Caution/Monitor. Risk of fetal compromise if given during pregnancy
		candesartan and bisoprolol both increase serum potassium. Use Caution/Monitor
rosuvastatin + warfarin	14	rosuvastatin increases effects of warfarin by anticoagulation. Use Caution/Monitor
atorvastatin + dabigatran	13	atorvastatin will increase the level or effect of dabigatran by P-glycoprotein (MDR1) efflux transporter. Use Caution/Monitor. Atrial fibrillation: Avoid coadministering dabigatran with P-gp inhibitors if CrCl <30 mL/min. DVT/PE treatment: Avoid coadministering dabigatran with P-gp inhibitors if CrCl <50 mL/min
enalapril + torsemide	13	enalapril, torsemide. Mechanism: pharmacodynamic synergism. Use Caution/Monitor. Risk of acute hypotension, renal insufficiency

amoxicillin + aspirin	12	amoxicillin, aspirin. Either increases levels of the other by plasma protein binding competition. Use Caution/Monitor
		amoxicillin, aspirin. Either increases levels of the other by decreasing renal clearance. Use Caution/Monitor
atorvastatin + verapamil	12	verapamil will increase the level or effect of atorvastatin by affecting hepatic/intestinal enzyme CYP3A4 metabolism. Use Caution/Monitor
		verapamil will increase the level or effect of atorvastatin by P-glycoprotein (MDR1) efflux transporter. Use Caution/Monitor
bisoprolol + formoterol	12	bisoprolol decreases effects of formoterol by pharmacodynamic antagonism. Use Caution/Monitor
		bisoprolol increases and formoterol decreases serum potassium. Effect of interaction is not clear, use caution. Use Caution/Monitor
bisoprolol + furosemide	12	bisoprolol increases and furosemide decreases serum potassium. Effect of interaction is not clear, use caution. Use Caution/Monitor
insulin + metformin	12	metformin, insulin glargine. Either increases effects of the other by pharmacodynamic synergism. Use Caution/Monitor. Antidiabetic agents are often used in combination; dosage adjustments may be required when initiating or discontinuing antidiabetic agents
losartan + spironolactone	12	losartan and spironolactone both increase serum potassium. Modify Therapy/Monitor Closely
metformin + torsemide	12	torsemide decreases effects of metformin by pharmacodynamic antagonism. Use Caution/Monitor
bisoprolol + hydrochlorothiazide	11	bisoprolol increases and hydrochlorothiazide decreases serum potassium. Effect of interaction is not clear, use caution. Use Caution/Monitor
enalapril + spironolactone	11	enalapril, spironolactone. Mechanism: pharmacodynamic synergism. Use Caution/Monitor. Risk of hyperkalemia
fosinopril + spironolactone	11	fosinopril, spironolactone. Mechanism: pharmacodynamic synergism. Use Caution/Monitor. Risk of hyperkalemia
indapamide + losartan	11	losartan increases and indapamide decreases serum potassium. Effect of interaction is not clear, use caution. Use Caution/Monitor
lisinopril + metformin	11	lisinopril increases toxicity of metformin by unspecified interaction mechanism. Use Caution/Monitor. Increases risk for hypoglycemia and lactic acidosis
losartan + omeprazole	11	omeprazole will increase the level or effect of losartan by affecting hepatic enzyme CYP2C9/10 metabolism. Use Caution/Monitor. May inhibit the conversion of losartan to its active metabolite E-3174. Importance of interaction not established; monitor individual therapeutic response to determine losartan dosage
amlodipine + metformin	10	amlodipine decreases effects of metformin by pharmacodynamic antagonism. Use Caution/Monitor. Patient should be closely observed for loss of blood glucose control; when drugs are withdrawn from a patient receiving metformin, patient should be observed closely for hypoglycemia
aspirin + heparin	10	aspirin, heparin. Either increases toxicity of the other by anticoagulation. Use Caution/Monitor. The need for simultaneous use of low-dose aspirin and anticoagulant or antiplatelet agents are common for patients with cardiovascular disease; monitor closely
aspirin + heparin	10	heparin and aspirin both increase anticoagulation. Modify Therapy/Monitor Closely

aspirin + nebivolol	10	aspirin decreases effects of nebivolol by pharmacodynamic antagonism. Use Caution/Monitor. Long term (>1 wk) NSAID use. NSAIDs decrease prostaglandin synthesis
		nebivolol and aspirin both increase serum potassium. Use Caution/Monitor
aspirin + warfarin	10	aspirin increases effects of warfarin by anticoagulation. Modify Therapy/Monitor Closely. Avoid coadministration of chronic high-dose aspirin. Aspirin's antiplatelet properties may increase anticoagulation effect of warfarin. The need for simultaneous use of low-dose aspirin and warfarin is common for patients with cardiovascular disease
atorvastatin + nifedipine	10	nifedipine will decrease the level or effect of atorvastatin by P-glycoprotein (MDR1) efflux transporter. Use Caution/Monitor
		nifedipine will increase the level or effect of atorvastatin by affecting hepatic/intestinal enzyme CYP3A4 metabolism. Use Caution/Monitor
bisoprolol + valsartan	10	bisoprolol, valsartan. Mechanism: pharmacodynamic synergism. Use Caution/Monitor. Risk of fetal compromise if given during pregnancy
		valsartan and bisoprolol both increase serum potassium. Use Caution/Monitor
eplerenone + fosinopril	10	fosinopril, eplerenone. Mechanism: pharmacodynamic synergism. Use Caution/Monitor. Risk of hyperkalemia
formoterol + torsemide	10	formoterol and torsemide both decrease serum potassium. Use Caution/Monitor
indapamide + metoprolol	10	metoprolol increases and indapamide decreases serum potassium. Effect of interaction is not clear, use caution. Use Caution/Monitor
sacubitril/valsartan + spironolactone	10	sacubitril/valsartan and spironolactone both increase serum potassium. Modify Therapy/Monitor Closely
amiodarone + warfarin	9	amiodarone will increase the level or effect of warfarin by affecting hepatic enzyme CYP2C9/10 metabolism. Modify Therapy/Monitor Closely. Coadministration increases INR by 100% after 3-4 days. Reduce warfarin dose by one-third to one-half and monitor INR
apixaban + aspirin	9	aspirin and apixaban both increase anticoagulation. Modify Therapy/Monitor Closely. Both drugs have the potential to cause bleeding. The need for simultaneous use of low-dose aspirin (<100 mg/day) with anticoagulants are common for patients with cardiovascular disease, but may result in increased bleeding; monitor closely. Promptly evaluate any signs or symptoms of blood loss if treated concomitantly with low-dose aspiriin. Avoid coadministration with chronic use of higher dose aspirin. In 1 trial (APPRAISE-2), therapy was terminated because of significantly increased bleeding when apixaban was administered with dual antiplatelet therapy (eg, aspirin plus clopidogrel) compared with single antiplatelet treatment
bisoprolol + ivabradine	9	ivabradine, bisoprolol. Either increases effects of the other by pharmacodynamic synergism. Modify Therapy/Monitor Closely. Most patients receiving ivabradine will also be treated with a beta-blocker. The risk of bradycardia increases with coadministration of drugs that slow heart rate (eg, digoxin, amiodarone, beta-blockers). Monitor heart rate in patients taking ivabradine with other negative chronotropes
		meloxicam decreases effects of bisoprolol by pharmacodynamic antagonism. Use Caution/Monitor. Long term (>1 wk) NSAID use. NSAIDs decrease prostaglandin synthesis
		bisoprolol and meloxicam both increase serum potassium. Use Caution/Monitor

aspirin + enoxaparin	8	enoxaparin and aspirin both increase anticoagulation. Use Caution/Monitor. Additive effects are intended when both drugs are prescribed as indicated for unstable angina, non-Q-wave MI, and STEMI
		aspirin, enoxaparin. Either increases toxicity of the other by pharmacodynamic synergism. Use Caution/Monitor. The need for simultaneous use of low-dose aspirin and anticoagulant or antiplatelet agents are common for patients with cardiovascular disease; monitor closely
atorvastatin + budesonide	8	atorvastatin will increase the level or effect of budesonide by P-glycoprotein (MDR1) efflux transporter. Use Caution/Monitor
bisoprolol + felodipine	8	bisoprolol and felodipine both increase anti-hypertensive channel blocking. Modify Therapy/Monitor Closely
digoxin + furosemide	8	furosemide increases effects of digoxin by pharmacodynamic synergism. Use Caution/Monitor. Hypokalemia increases digoxin effects
		digoxin increases and furosemide decreases serum potassium. Effect of interaction is not clear, use caution. Use Caution/Monitor
digoxin + perindopril	8	perindopril increases levels of digoxin by unspecified interaction mechanism. Use Caution/Monitor
formoterol + spironolactone	8	spironolactone increases and formoterol decreases serum potassium. Effect of interaction is not clear, use caution. Modify Therapy/Monitor Closely
metformin + perindopril	8	perindopril increases toxicity of metformin by unspecified interaction mechanism. Use Caution/Monitor. Increases risk for hypoglycemia and lactic acidosis
omeprazole + warfarin	8	omeprazole will increase the level or effect of warfarin by Other (see comment). Use Caution/Monitor. Warfarin's less potent R-enantiomer is metabolized in part by CYP3A4 (and also CYP1A2 and CYP2C19). Monitor INR more frequently if coadministered with inhibitors of these isoenzymes and adjust warfarin dose if needed
aspirin + formoterol	7	aspirin increases and formoterol decreases serum potassium. Effect of interaction is not clear, use caution. Use Caution/Monitor
captopril + digoxin	7	captopril increases levels of digoxin by unspecified interaction mechanism. Use Caution/Monitor
losartan + warfarin	7	losartan will increase the level or effect of warfarin by affecting hepatic enzyme CYP2C9/10 metabolism. Use Caution/Monitor
spironolactone + valsartan	7	valsartan and spironolactone both increase serum potassium. Modify Therapy/Monitor Closely
torsemide + valsartan	7	valsartan increases and torsemide decreases serum potassium. Effect of interaction is not clear, use caution. Use Caution/Monitor
amiodarone + dabigatran	6	amiodarone will increase the level or effect of dabigatran by P-glycoprotein (MDR1) efflux transporter. Use Caution/Monitor. Atrial fibrillation: Avoid coadministering dabigatran with P-gp inhibitors if CrCl <30 mL/min. DVT/PE treatment: Avoid coadministering dabigatran with P-gp inhibitors if CrCl <50 mL/min
aspirin + chlorthalidone	6	aspirin increases and chlorthalidone decreases serum potassium. Effect of interaction is not clear, use caution. Use Caution/Monitor
aspirin + digoxin	6	aspirin and digoxin both increase serum potassium. Use Caution/Monitor

aspirin + olmesartan	6	olmesartan, aspirin. Either increases toxicity of the other by Other (see comment). Use Caution/Monitor. Comment: May result in renal function deterioration, particularly in elderly or volume depleted individuals
		aspirin decreases effects of olmesartan by pharmacodynamic antagonism. Modify Therapy/Monitor Closely. NSAIDs decrease synthesis of vasodilating renal prostaglandins, and thus affect fluid homeostasis and may diminish antihypertensive effect
		olmesartan and aspirin both increase serum potassium. Use Caution/Monitor
aspirin + ramipril	6	ramipril, aspirin. Either increases toxicity of the other by Other (see comment). Use Caution/Monitor. Comment: May result in renal function deterioration, particularly with high doses of aspirin, in elderly or volume depleted individuals
atorvastatin + ranolazine	6	ranolazine will increase the level or effect of atorvastatin by P-glycoprotein (MDR1) efflux transporter. Use Caution/Monitor
		ranolazine increases toxicity of atorvastatin by Other (see comment). Modify Therapy/Monitor Closely. Comment: OATP1B1 inhibitors may increase risk of myopathy
bisoprolol + lornoxicam	6	lornoxicam decreases effects of bisoprolol by pharmacodynamic antagonism. Use Caution/Monitor. Long term (>1 wk) NSAID use. NSAIDs decrease prostaglandin synthesis
		bisoprolol and lornoxicam both increase serum potassium. Use Caution/Monitor
captopril + furosemide	6	captopril, furosemide. Mechanism: pharmacodynamic synergism. Use Caution/Monitor. Risk of acute hypotension, renal insufficiency
eplerenone + lisinopril	6	lisinopril, eplerenone. Mechanism: pharmacodynamic synergism. Use Caution/Monitor. Risk of hyperkalemia
eplerenone + ramipril	6	ramipril, eplerenone. Mechanism: pharmacodynamic synergism. Use Caution/Monitor. Risk of hyperkalemia
ferrous sulfate + omeprazole	6	omeprazole will decrease the level or effect of ferrous sulfate by increasing gastric pH. Applies only to oral form of both agents. Use Caution/Monitor
hydrochlorothiazide + metoprolol	6	hydrochlorothiazide, metoprolol. Either increases toxicity of the other by Other (see comment). Modify Therapy/Monitor Closely. Comment: May cause idiosyncratic reaction, resulting in acute transient myopia and acute angle-closure glaucoma, which can lead to permanent vision loss
		metoprolol increases and hydrochlorothiazide decreases serum potassium. Effect of interaction is not clear, use caution. Use Caution/Monitor
indapamide + torsemide	6	torsemide and indapamide both decrease serum potassium. Use Caution/Monitor
ivabradine + metoprolol	6	ivabradine, metoprolol. Either increases effects of the other by pharmacodynamic synergism. Modify Therapy/Monitor Closely. Most patients receiving ivabradine will also be treated with a beta-blocker. The risk of bradycardia increases with coadministration of drugs that slow heart rate (eg, digoxin, amiodarone, beta-blockers). Monitor heart rate in patients taking ivabradine with other negative chronotropes
losartan + sotalol	6	sotalol, losartan. Mechanism: pharmacodynamic synergism. Use Caution/Monitor. Risk of fetal compromise if given during pregnancy
		losartan and sotalol both increase serum potassium. Use Caution/Monitor
amiodarone + metformin	5	amiodarone will increase the level or effect of metformin by basic (cationic) drug competition for renal tubular clearance. Use Caution/Monitor

aspirin + dabigatran	5	dabigatran and aspirin both increase anticoagulation. Modify Therapy/Monitor Closely. Both drugs have the potential to cause bleeding. The need for simultaneous use of low-dose aspirin (<100 mg/day) with anticoagulants are common for patients with cardiovascular disease, but may result in increased bleeding; monitor closely. Promptly evaluate any signs or symptoms of blood loss if treated concomitantly with low-dose aspirin. Avoid coadministration with chronic use of higher dose aspirin
candesartan + torsemide	5	candesartan increases and torsemide decreases serum potassium. Effect of interaction is not clear, use caution. Use Caution/Monitor
captopril + metformin	5	captopril increases toxicity of metformin by unspecified interaction mechanism. Use Caution/Monitor. Increases risk for hypoglycemia and lactic acidosis
clopidogrel + warfarin	5	clopidogrel, warfarin. Either increases effects of the other by pharmacodynamic synergism. Modify Therapy/Monitor Closely. Drugs with antiplatelet properties may increase anticoagulation effect of warfarin
digoxin + enalapril	5	enalapril increases levels of digoxin by unspecified interaction mechanism. Use Caution/Monitor
empagliflozin + torsemide	5	empagliflozin, torsemide. Either increases effects of the other by pharmacodynamic synergism. Use Caution/Monitor. Coadministration of empagliflozin with diuretics results in increased urine volume and frequency of voids, which might enhance the potential for volume depletion
formoterol + moxonidine	5	moxonidine increases and formoterol decreases sedation. Effect of interaction is not clear, use caution. Use Caution/Monitor
furosemide + losartan	5	losartan increases and furosemide decreases serum potassium. Effect of interaction is not clear, use caution. Use Caution/Monitor
insulin + losartan	5	losartan increases effects of insulin regular human by unspecified interaction mechanism. Use Caution/Monitor. Concomitant use of insulin and ARBs may require insulin dosage adjustment and increased glucose monitoring
aceclofenac + bisoprolol	4	aceclofenac decreases effects of bisoprolol by pharmacodynamic antagonism. Use Caution/Monitor. Long term (>1 wk) NSAID use. NSAIDs decrease prostaglandin synthesis
		bisoprolol and aceclofenac both increase serum potassium. Use Caution/Monitor
amiodarone + budesonide	4	budesonide will decrease the level or effect of amiodarone by affecting hepatic/intestinal enzyme CYP3A4 metabolism. Use Caution/Monitor
		amiodarone will increase the level or effect of budesonide by P-glycoprotein (MDR1) efflux transporter. Use Caution/Monitor
amlodipine + carvedilol	4	carvedilol and amlodipine both increase anti-hypertensive channel blocking. Modify Therapy/Monitor Closely
aspirin + atenolol	4	aspirin decreases effects of atenolol by pharmacodynamic antagonism. Use Caution/Monitor. Long term (>1 wk) NSAID use. NSAIDs decrease prostaglandin synthesis
aspirin + atenolol	4	atenolol and aspirin both increase serum potassium. Use Caution/Monitor
aspirin + doxazosin	4	aspirin decreases effects of doxazosin by pharmacodynamic antagonism. Use Caution/Monitor. NSAIDs decrease prostaglandin synthesis
aspirin + ketoprofen	4	aspirin and ketoprofen both increase anticoagulation. Use Caution/Monitor
		aspirin and ketoprofen both increase serum potassium. Use Caution/Monitor
aspirin + ketorolac	4	aspirin and ketorolac both increase anticoagulation. Use Caution/Monitor

		aspirin and ketorolac both increase serum potassium. Use Caution/Monitor
aspirin + meloxicam	4	aspirin and meloxicam both increase anticoagulation. Use Caution/Monitor
		aspirin and meloxicam both increase serum potassium. Use Caution/Monitor
aspirin + sotalol	4	sotalol and aspirin both increase serum potassium. Use Caution/Monitor
		aspirin decreases effects of sotalol by pharmacodynamic antagonism. Use Caution/Monitor. Long term (>1 wk) NSAID use. NSAIDs decrease prostaglandin synthesis
atenolol + sacubitril/valsartan	4	atenolol, sacubitril/valsartan. Mechanism: pharmacodynamic synergism. Use Caution/Monitor. Risk of fetal compromise if given during pregnancy
		sacubitril/valsartan and atenolol both increase serum potassium. Use Caution/Monitor
bisoprolol + chlorthalidone	4	bisoprolol increases and chlorthalidone decreases serum potassium. Effect of interaction is not clear, use caution. Use Caution/Monitor
bisoprolol + nifedipine	4	bisoprolol, nifedipine. Either decreases effects of the other by pharmacodynamic synergism. Use Caution/Monitor. Both drugs lower blood pressure
bisoprolol + olmesartan	4	bisoprolol, olmesartan. Mechanism: pharmacodynamic synergism. Use Caution/Monitor. Risk of fetal compromise if given during pregnancy
		olmesartan and bisoprolol both increase serum potassium. Use Caution/Monitor
calcium carbonate + metoprolol	4	calcium carbonate decreases effects of metoprolol by unspecified interaction mechanism. Use Caution/Monitor.
		calcium carbonate decreases levels of metoprolol by inhibition of GI absorption. Applies only to oral form of both agents. Use Caution/Monitor. Separate by 2 hours
celecoxib + meloxicam	4	calcium carbonate decreases effects of metoprolol by unspecified interaction mechanism. Use Caution/Monitor
		calcium carbonate decreases levels of metoprolol by inhibition of GI absorption. Applies only to oral form of both agents. Use Caution/Monitor. Separate by 2 hours
ciprofloxacin + omeprazole	4	omeprazole will decrease the level or effect of ciprofloxacin by unknown mechanism. Use Caution/Monitor. Absorption of the ciprofloxacin ER tablet was slightly diminished (20%) when coadministered with omeprazole
clopidogrel + ticagrelor	4	ticagrelor, clopidogrel. Either increases effects of the other by Other (see comment). Use Caution/Monitor. Comment: Increased risk of bleeding during concomitant use of medications that increase potential for bleeding
digoxin + lisinopril	4	lisinopril increases levels of digoxin by unspecified interaction mechanism. Use Caution/Monitor
digoxin + metformin	4	digoxin, metformin. Either increases levels of the other by basic (cationic) drug competition for renal tubular clearance. Use Caution/Monitor. Measure serum digoxin concentrations before initiating metformin. Monitor patients who take both metformin and digoxin for possible digoxin toxicity and lactic acidosis. Reduce the digoxin and/or metformin dose as necessary
digoxin + omeprazole	4	omeprazole increases toxicity of digoxin by Other (see comment). Use Caution/Monitor. Comment: Prolonged use of PPIs may cause hypomagnesemia and increase risk for digoxin toxicity
enalapril + metformin	4	enalapril increases toxicity of metformin by unspecified interaction mechanism. Use Caution/Monitor. Increases risk for hypoglycemia and lactic acidosis

enoxaparin + losartan	4	enoxaparin increases toxicity of losartan by Other (see comment). Use Caution/Monitor. Comment: Low molecular weight heparins may suppress adrenal aldosterone secretion, which can potentially cause hyperkalemia
eplerenone + losartan	4	losartan, eplerenone. Mechanism: pharmacodynamic synergism. Use Caution/Monitor. Risk of hyperkalemia
felodipine + nitroglycerin	4	felodipine, nitroglycerin sublingual. Either increases toxicity of the other by additive vasodilation. Modify Therapy/Monitor Closely. Marked orthostatic hypotension reported with concomitant use
formoterol + nebivolol	4	nebivolol decreases effects of formoterol by pharmacodynamic antagonism. Use Caution/Monitor
		nebivolol increases and formoterol decreases serum potassium. Effect of interaction is not clear, use caution. Use Caution/Monitor
furosemide + torsemide	4	furosemide and torsemide both decrease serum potassium. Use Caution/Monitor
indapamide + spironolactone	4	spironolactone increases and indapamide decreases serum potassium. Effect of interaction is not clear, use caution. Modify Therapy/Monitor Closely
ketoprofen + meloxicam	4	ketoprofen and meloxicam both increase anticoagulation. Use Caution/Monitor
		ketoprofen and meloxicam both increase serum potassium. Use Caution/Monitor
metoprolol + valsartan	4	metoprolol, valsartan. Mechanism: pharmacodynamic synergism. Use Caution/Monitor. Risk of fetal compromise if given during pregnancy
		valsartan and metoprolol both increase serum potassium. Use Caution/Monitor
sotalol + torsemide	4	sotalol increases and torsemide decreases serum potassium. Effect of interaction is not clear, use caution. Use Caution/Monitor
sotalol + valsartan	4	sotalol, valsartan. Mechanism: pharmacodynamic synergism. Use Caution/Monitor. Risk of fetal compromise if given during pregnancy
		valsartan and sotalol both increase serum potassium. Use Caution/Monitor
amiodarone + carvedilol	3	amiodarone will increase the level or effect of carvedilol by affecting hepatic enzyme CYP2C9/10 metabolism. Use Caution/Monitor
		amiodarone will increase the level or effect of carvedilol by affecting hepatic enzyme CYP2D6 metabolism. Use Caution/Monitor. Monitor for signs of bradycardia or heart block when amiodarone and a beta adrenergic blocker are coadministered. Amiodarone should be used with caution in patients receiving a beta adrenergic blocker, particularly if there is suspicion of underlying dysfunction of the sinus node, such as bradycardia or sick sinus syndrome, or if there is partial AV block
		amiodarone, carvedilol. Mechanism: pharmacodynamic synergism. Use Caution/Monitor. Risk of cardiotoxicity with bradycardia
amlodipine + nifedipine	3	amlodipine and nifedipine both increase anti-hypertensive channel blocking. Use Caution/Monitor
amlodipine + sotalol	3	sotalol and amlodipine both increase anti-hypertensive channel blocking. Modify Therapy/Monitor Closely
aspirin + potassium	3	aspirin and potassium chloride both increase serum potassium. Modify Therapy/Monitor Closely

atorvastatin + carbamazepine	3	carbamazepine increases toxicity of atorvastatin by Other (see comment). Use Caution/Monitor. Comment: OATP1B1 inhibitors may increase risk of myopathy
atorvastatin + telmisartan	3	telmisartan increases toxicity of atorvastatin by Other (see comment). Use Caution/Monitor. Comment: OATP1B1 inhibitors may increase risk of myopathy
bisoprolol + potassium	3	bisoprolol and potassium chloride both increase serum potassium. Modify Therapy/Monitor Closely
budesonide + clopidogrel	3	budesonide will increase the level or effect of clopidogrel by affecting hepatic/intestinal enzyme CYP3A4 metabolism. Use Caution/Monitor. CYP3A4 inducers may increase the metabolism of clopidogrel to its active metabolite. Monitor patients for potential increase in antiplatelet effects when CYP3A4 inducers are used in combination with clopidogrel
budesonide + esomeprazole	3	esomeprazole decreases effects of budesonide by increasing gastric pH. Applies only to oral form of both agents. Modify Therapy/Monitor Closely. Enteric-coated budesonide dissolves at pH >5.5. Also, dissolution of extended-release budesonide tablets is pH dependent. Coadministration with drugs that increase gastric pH may cause these budesonide products to prematurely dissolve, and possibly affect release properties and absorption of the drug in the duodenum
budesonide + verapamil	3	verapamil will increase the level or effect of budesonide by affecting hepatic/intestinal enzyme CYP3A4 metabolism. Use Caution/Monitor
		budesonide will decrease the level or effect of verapamil by affecting hepatic/intestinal enzyme CYP3A4 metabolism. Use Caution/Monitor
		verapamil will increase the level or effect of budesonide by P-glycoprotein (MDR1) efflux transporter. Use Caution/Monitor
candesartan + spironolactone	3	candesartan and spironolactone both increase serum potassium. Modify Therapy/Monitor Closely
captopril + chlorthalidone	3	captopril, chlorthalidone. Either increases toxicity of the other by pharmacodynamic synergism. Use Caution/Monitor. Both drugs lower blood pressure. Increased risk of nephrotoxicity. Monitor blood pressure and renal function
captopril + insulin	3	captopril increases effects of insulin regular human by pharmacodynamic synergism. Use Caution/Monitor. Both drugs decrease blood glucose. Monitor blood glucose
carbamazepine + omeprazole	3	carbamazepine will decrease the level or effect of omeprazole by affecting hepatic/intestinal enzyme CYP3A4 metabolism. Use Caution/Monitor
carvedilol + spironolactone	3	carvedilol and spironolactone both increase serum potassium. Modify Therapy/Monitor Closely
carvedilol + torsemide	3	carvedilol increases and torsemide decreases serum potassium. Effect of interaction is not clear, use caution. Use Caution/Monitor
clopidogrel + dabigatran	3	dabigatran, clopidogrel. Either increases effects of the other by pharmacodynamic synergism. Use Caution/Monitor. Both drugs have the potential to cause bleeding. Concomitant use may increase risk of bleeding
clopidogrel + felodipine	3	felodipine decreases effects of clopidogrel by decreasing metabolism. Use Caution/Monitor. Cytochrome P450 2C19 inhibitors decrease the conversion of clopidogrel to its active form
digoxin + formoterol	3	digoxin increases and formoterol decreases serum potassium. Effect of interaction is not clear, use caution. Use Caution/Monitor

digoxin + pantoprazole	3	pantoprazole increases toxicity of digoxin by Other (see comment). Use Caution/Monitor. Comment: Prolonged use of PPIs may cause hypomagnesemia and increase risk for digoxin toxicity
doxazosin + metoprolol	3	doxazosin and metoprolol both increase anti-hypertensive channel blocking. Modify Therapy/Monitor Closely
enalapril + enoxaparin	3	enoxaparin increases toxicity of enalapril by Other (see comment). Use Caution/Monitor. Comment: Low molecular weight heparins may suppress adrenal aldosterone secretion, which can potentially cause hyperkalemia
enalapril + furosemide	3	enalapril, furosemide. Mechanism: pharmacodynamic synergism. Use Caution/Monitor. Risk of acute hypotension, renal insufficiency
enalapril + insulin	3	enalapril increases effects of insulin regular human by pharmacodynamic synergism. Use Caution/Monitor
formoterol + salmeterol	3	formoterol and salmeterol both decrease serum potassium. Use Caution/Monitor
		formoterol and salmeterol both decrease sedation. Use Caution/Monitor
		formoterol and salmeterol both increase sympathetic (adrenergic) effects, including increased blood pressure and heart rate. Use Caution/Monitor.
fosinopril + metformin	3	fosinopril increases toxicity of metformin by unspecified interaction mechanism. Use Caution/Monitor. Increases risk for hypoglycemia and lactic acidosis
hydrochlorothiazide + telmisartan	3	telmisartan increases and hydrochlorothiazide decreases serum potassium. Effect of interaction is not clear, use caution. Use Caution/Monitor
indomethacin + losartan	3	losartan, indomethacin. Either increases toxicity of the other by Other (see comment). Use Caution/Monitor. Comment: May result in renal function deterioration, particularly in elderly or volume depleted individuals
		indomethacin decreases effects of losartan by pharmacodynamic antagonism. Modify Therapy/Monitor Closely. NSAIDs decrease synthesis of vasodilating renal prostaglandins, and thus affect fluid homeostasis and may diminish antihypertensive effect
		losartan and indomethacin both increase serum potassium. Use Caution/Monitor
insulin + lisinopril	3	lisinopril increases effects of insulin regular human by pharmacodynamic synergism. Use Caution/Monitor
metoprolol + nifedipine	3	metoprolol and nifedipine both increase anti-hypertensive channel blocking. Modify Therapy/Monitor Closely
aceclofenac + aspirin	2	aceclofenac and aspirin both increase anticoagulation. Use Caution/Monitor
		aceclofenac and aspirin both increase serum potassium. Use Caution/Monitor
acetazolamide + aspirin	2	acetazolamide, aspirin. Either increases levels of the other by Other (see comment). Use Caution/Monitor. Comment: Carbonic anhydrase inhibitors (CAIs) and salicylates inhibit each other's renal tubular secretion, resulting in increased plasma levels. CAIs also shift salicylates from plasma to the CNS, leading to potential neurotoxicity
		acetazolamide, aspirin. Mechanism: passive renal tubular reabsorption due to increased pH. Use Caution/Monitor. Salicylate levels increased at moderate doses; risk of CNS toxicity. Salicylate levels decreased at large doses (d/t increased renal excretion of unchanged salicylic acid)

aluminum hydroxide + bisoprolol	2	aluminum hydroxide decreases levels of bisoprolol by inhibition of GI absorption. Applies only to oral form of both agents. Use Caution/Monitor. Separate by 2 hours
amiodarone + atenolol	2	amiodarone, atenolol. Mechanism: pharmacodynamic synergism. Use Caution/Monitor. Risk of cardiotoxicity with bradycardia
amiodarone + formoterol	2	amiodarone and formoterol both increase QTc interval. Use Caution/Monitor
amiodarone + nebivolol	2	amiodarone will increase the level or effect of nebivolol by affecting hepatic enzyme CYP2D6 metabolism. Use Caution/Monitor. Monitor cardiac function carefully and observe for signs of bradycardia or heart block when amiodarone and a beta adrenergic blocker are coadministered. Amiodarone should be used with caution in patients receiving a beta adrenergic blocker, particularly if there is suspicion of underlying dysfunction of the sinus node, such as bradycardia or sick sinus syndrome, or if there is partial AV block
		amiodarone, nebivolol. Mechanism: pharmacodynamic synergism. Use Caution/Monitor. Risk of cardiotoxicity with bradycardia
amlodipine + nebivolol	2	nebivolol, amlodipine. Either increases effects of the other by pharmacodynamic synergism. Use Caution/Monitor. Both drugs lower blood pressure
aspirin + celecoxib	2	aspirin and celecoxib both increase anticoagulation. Use Caution/Monitor
		aspirin and celecoxib both increase serum potassium. Use Caution/Monitor
aspirin + fondaparinux	2	fondaparinux and aspirin both increase anticoagulation. Modify Therapy/Monitor Closely
aspirin + ibuprofen	2	aspirin and ibuprofen both increase anticoagulation. Use Caution/Monitor
		aspirin and ibuprofen both increase serum potassium. Use Caution/Monitor
aspirin + lornoxicam	2	aspirin and lornoxicam both increase anticoagulation. Use Caution/Monitor
		aspirin and lornoxicam both increase serum potassium. Use Caution/Monitor
aspirin + timolol	2	aspirin decreases effects of timolol by pharmacodynamic antagonism. Use Caution/Monitor. Long term (>1 wk) NSAID use. NSAIDs decrease prostaglandin synthesis
		timolol and aspirin both increase serum potassium. Use Caution/Monitor
atenolol + torsemide	2	atenolol increases and torsemide decreases serum potassium. Effect of interaction is not clear, use caution. Use Caution/Monitor
azilsartan + meloxicam	2	meloxicam, azilsartan. Either increases toxicity of the other by Other (see comment). Use Caution/Monitor. Comment: May result in renal function deterioration, particularly in elderly or volume depleted individuals
		meloxicam decreases effects of azilsartan by pharmacodynamic antagonism. Modify Therapy/Monitor Closely. NSAIDs decrease synthesis of vasodilating renal prostaglandins, and thus affect fluid homeostasis and may diminish antihypertensive effect
azithromycin + cetirizine	2	azithromycin will increase the level or effect of cetirizine by P-glycoprotein (MDR1) efflux transporter. Use Caution/Monitor
bisoprolol + calcium carbonate	2	calcium carbonate decreases effects of bisoprolol by unspecified interaction mechanism. Use Caution/Monitor
		calcium carbonate decreases levels of bisoprolol by inhibition of GI absorption. Applies only to oral form of both agents. Use Caution/Monitor. Separate by 2 hours
bisoprolol + celecoxib	2	celecoxib decreases effects of bisoprolol by pharmacodynamic antagonism. Use Caution/Monitor. Long term (>1 wk) NSAID use. NSAIDs decrease prostaglandin synthesis
		bisoprolol and celecoxib both increase serum potassium. Use Caution/Monitor

bisoprolol + doxazosin	2	doxazosin and bisoprolol both increase anti-hypertensive channel blocking. Modify Therapy/Monitor Closely
bisoprolol + ketoprofen	2	ketoprofen decreases effects of bisoprolol by pharmacodynamic antagonism. Use Caution/Monitor. Long term (>1 wk) NSAID use. NSAIDs decrease prostaglandin synthesis
		bisoprolol and ketoprofen both increase serum potassium. Use Caution/Monitor
bisoprolol + ketorolac	2	ketorolac decreases effects of bisoprolol by pharmacodynamic antagonism. Use Caution/Monitor. Long term (>1 wk) NSAID use. NSAIDs decrease prostaglandin synthesis
		bisoprolol and ketorolac both increase serum potassium. Use Caution/Monitor
budesonide + omeprazole	2	omeprazole decreases effects of budesonide by increasing gastric pH. Applies only to oral form of both agents. Modify Therapy/Monitor Closely. Enteric-coated budesonide dissolves at pH >5.5. Also, dissolution of extended-release budesonide tablets is pH dependent. Coadministration with drugs that increase gastric pH may cause these budesonide products to prematurely dissolve, and possibly affect release properties and absorption of the drug in the duodenum
calcium carbonate + captopril	2	calcium carbonate decreases effects of captopril by unspecified interaction mechanism. Use Caution/Monitor. Calcium carbonate may decrease absorption of captopril
calcium carbonate + nifedipine	2	calcium carbonate decreases effects of nifedipine by pharmacodynamic antagonism. Use Caution/Monitor
calcium carbonate + vitamin D	2	calcium carbonate decreases effects of nifedipine by pharmacodynamic antagonism. Use Caution/Monitor
candesartan + metoprolol	2	metoprolol, candesartan. Mechanism: pharmacodynamic synergism. Use Caution/Monitor. Risk of fetal compromise if given during pregnancy
		candesartan and metoprolol both increase serum potassium. Use Caution/Monitor
captopril + doxazosin	2	captopril, doxazosin. Either increases effects of the other by Mechanism: pharmacodynamic synergism. Use Caution/Monitor. Exaggerated first dose hypotensive response. Both drugs lower blood pressure. Monitor blood pressure
captopril + hydrochlorothiazide	2	captopril, hydrochlorothiazide. Either increases effects of the other by pharmacodynamic synergism. Use Caution/Monitor. Both drugs lower blood pressure. Increased risk of nephrotoxicity. Monitor blood pressure and renal function
captopril + ketoprofen	2	captopril, ketoprofen. Either increases toxicity of the other by Other (see comment). Use Caution/Monitor. Comment: May result in renal function deterioration, particularly in elderly or volume depleted individuals
captopril + potassium	2	captopril increases levels of potassium chloride by decreasing elimination. Use Caution/Monitor. Risk of hyperkalemia
		potassium chloride increases toxicity of captopril by Mechanism: unspecified interaction mechanism. Modify Therapy/Monitor Closely. Both drugs increase potassium. Monitor potassium
carvedilol + losartan	2	carvedilol, losartan. Mechanism: pharmacodynamic synergism. Use Caution/Monitor. Risk of fetal compromise if given during pregnancy
		losartan and carvedilol both increase serum potassium. Use Caution/Monitor
carvedilol + telmisartan	2	carvedilol, telmisartan. Mechanism: pharmacodynamic synergism. Use Caution/Monitor. Risk of fetal compromise if given during pregnancy

		telmisartan and carvedilol both increase serum potassium. Use Caution/Monitor
celecoxib + lisinopril	2	lisinopril, celecoxib. Either increases toxicity of the other by Other (see comment). Use Caution/Monitor. Comment: May result in renal function deterioration, particularly in elderly or volume depleted individuals
chlorthalidone + metoprolol	2	metoprolol increases and chlorthalidone decreases serum potassium. Effect of interaction is not clear, use caution. Use Caution/Monitor
ciprofloxacin + dexamethasone	2	dexamethasone and ciprofloxacin both increase Other (see comment). Use Caution/Monitor. Coadministration of quinolone antibiotics and corticosteroids may increase risk of tendon rupture
clopidogrel + enoxaparin	2	enoxaparin, clopidogrel. Either increases effects of the other by pharmacodynamic synergism. Modify Therapy/Monitor Closely. Enhanced risk of hemorrhage; additive effects are intended when both drugs are prescribed as indicated for ACS
clopidogrel + heparin	2	heparin, clopidogrel. Either increases effects of the other by pharmacodynamic synergism. Modify Therapy/Monitor Closely. Enhanced risk of hemorrhage; additive effects are intended when both drugs are prescribed as indicated for ACS
dexamethasone + loratadine	2	dexamethasone will decrease the level or effect of loratadine by affecting hepatic/intestinal enzyme CYP3A4 metabolism. Use Caution/Monitor
		loratadine will increase the level or effect of dexamethasone by P-glycoprotein (MDR1) efflux transporter. Use Caution/Monitor
diclofenac + lornoxicam	2	diclofenac and lornoxicam both increase anticoagulation. Use Caution/Monitor
		diclofenac and lornoxicam both increase serum potassium. Use Caution/Monitor
digoxin + esomeprazole	2	esomeprazole increases toxicity of digoxin by Other (see comment). Use Caution/Monitor. Comment: Prolonged use of PPIs may cause hypomagnesemia and increase risk for digoxin toxicity
digoxin + indapamide	2	indapamide increases effects of digoxin by pharmacodynamic synergism. Use Caution/Monitor. Hypokalemia increases digoxin effects
		digoxin increases and indapamide decreases serum potassium. Effect of interaction is not clear, use caution. Use Caution/Monitor
digoxin + nebivolol	2	nebivolol increases effects of digoxin by pharmacodynamic synergism. Use Caution/Monitor. Enhanced bradycardia
		nebivolol and digoxin both increase serum potassium. Use Caution/Monitor
digoxin + verapamil	2	verapamil will increase the level or effect of digoxin by P-glycoprotein (MDR1) efflux transporter. Use Caution/Monitor. Toxicity characterized by gastrointestinal and neuropsychiatric symptoms, and cardiac arrhythmias may result
		digoxin will increase the level or effect of verapamil by basic (cationic) drug competition for renal tubular clearance. Use Caution/Monitor
doxazosin + nifedipine	2	doxazosin and nifedipine both increase anti-hypertensive channel blocking. Use Caution/Monitor
enalapril + potassium	2	enalapril increases levels of potassium chloride by decreasing elimination. Use Caution/Monitor. Risk of hyperkalemia
felodipine + metoprolol	2	metoprolol and felodipine both increase anti-hypertensive channel blocking. Modify Therapy/Monitor Closely

formoterol + furosemide	2	formoterol and furosemide both decrease serum potassium. Use Caution/Monitor
furosemide + lisinopril	2	lisinopril, furosemide. Mechanism: pharmacodynamic synergism. Use Caution/Monitor. Risk of acute hypotension, renal insufficiency
furosemide + potassium	2	potassium chloride increases and furosemide decreases serum potassium. Effect of interaction is not clear, use caution. Modify Therapy/Monitor Closely
heparin + meloxicam	2	heparin and meloxicam both increase anticoagulation. Modify Therapy/Monitor Closely
heparin + rivaroxaban	2	rivaroxaban, heparin. Either increases effects of the other by anticoagulation. Use Caution/Monitor. Avoid concurrent use of rivaroxaban with other anticoagulants due to increased bleeding risk other than during therapeutic transition periods where patients should be observed closely. Monitor for signs/symptoms of blood loss
hydrochlorothiazide + losartan	2	losartan increases and hydrochlorothiazide decreases serum potassium. Effect of interaction is not clear, use caution. Use Caution/Monitor
hydrochlorothiazide + torsemide	2	torsemide and hydrochlorothiazide both decrease serum potassium. Use Caution/Monitor
indapamide + sotalol	2	sotalol increases and indapamide decreases serum potassium. Effect of interaction is not clear, use caution. Use Caution/Monitor
indapamide + valsartan	2	valsartan increases and indapamide decreases serum potassium. Effect of interaction is not clear, use caution. Use Caution/Monitor
insulin + torsemide	2	torsemide decreases effects of insulin degludec by Other (see comment). Use Caution/Monitor. Comment: Diuretics may cause hyperglycemia and glycosuria in patients with diabetes mellitus, possibly by diuretic-induced hpokalemia
insulin + valsartan	2	valsartan increases effects of insulin aspart by unspecified interaction mechanism. Use Caution/Monitor. Concomitant use of insulin and ARBs may require insulin dosage adjustment and increased glucose monitoring
ketorolac + lisinopril	2	lisinopril, ketorolac. Either increases toxicity of the other by Other (see comment). Use Caution/Monitor. Comment: May result in renal function deterioration, particularly in elderly or volume depleted individuals
ketorolac + meloxicam	2	ketorolac and meloxicam both increase anticoagulation. Use Caution/Monitor
		ketorolac and meloxicam both increase serum potassium. Use Caution/Monitor
levothyroxine + metformin	2	levothyroxine decreases effects of metformin by pharmacodynamic antagonism. Use Caution/Monitor. Patient should be closely observed for loss of blood glucose control; when drugs are withdrawn from a patient receiving metformin, patient should be observed closely for hypoglycemia
levothyroxine + warfarin	2	levothyroxine increases effects of warfarin by unspecified interaction mechanism. Use Caution/Monitor
lisinopril + meloxicam	2	lisinopril, meloxicam. Either increases toxicity of the other by Other (see comment). Use Caution/Monitor. Comment: May result in renal function deterioration, particularly in elderly or volume depleted individuals
losartan + nebivolol	2	nebivolol, losartan. Mechanism: pharmacodynamic synergism. Use Caution/Monitor. Risk of fetal compromise if given during pregnancy
		losartan and nebivolol both increase serum potassium. Use Caution/Monitor
losartan + potassium	2	losartan and potassium chloride both increase serum potassium. Use Caution/Monitor

metoprolol + ranolazine	2	ranolazine will increase the level or effect of metoprolol by affecting hepatic enzyme CYP2D6 metabolism. Use Caution/Monitor
metoprolol + telmisartan	2	metoprolol, telmisartan. Mechanism: pharmacodynamic synergism. Use Caution/Monitor. Risk of fetal compromise if given during pregnancy
		telmisartan and metoprolol both increase serum potassium. Use Caution/Monitor
nebivolol + spironolactone	2	nebivolol and spironolactone both increase serum potassium. Modify Therapy/Monitor Closely
nebivolol + torsemide	2	nebivolol increases and torsemide decreases serum potassium. Effect of interaction is not clear, use caution. Use Caution/Monitor
nifedipine + nitroglycerin	2	nifedipine, nitroglycerin sublingual. Either increases toxicity of the other by additive vasodilation. Modify Therapy/Monitor Closely. Marked orthostatic hypotension reported with concomitant use
phenylephrine + xylometazoline	2	phenylephrine and xylometazoline both decrease sedation. Use Caution/Monitor
		phenylephrine and xylometazoline both increase sympathetic (adrenergic) effects, including increased blood pressure and heart rate. Use Caution/Monitor
ramipril + torsemide	2	ramipril, torsemide. Mechanism: pharmacodynamic synergism. Use Caution/Monitor. Risk of acute hypotension, renal insufficiency
sacubitril/valsartan + sotalol	2	sotalol, sacubitril/valsartan. Mechanism: pharmacodynamic synergism. Use Caution/Monitor. Risk of fetal compromise if given during pregnancy
		sacubitril/valsartan and sotalol both increase serum potassium. Use Caution/Monitor
sotalol + spironolactone	2	sotalol and spironolactone both increase serum potassium. Modify Therapy/Monitor Closely
sotalol + telmisartan	2	sotalol, telmisartan. Mechanism: pharmacodynamic synergism. Use Caution/Monitor. Risk of fetal compromise if given during pregnancy
		telmisartan and sotalol both increase serum potassium. Use Caution/Monitor
telmisartan + torsemide	2	telmisartan increases and torsemide decreases serum potassium. Effect of interaction is not clear, use caution. Use Caution/Monitor
aceclofenac + hydrochlorothiazide	1	aceclofenac increases and hydrochlorothiazide decreases serum potassium. Effect of interaction is not clear, use caution. Use Caution/Monitor
aceclofenac + indapamide	1	aceclofenac increases and indapamide decreases serum potassium. Effect of interaction is not clear, use caution. Use Caution/Monitor
allopurinol + calcium carbonate	1	calcium carbonate decreases levels of allopurinol by inhibition of GI absorption. Applies only to oral form of both agents. Use Caution/Monitor. Separate by 2 hours
amiodarone + hydrochlorothiazide	1	amiodarone will increase the level or effect of hydrochlorothiazide by basic (cationic) drug competition for renal tubular clearance. Use Caution/Monitor
amitriptyline + atorvastatin	1	atorvastatin will increase the level or effect of amitriptyline by P-glycoprotein (MDR1) efflux transporter. Use Caution/Monitor
amitriptyline + carbamazepine	1	carbamazepine will decrease the level or effect of amitriptyline by affecting hepatic/intestinal enzyme CYP3A4 metabolism. Use Caution/Monitor
amitriptyline + gabapentin	1	gabapentin, amitriptyline. Either increases effects of the other by pharmacodynamic synergism. Modify Therapy/Monitor Closely. Coadministration of CNS depressants can result in serious, life-threatening, and fatal respiratory depression. Use lowest dose possible and monitor for respiratory depression and sedation

amlodipine + doxazosin	1	doxazosin and amlodipine both increase anti-hypertensive channel blocking. Use Caution/Monitor
amlodipine + magnesium supplement	1	magnesium supplement, amlodipine. Either increases toxicity of the other by pharmacodynamic synergism. Use Caution/Monitor. Calcium channel blockers may increase toxic effects of magnesium; magnesium may increase hypotensive effects of calcium channel blockers
amoxicillin + hydrochlorothiazide	1	amoxicillin, hydrochlorothiazide. Either increases levels of the other by decreasing renal clearance. Use Caution/Monitor
aspirin + cilostazol	1	aspirin, cilostazol. Either increases toxicity of the other by pharmacodynamic synergism. Use Caution/Monitor. The need for simultaneous use of low-dose aspirin and anticoagulant or antiplatelet agents are common for patients with cardiovascular disease; monitor closely
aspirin + escitalopram	1	escitalopram, aspirin. Either increases toxicity of the other by pharmacodynamic synergism. Use Caution/Monitor. Increased risk of upper GI bleeding. SSRIs inhib. serotonin uptake by platelets
aspirin + omega 3 fatty acids	1	omega 3 fatty acids, aspirin. Other (see comment). Use Caution/Monitor. Comment: Patients taking omega-3-fatty acids and an anticoagulant or other drug affecting coagulation should be monitored periodically due to potential increased risk of bleeding
aspirin + prasugrel	1	aspirin, prasugrel. Either increases toxicity of the other by pharmacodynamic synergism. Use Caution/Monitor. The need for simultaneous use of low-dose aspirin and anticoagulant or antiplatelet agents are common for patients with cardiovascular disease; monitor closely
atenolol + chlorthalidone	1	atenolol increases and chlorthalidone decreases serum potassium. Effect of interaction is not clear, use caution. Use Caution/Monitor
atenolol + spironolactone	1	atenolol and spironolactone both increase serum potassium. Modify Therapy/Monitor Closely
atorvastatin + azithromycin	1	azithromycin will increase the level or effect of atorvastatin by affecting hepatic/intestinal enzyme CYP3A4 metabolism. Use Caution/Monitor. If this combination is used, closely monitor for evidence of atorvastatin toxicity (eg, muscle aches or pains, renal dysfunction)
atorvastatin + tinidazole	1	atorvastatin will increase the level or effect of tinidazole by affecting hepatic/intestinal enzyme CYP3A4 metabolism. Use Caution/Monitor
azithromycin + warfarin	1	azithromycin increases toxicity of warfarin by anticoagulation. Use Caution/Monitor. Postmarketing reports have suggested that concomitant administration of azithromycin may potentiate effects of oral warfarin but the interaction does not appear to alter prothrombin time
bisoprolol + timolol	1	bisoprolol and timolol both increase serum potassium. Use Caution/Monitor
bisoprolol + verapamil	1	bisoprolol and verapamil both increase anti-hypertensive channel blocking. Modify Therapy/Monitor Closely
budesonide + clarithromycin	1	clarithromycin will increase the level or effect of budesonide by P-glycoprotein (MDR1) efflux transporter. Use Caution/Monitor
calcium carbonate + levothyroxine	1	calcium carbonate decreases levels of levothyroxine by inhibition of GI absorption. Applies only to oral form of both agents. Use Caution/Monitor. Separate administration by 4 hours
calcium carbonate + ramipril	1	calcium carbonate decreases effects of ramipril by unspecified interaction mechanism. Use Caution/Monitor
calcium carbonate + rosuvastatin	1	calcium carbonate decreases levels of rosuvastatin by inhibition of GI absorption. Applies only to oral form of both agents. Use Caution/Monitor. Separate by 2 hours

candesartan + digoxin	1	candesartan and digoxin both increase serum potassium. Use Caution/Monitor
candesartan + eplerenone	1	candesartan, eplerenone. Mechanism: pharmacodynamic synergism. Use Caution/Monitor. Risk of hyperkalemia
candesartan + indapamide	1	candesartan increases and indapamide decreases serum potassium. Effect of interaction is not clear, use caution. Use Caution/Monitor
candesartan + insulin	1	candesartan increases effects of insulin regular human by unspecified interaction mechanism. Use Caution/Monitor. Concomitant use of insulin and ARBs may require insulin dosage adjustment and increased glucose monitoring
captopril + ibuprofen	1	captopril, ibuprofen. Either increases toxicity of the other by Other (see comment). Use Caution/Monitor. Comment: May result in renal function deterioration, particularly in elderly or volume depleted individuals
captopril + isosorbide dinitrate	1	isosorbide dinitrate, captopril. Either increases effects of the other by pharmacodynamic synergism. Use Caution/Monitor. Both drugs lower blood pressure. Monitor blood pressure
captopril + isosorbide mononitrate	1	isosorbide mononitrate, captopril. Either increases effects of the other by pharmacodynamic synergism. Use Caution/Monitor. Both drugs lower blood pressure. Monitor blood pressure
captopril + linagliptin	1	linagliptin increases toxicity of captopril by Mechanism: unspecified interaction mechanism. Use Caution/Monitor. Increased adverse/toxic effects, specifically, increased risk of angioedema
captopril + meloxicam	1	captopril, meloxicam. Either increases toxicity of the other by Other (see comment). Use Caution/Monitor. Comment: May result in renal function deterioration, particularly in elderly or volume depleted individuals
captopril + naproxen	1	captopril, naproxen. Either increases toxicity of the other by Other (see comment). Use Caution/Monitor. Comment: May result in renal function deterioration, particularly in elderly or volume depleted individuals
captopril + pentoxifylline	1	pentoxifylline, captopril. Either increases effects of the other by pharmacodynamic synergism. Use Caution/Monitor. Both drugs lower blood pressure. Monitor blood pressure
carbamazepine + enalapril	1	enalapril increases levels of carbamazepine by decreasing metabolism. Use Caution/Monitor
carbamazepine + eplerenone	1	carbamazepine will decrease the level or effect of eplerenone by affecting hepatic/intestinal enzyme CYP3A4 metabolism. Use Caution/Monitor
carbamazepine + felodipine	1	felodipine increases levels of carbamazepine by decreasing metabolism. Use Caution/Monitor
carbamazepine + oxcarbazepine	1	oxcarbazepine will decrease the level or effect of carbamazepine by affecting hepatic/intestinal enzyme CYP3A4 metabolism. Use Caution/Monitor
carbamazepine + pantoprazole	1	carbamazepine will decrease the level or effect of pantoprazole by affecting hepatic/intestinal enzyme CYP3A4 metabolism. Use Caution/Monitor
carbamazepine + sacubitril/valsartan	1	carbamazepine will increase the level or effect of sacubitril/valsartan by Other (see comment). Use Caution/Monitor. The results from an in vitro study with human liver tissue indicate that valsartan is a substrate of the hepatic uptake transporter OATP1B1; coadministration with OATP1B1 inhibitors may increase valsartan systemic exposure
carvedilol + hydrochlorothiazide	1	carvedilol increases and hydrochlorothiazide decreases serum potassium. Effect of interaction is not clear, use caution. Use Caution/Monitor

carvedilol + indapamide	1	carvedilol increases and indapamide decreases serum potassium. Effect of interaction is not clear, use caution. Use Caution/Monitor
carvedilol + nifedipine	1	carvedilol and nifedipine both increase anti-hypertensive channel blocking. Modify Therapy/Monitor Closely
ceftriaxone + warfarin	1	ceftriaxone increases effects of warfarin by unspecified interaction mechanism. Use Caution/Monitor
celecoxib + heparin	1	heparin and celecoxib both increase anticoagulation. Modify Therapy/Monitor Closely
celecoxib + indapamide	1	celecoxib increases and indapamide decreases serum potassium. Effect of interaction is not clear, use caution. Use Caution/Monitor
celecoxib + rivaroxaban	1	rivaroxaban, celecoxib. Other (see comment). Use Caution/Monitor. Comment: NSAIDs are known to increase bleeding. Bleeding risk may be increased when NSAIDs are used concomitantly with rivaroxaban. Monitor for signs/symptoms of blood loss
chlorthalidone + meloxicam	1	meloxicam increases and chlorthalidone decreases serum potassium. Effect of interaction is not clear, use caution. Use Caution/Monitor
chlorthalidone + spironolactone	1	spironolactone increases and chlorthalidone decreases serum potassium. Effect of interaction is not clear, use caution. Modify Therapy/Monitor Closely
ciprofloxacin + ibuprofen	1	ibuprofen, ciprofloxacin. Other (see comment). Modify Therapy/Monitor Closely. Comment: Mechanism: unknown. Increased risk of CNS stimulation and seizures with high doses of fluoroquinolones
ciprofloxacin + tinidazole	1	ciprofloxacin will increase the level or effect of tinidazole by affecting hepatic/intestinal enzyme CYP3A4 metabolism. Use Caution/Monitor
clarithromycin + dexamethasone	1	clarithromycin will increase the level or effect of dexamethasone by P-glycoprotein (MDR1) efflux transporter. Use Caution/Monitor
clarithromycin + warfarin	1	clarithromycin will increase the level or effect of warfarin by Other (see comment). Use Caution/Monitor. Warfarin's less potent R-enantiomer is metabolized in part by CYP3A4 (and also CYP1A2 and CYP2C19). Monitor INR more frequently if coadministered with inhibitors of these isoenzymes and adjust warfarin dose if needed
clopidogrel + omega 3 fatty acids	1	omega 3 fatty acids, clopidogrel. Other (see comment). Use Caution/Monitor. Comment: Patients taking omega-3-fatty acids and an anticoagulant or other drug affecting coagulation should be monitored periodically due to potential increased risk of bleeding
dabigatran + diclofenac	1	dabigatran and diclofenac both increase anticoagulation. Use Caution/Monitor. Caution is advised, both drugs have the potential to cause bleeding. Concomitant use may increase risk of bleeding
dabigatran + rivaroxaban	1	rivaroxaban, dabigatran. Either increases effects of the other by anticoagulation. Use Caution/Monitor. Avoid concurrent use of rivaroxaban with other anticoagulants due to increased bleeding risk other than during therapeutic transition periods where patients should be observed closely. Monitor for signs/symptoms of blood loss
dapagliflozin + dulaglutide	1	dulaglutide, dapagliflozin. Either increases effects of the other by pharmacodynamic synergism. Use Caution/Monitor. Antidiabetic agents are often used in combination; dosage adjustments may be required when initiating or discontinuing antidiabetic agents

dapagliflozin + glimepiride	1	glimepiride, dapagliflozin. Either increases effects of the other by pharmacodynamic synergism. Modify Therapy/Monitor Closely. Consider a lower dose of insulin or insulin secretagogue to avoid hypoglycemia when coadministered with dapagliflozin
dexamethasone + enoxaparin	1	dexamethasone, enoxaparin. Other (see comment). Use Caution/Monitor. Comment: Corticosteroids may decrease anticoagulant effects by increasing blood coagulability; conversely, they may impair vascular integrity, thus increasing bleeding risk. Monitor INR closely
dexamethasone + ketorolac	1	ketorolac, dexamethasone. Either increases toxicity of the other by pharmacodynamic synergism. Use Caution/Monitor. Increased risk of GI ulceration
dexamethasone + levofloxacin	1	dexamethasone and levofloxacin both increase Other (see comment). Use Caution/Monitor. Coadministration of quinolone antibiotics and corticosteroids may increase risk of tendon rupture
diclofenac + heparin	1	heparin and diclofenac both increase anticoagulation. Modify Therapy/Monitor Closely
diclofenac + hydrocortisone	1	diclofenac, hydrocortisone. Either increases toxicity of the other by pharmacodynamic synergism. Use Caution/Monitor. Increased risk of GI ulceration
diclofenac + levofloxacin	1	levofloxacin, diclofenac. Other (see comment). Modify Therapy/Monitor Closely. Comment: Risk of CNS stimulation/seizure. Mechanism: Displacement of GABA from receptors in brain
digoxin + felodipine	1	felodipine will increase the level or effect of digoxin by P-glycoprotein (MDR1) efflux transporter. Use Caution/Monitor
digoxin + meloxicam	1	meloxicam and digoxin both increase serum potassium. Use Caution/Monitor
digoxin + memantine	1	digoxin will increase the level or effect of memantine by basic (cationic) drug competition for renal tubular clearance. Use Caution/Monitor
digoxin + sacubitril/valsartan	1	sacubitril/valsartan and digoxin both increase serum potassium. Use Caution/Monitor
digoxin + valsartan	1	valsartan will increase the level or effect of digoxin by decreasing renal clearance. Use Caution/Monitor. Monitor digoxin levels closely when coadministered with drugs that may decrease glomerular filtration or tubular secretion
diltiazem + magnesium supplement	1	magnesium supplement, diltiazem. Either increases toxicity of the other by pharmacodynamic synergism. Use Caution/Monitor. Calcium channel blockers may increase toxic effects of magnesium; magnesium may increase hypotensive effects of calcium channel blockers
doxazosin + fosinopril	1	fosinopril, doxazosin. Mechanism: pharmacodynamic synergism. Use Caution/Monitor. Exaggerated first dose hypotensive response
doxazosin + lisinopril	1	lisinopril, doxazosin. Mechanism: pharmacodynamic synergism. Use Caution/Monitor. Exaggerated first dose hypotensive response
doxazosin + verapamil	1	doxazosin and verapamil both increase anti-hypertensive channel blocking. Use Caution/Monitor
dulaglutide + metformin	1	dulaglutide, metformin. Either increases effects of the other by pharmacodynamic synergism. Use Caution/Monitor. Antidiabetic agents are often used in combination; dosage adjustments may be required when initiating or discontinuing antidiabetic agents
empagliflozin + insulin	1	empagliflozin, insulin regular human. Either increases effects of the other by pharmacodynamic synergism. Modify Therapy/Monitor Closely. Consider a lower dose of

		insulin or insulin secretagogue to avoid hypoglycemia when coadministered with SGLT2 inhibitors
empagliflozin + spironolactone	1	empagliflozin, spironolactone. Either increases effects of the other by pharmacodynamic synergism. Use Caution/Monitor. Coadministration of empagliflozin with diuretics results in increased urine volume and frequency of voids, which might enhance the potential for volume depletion
enalapril + meloxicam	1	enalapril, meloxicam. Either increases toxicity of the other by Other (see comment). Use Caution/Monitor. Comment: May result in renal function deterioration, particularly in elderly or volume depleted individuals
enoxaparin + spironolactone	1	spironolactone, enoxaparin. Either increases toxicity of the other by serum potassium. Use Caution/Monitor. Both drugs may increase serum potassium levels
eplerenone + telmisartan	1	telmisartan, eplerenone. Mechanism: pharmacodynamic synergism. Use Caution/Monitor. Risk of hyperkalemia
eplerenone + valsartan	1	valsartan, eplerenone. Mechanism: pharmacodynamic synergism. Use Caution/Monitor. Risk of hyperkalemia
eplerenone + verapamil	1	verapamil will increase the level or effect of eplerenone by affecting hepatic/intestinal enzyme CYP3A4 metabolism. Use Caution/Monitor. Plasma concentrations and pharmacologic or toxic effects of eplerenone may be increased by verapamil
escitalopram + metformin	1	escitalopram increases effects of metformin by pharmacodynamic synergism. Use Caution/Monitor
escitalopram + omeprazole	1	omeprazole will increase the level or effect of escitalopram by affecting hepatic enzyme CYP2C19 metabolism. Use Caution/Monitor
felodipine + metformin	1	felodipine decreases effects of metformin by pharmacodynamic antagonism. Use Caution/Monitor. Patient should be closely observed for loss of blood glucose control; when drugs are withdrawn from a patient receiving metformin, patient should be observed closely for hypoglycemia
felodipine + rivaroxaban	1	felodipine increases levels of rivaroxaban by affecting hepatic/intestinal enzyme CYP3A4 metabolism. Use Caution/Monitor. Patients with renal impairment receiving rivaroxaban with drugs that are combined P-gp and weak or moderate CYP3A4 inhibitors may have significant increases in exposure compared with patients with normal renal function and no inhibitor use, since both pathways of rivaroxaban elimination are affected. Since these increases may increase bleeding risk, use rivaroxaban in this situation only if the potential benefit justifies the potential risk
ferrous sulfate + pantoprazole	1	pantoprazole will decrease the level or effect of ferrous sulfate by increasing gastric pH. Applies only to oral form of both agents. Use Caution/Monitor
formoterol + indapamide	1	formoterol and indapamide both decrease serum potassium. Use Caution/Monitor
formoterol + olodaterol inhaled	1	formoterol and olodaterol inhaled both increase sympathetic (adrenergic) effects, including increased blood pressure and heart rate. Use Caution/Monitor. Caution with coadministration of adrenergic drugs by any route because of additive sympathetic effects
furosemide + metoprolol	1	metoprolol increases and furosemide decreases serum potassium. Effect of interaction is not clear, use caution. Use Caution/Monitor

furosemide + valsartan	1	valsartan increases and furosemide decreases serum potassium. Effect of interaction is not clear, use caution. Use Caution/Monitor
garlic + nettle	1	garlic increases and nettle decreases anticoagulation. Effect of interaction is not clear, use caution. Use Caution/Monitor
heparin + losartan	1	heparin increases toxicity of losartan by Other (see comment). Use Caution/Monitor. Comment: Low molecular weight heparins may suppress adrenal aldosterone secretion, which can potentially cause hyperkalemia
hydrochlorothiazide + spironolactone	1	spironolactone increases and hydrochlorothiazide decreases serum potassium. Effect of interaction is not clear, use caution. Modify Therapy/Monitor Closely
hydrochlorothiazide + valsartan	1	valsartan increases and hydrochlorothiazide decreases serum potassium. Effect of interaction is not clear, use caution. Use Caution/Monitor
ibuprofen + indapamide	1	ibuprofen increases and indapamide decreases serum potassium. Effect of interaction is not clear, use caution. Use Caution/Monitor
ibuprofen + lisinopril	1	lisinopril, ibuprofen. Either increases toxicity of the other by Other (see comment). Use Caution/Monitor. Comment: May result in renal function deterioration, particularly in elderly or volume depleted individuals
indapamide + ketorolac	1	ketorolac increases and indapamide decreases serum potassium. Effect of interaction is not clear, use caution. Use Caution/Monitor
indapamide + lornoxicam	1	lornoxicam increases and indapamide decreases serum potassium. Effect of interaction is not clear, use caution. Use Caution/Monitor
indapamide + naproxen	1	naproxen increases and indapamide decreases serum potassium. Effect of interaction is not clear, use caution. Use Caution/Monitor
indapamide + nebivolol	1	nebivolol increases and indapamide decreases serum potassium. Effect of interaction is not clear, use caution. Use Caution/Monitor
indapamide + potassium	1	potassium chloride increases and indapamide decreases serum potassium. Effect of interaction is not clear, use caution. Modify Therapy/Monitor Closely
indapamide + telmisartan	1	telmisartan increases and indapamide decreases serum potassium. Effect of interaction is not clear, use caution. Use Caution/Monitor
indapamide + timolol	1	timolol increases and indapamide decreases serum potassium. Effect of interaction is not clear, use caution. Use Caution/Monitor
insulin + levofloxacin	1	levofloxacin increases effects of insulin aspart by pharmacodynamic synergism. Use Caution/Monitor. Quinolone antibiotic administration may result in hyper- or hypoglycemia. Gatifloxacin is most likely to produce dysglycemia; moxifloxacin is least likely
insulin + lixisenatide (DSC)	1	lixisenatide (DSC), insulin degludec. Either increases effects of the other by pharmacodynamic synergism. Modify Therapy/Monitor Closely. Risk of hypoglycemia increased when coadministered with basal insulins. Basal insulin dose reduction may be required
insulin + perindopril	1	perindopril, insulin degludec. Either increases effects of the other by pharmacodynamic synergism. Use Caution/Monitor. Both drugs decrease blood glucose
ivabradine + sotalol	1	ivabradine, sotalol. Either increases effects of the other by pharmacodynamic synergism. Modify Therapy/Monitor Closely. Most patients receiving ivabradine will also be treated with a beta-blocker. The risk of bradycardia increases with coadministration of drugs that slow heart

		rate (eg, digoxin, amiodarone, beta-blockers). Monitor heart rate in patients taking ivabradine with other negative chronotropes
ketoprofen + perindopril	1	perindopril, ketoprofen. Either increases toxicity of the other by Other (see comment). Use Caution/Monitor. Comment: May result in renal function deterioration, particularly in elderly or volume depleted individuals
ketoprofen + ramipril	1	ramipril, ketoprofen. Either increases toxicity of the other by Other (see comment). Use Caution/Monitor. Comment: May result in renal function deterioration, particularly in elderly or volume depleted individuals
ketoprofen + spironolactone	1	spironolactone and ketoprofen both increase serum potassium. Modify Therapy/Monitor Closely
ketoprofen + torsemide	1	ketoprofen increases and torsemide decreases serum potassium. Effect of interaction is not clear, use caution. Use Caution/Monitor
ketorolac + rivaroxaban	1	rivaroxaban, ketorolac. Other (see comment). Use Caution/Monitor. Comment: NSAIDs are known to increase bleeding. Bleeding risk may be increased when NSAIDs are used concomitantly with rivaroxaban. Monitor for signs/symptoms of blood loss
levofloxacin + magnesium sulfate	1	magnesium sulfate decreases levels of levofloxacin by inhibition of GI absorption. Applies only to oral form of both agents. Use Caution/Monitor. Separate by 2 hours
levofloxacin + metformin	1	levofloxacin increases effects of metformin by pharmacodynamic synergism. Use Caution/Monitor. Quinolone antibiotic administration may result in hyper- or hypoglycemia. Gatifloxacin is most likely to produce dysglycemia; moxifloxacin is least likely
lornoxicam + losartan	2	losartan and lornoxicam both increase serum potassium. Use Caution/Monitor
lornoxicam + spironolactone	1	spironolactone and lornoxicam both increase serum potassium. Modify Therapy/Monitor Closely
lornoxicam + torsemide	1	lornoxicam increases and torsemide decreases serum potassium. Effect of interaction is not clear, use caution. Use Caution/Monitor
losartan + tizanidine	1	tizanidine increases effects of losartan by pharmacodynamic synergism. Use Caution/Monitor. Risk of hypotension
magnesium hydroxide + vitamin D	1	vitamin D increases levels of magnesium hydroxide by Other (see comment). Use Caution/Monitor. Comment: Vitamin D can increase serum magnesium concentrations, particularly in the presence of renal impairment. The combined use of vitamin D and magnesium-containing products should be avoided, if possible, in patients with chronic renal failure
magnesium supplement + nifedipine	1	magnesium supplement, nifedipine. Either increases toxicity of the other by Other (see comment). Use Caution/Monitor. Comment: Calcium channel blockers may increase toxic effects of magnesium; magnesium may increase hypotensive effects of calcium channel blockers
magnesium hydroxide + torsemide	1	torsemide decreases levels of magnesium hydroxide by increasing renal clearance. Minor/Significance Unknown
meloxicam + torsemide	1	meloxicam increases and torsemide decreases serum potassium. Effect of interaction is not clear, use caution. Use Caution/Monitor

meloxicam + warfarin	1	meloxicam, warfarin. Either increases effects of the other by pharmacodynamic synergism. Modify Therapy/Monitor Closely. Drugs with antiplatelet properties may increase anticoagulation effect of warfarin
metformin + nifedipine	1	nifedipine decreases effects of metformin by pharmacodynamic antagonism. Use Caution/Monitor. Patient should be closely observed for loss of blood glucose control; when drugs are withdrawn from a patient receiving metformin, patient should be observed closely for hypoglycemia
metformin + ramipril	2	ramipril increases toxicity of metformin by unspecified interaction mechanism. Use Caution/Monitor. Increases risk for hypoglycemia and lactic acidosis
methotrexate + omeprazole	1	omeprazole increases levels of methotrexate by decreasing renal clearance. Use Caution/Monitor. Temporary withdrawal of PPI may be considered in some patients
nifedipine + sotalol	1	sotalol and nifedipine both increase anti-hypertensive channel blocking. Modify Therapy/Monitor Closely
nitroglycerin + verapamil	1	verapamil, nitroglycerin sublingual. Either increases toxicity of the other by additive vasodilation. Modify Therapy/Monitor Closely. Marked orthostatic hypotension reported with concomitant use
olmesartan + torsemide	1	olmesartan increases and torsemide decreases serum potassium. Effect of interaction is not clear, use caution. Use Caution/Monitor
omeprazole + tinidazole	1	omeprazole will increase the level or effect of tinidazole by affecting hepatic/intestinal enzyme CYP3A4 metabolism. Use Caution/Monitor
rivaroxaban + saw palmetto	1	saw palmetto increases toxicity of rivaroxaban by unspecified interaction mechanism. Use Caution/Monitor. May increase risk of bleeding
rivaroxaban + verapamil	1	verapamil increases levels of rivaroxaban by affecting hepatic/intestinal enzyme CYP3A4 metabolism. Use Caution/Monitor. Verapamil also inhibits P-gp activity, which can further increase rivaroxaban serum levels; since both pathways of rivaroxaban elimination are affected, patients with renal impairment receiving rivaroxaban with drugs that are combined P-gp and moderate CYP3A4 inhibitors may increase exposure compared to patients with normal renal function; monitor for bleeding
rosuvastatin + sodium bicarbonate	1	sodium bicarbonate decreases levels of rosuvastatin by inhibition of GI absorption. Applies only to oral form of both agents. Use Caution/Monitor. Separate by 2 hours
spironolactone + telmisartan	1	telmisartan and spironolactone both increase serum potassium. Modify Therapy/Monitor Closely

Note: Impact of drug–drug interactions associated with the combinations "aspirin + captopril" and "aspirin + enalapril" may be considered insignificant due to the use of low-dose aspirin in the majority of cases. Administration of aspirin at doses less than 300 mg per day has little effect on the effectiveness of captopril and enalapril. Administration of aspirin in higher doses reduces the effectiveness of captopril and enalapril.

Table S11. Drug combinations resulting in potential minor drug–drug interactions in the list of prescribed medications in patients with cardiovascular diseases according to data derived from the electronic health records (*n* = 1030) established in 2018–2023.

Drug combinations	*n*	Potential minor drug-drug interactions (P-list)
magnesium hydroxide + torsemide	65	torsemide decreases levels of magnesium hydroxide by increasing renal clearance. Minor/Significance Unknown
magnesium hydroxide + spironolactone	45	spironolactone increases levels of magnesium hydroxide by decreasing renal clearance. Minor/Significance Unknown
clopidogrel + torsemide	44	clopidogrel increases levels of torsemide by decreasing metabolism. Minor/Significance Unknown
aspirin + eplerenone	35	aspirin decreases effects of eplerenone by pharmacodynamic antagonism. Minor/Significance Unknown. NSAIDs decrease prostaglandin synthesis
aspirin + indapamide	32	indapamide will increase the level or effect of aspirin by acidic (anionic) drug competition for renal tubular clearance. Minor/Significance Unknown
ascorbic acid + aspirin	22	ascorbic acid will increase the level or effect of aspirin by acidic (anionic) drug competition for renal tubular clearance. Minor/Significance Unknown
		aspirin decreases levels of ascorbic acid by increasing renal clearance. Minor/Significance Unknown
		ascorbic acid increases levels of aspirin by decreasing renal clearance. Minor/Significance Unknown
indapamide + magnesium hydroxide	21	indapamide decreases levels of magnesium hydroxide by increasing renal clearance. Minor/Significance Unknown
aspirin + hydrochlorothiazide	13	hydrochlorothiazide will increase the level or effect of aspirin by acidic (anionic) drug competition for renal tubular clearance. Minor/Significance Unknown
budesonide + torsemide	11	budesonide, torsemide. Mechanism: pharmacodynamic synergism. Minor/Significance Unknown. Risk of hypokalemia, especially with strong glucocorticoid activity
aspirin + furosemide	10	aspirin decreases effects of furosemide by pharmacodynamic antagonism. Minor/Significance Unknown. NSAIDs decrease prostaglandin synthesis
formoterol + torsemide	10	formoterol, torsemide. Mechanism: pharmacodynamic synergism. Minor/Significance Unknown. Hypokalemia
hydrochlorothiazide + magnesium hydroxide	10	hydrochlorothiazide decreases levels of magnesium hydroxide by increasing renal clearance. Minor/Significance Unknown
cyanocobalamin + omeprazole	9	omeprazole decreases levels of cyanocobalamin by inhibition of GI absorption. Applies only to oral form of both agents. Minor/Significance Unknown
diclofenac topical + meloxicam	6	diclofenac topical, meloxicam. Either increases effects of the other by pharmacodynamic synergism. Minor/Significance Unknown. Although low, there is systemic exposure to diclofenac topical; theoretically, concomitant administration with systemic NSAIDS or aspirin may result in increased NSAID adverse effects
furosemide + magnesium hydroxide	5	furosemide decreases levels of magnesium hydroxide by increasing renal clearance. Minor/Significance Unknown
aspirin + chlorthalidone	4	chlorthalidone will increase the level or effect of aspirin by acidic (anionic) drug competition for renal tubular clearance. Minor/Significance Unknown

aspirin + cyanocobalamin	4	aspirin decreases levels of cyanocobalamin by inhibition of GI absorption. Applies only to oral form of both agents. Minor/Significance Unknown
carvedilol + omeprazole	4	omeprazole will increase the level or effect of carvedilol by affecting hepatic enzyme CYP2C9/10 metabolism. Minor/Significance Unknown
chlorthalidone + magnesium hydroxide	4	chlorthalidone decreases levels of magnesium hydroxide by increasing renal clearance. Minor/Significance Unknown
hydrochlorothiazide + metformin	4	hydrochlorothiazide will increase the level or effect of metformin by basic (cationic) drug competition for renal tubular clearance. Minor/Significance Unknown
		hydrochlorothiazide decreases effects of metformin by pharmacodynamic antagonism. Minor/Significance Unknown. Thiazide dosage >50 mg/day may increase blood glucose
indapamide + metformin	4	indapamide decreases effects of metformin by pharmacodynamic antagonism. Minor/Significance Unknown. Thiazide dosage >50 mg/day may increase blood glucose
bisoprolol + tizanidine	3	tizanidine increases effects of bisoprolol by pharmacodynamic synergism. Minor/Significance Unknown. Risk of hypotension
captopril + tizanidine	3	tizanidine increases effects of captopril by pharmacodynamic synergism. Minor/Significance Unknown. Risk of hypotension
carbamazepine + omeprazole	3	omeprazole increases levels of carbamazepine by decreasing metabolism. Minor/Significance Unknown. Monitor plasma levels when used concomitantly
cyanocobalamin + pantoprazole	3	pantoprazole decreases levels of cyanocobalamin by inhibition of GI absorption. Applies only to oral form of both agents. Minor/Significance Unknown
aluminum hydroxide + aspirin	2	aluminum hydroxide, aspirin. Mechanism: passive renal tubular reabsorption due to increased pH. Minor/Significance Unknown. Salicylate levels increased at moderate doses; salicylate levels decreased at large doses (d/t increased renal excretion of unchanged salicylic acid)
amiodarone + celecoxib	2	amiodarone will increase the level or effect of celecoxib by affecting hepatic enzyme CYP2C9/10 metabolism. Minor/Significance Unknown
amiodarone + pyridoxine	2	pyridoxine increases toxicity of amiodarone by unspecified interaction mechanism. Minor/Significance Unknown. Increased risk of photosensitivity
amlodipine + tizanidine	2	tizanidine increases effects of amlodipine by pharmacodynamic synergism. Minor/Significance Unknown. Risk of hypotension
aspirin + ascorbic acid	2	ascorbic acid will increase the level or effect of aspirin by acidic (anionic) drug competition for renal tubular clearance. Minor/Significance Unknown
		aspirin decreases levels of ascorbic acid by increasing renal clearance. Minor/Significance Unknown
		ascorbic acid increases levels of aspirin by decreasing renal clearance. Minor/Significance Unknown
aspirin + calcium carbonate	2	calcium carbonate, aspirin. Mechanism: passive renal tubular reabsorption due to increased pH. Minor/Significance Unknown. Salicylate levels increased at moderate doses; salicylate levels decreased at large doses (d/t increased renal excretion of unchanged salicylic acid)
aspirin + folic acid	2	aspirin decreases levels of folic acid by inhibition of GI absorption. Applies only to oral form of both agents. Minor/Significance Unknown
aspirin + ketoprofen	2	aspirin will increase the level or effect of ketoprofen by acidic (anionic) drug competition for renal tubular clearance. Minor/Significance Unknown

aspirin + verapamil	2	verapamil increases effects of aspirin by unknown mechanism. Minor/Significance Unknown. Enhanced antiplatelet activity
budesonide + eplerenone	2	budesonide will decrease the level or effect of eplerenone by affecting hepatic/intestinal enzyme CYP3A4 metabolism. Minor/Significance Unknown
budesonide + insulin	2	budesonide decreases effects of insulin regular human by pharmacodynamic antagonism. Minor/Significance Unknown
budesonide + metformin	2	budesonide decreases effects of metformin by pharmacodynamic antagonism. Minor/Significance Unknown
captopril + tamsulosin	2	tamsulosin, captopril. Either increases effects of the other by pharmacodynamic synergism. Minor/Significance Unknown. May increase risk of hypotension
carbamazepine + cyanocobalamin	2	carbamazepine decreases levels of cyanocobalamin by inhibition of GI absorption. Applies only to oral form of both agents. Minor/Significance Unknown
celecoxib + meloxicam	2	celecoxib will increase the level or effect of meloxicam by acidic (anionic) drug competition for renal tubular clearance. Minor/Significance Unknown
chlorthalidone + metformin	2	chlorthalidone decreases effects of metformin by pharmacodynamic antagonism. Minor/Significance Unknown. Thiazide dosage >50 mg/day may increase blood glucose
cyanocobalamin + gabapentin	2	gabapentin decreases levels of cyanocobalamin by inhibition of GI absorption. Applies only to oral form of both agents. Minor/Significance Unknown
digoxin + magnesium hydroxide	2	digoxin decreases levels of magnesium hydroxide by increasing renal clearance. Minor/Significance Unknown
folic acid + indapamide	2	indapamide decreases levels of folic acid by increasing renal clearance. Minor/Significance Unknown
folic acid + methotrexate	2	folic acid decreases effects of methotrexate by pharmacodynamic antagonism. Minor/Significance Unknown. Vitamin preparations containing folic acid or its derivatives may decrease responses to systemically administered methotrexate
formoterol + furosemide	2	formoterol, furosemide. Mechanism: pharmacodynamic synergism. Minor/Significance Unknown. Hypokalemia
ketoprofen + meloxicam	2	ketoprofen will increase the level or effect of meloxicam by acidic (anionic) drug competition for renal tubular clearance. Minor/Significance Unknown
levothyroxine + omeprazole	2	omeprazole decreases levels of levothyroxine by increasing gastric pH. Applies only to oral form of both agents. Minor/Significance Unknown. Conflicting evidence regarding this interaction exists
aceclofenac + aspirin	1	aceclofenac will increase the level or effect of aspirin by acidic (anionic) drug competition for renal tubular clearance. Minor/Significance Unknown
aceclofenac + hydrochlorothiazide	1	hydrochlorothiazide will increase the level or effect of aceclofenac by acidic (anionic) drug competition for renal tubular clearance. Minor/Significance Unknown
aceclofenac + indapamide	1	indapamide will increase the level or effect of aceclofenac by acidic (anionic) drug competition for renal tubular clearance. Minor/Significance Unknown
aceclofenac + lisinopril	1	aceclofenac decreases effects of lisinopril by pharmacodynamic antagonism. Minor/Significance Unknown. NSAIDs decrease prostaglandin synthesis
acetazolamide + aspirin	1	aspirin will decrease the level or effect of acetazolamide by affecting hepatic/intestinal enzyme CYP3A4 metabolism. Minor/Significance Unknown

acetazolamide + atorvastatin	1	acetazolamide will increase the level or effect of atorvastatin by affecting hepatic/intestinal enzyme CYP3A4 metabolism. Minor/Significance Unknown
acetylcysteine + nitroglycerin	1	acetylcysteine increases effects of nitroglycerin sublingual by Other (see comment). Minor/Significance Unknown. Comment: Acetylcysteine may enhance vasodilatory effects of nitroglycerin
amitriptyline + carbamazepine	1	carbamazepine decreases levels of amitriptyline by increasing metabolism. Minor/Significance Unknown
amoxicillin + clarithromycin	1	clarithromycin decreases effects of amoxicillin by pharmacodynamic antagonism. Minor/Significance Unknown
apixaban + dexamethasone	1	dexamethasone will decrease the level or effect of apixaban by affecting hepatic/intestinal enzyme CYP3A4 metabolism. Contraindicated. Reduces anticoagulant effect by decreasing apixaban systemic exposure
aspirin + cefixime	1	cefixime will increase the level or effect of aspirin by acidic (anionic) drug competition for renal tubular clearance. Minor/Significance Unknown
aspirin + celecoxib	1	aspirin will increase the level or effect of celecoxib by acidic (anionic) drug competition for renal tubular clearance. Minor/Significance Unknown
aspirin + diclofenac topical	1	diclofenac topical, aspirin. Either increases effects of the other by pharmacodynamic synergism. Minor/Significance Unknown. Although low, there is systemic exposure to diclofenac topical; theoretically, concomitant administration with systemic NSAIDS or aspirin may result in increased NSAID adverse effects
aspirin + ibuprofen	1	aspirin will increase the level or effect of ibuprofen by acidic (anionic) drug competition for renal tubular clearance. Minor/Significance Unknown
aspirin + ketorolac	1	aspirin will increase the level or effect of ketorolac by acidic (anionic) drug competition for renal tubular clearance. Minor/Significance Unknown
aspirin + lornoxicam	1	aspirin will increase the level or effect of lornoxicam by acidic (anionic) drug competition for renal tubular clearance. Minor/Significance Unknown
aspirin + meloxicam	1	aspirin will increase the level or effect of meloxicam by acidic (anionic) drug competition for renal tubular clearance. Minor/Significance Unknown
atorvastatin + orlistat	1	orlistat increases effects of atorvastatin by pharmacodynamic synergism. Minor/Significance Unknown
bisoprolol + escitalopram	1	escitalopram increases levels of bisoprolol by decreasing metabolism. Minor/Significance Unknown
budesonide + clarithromycin	1	budesonide will decrease the level or effect of clarithromycin by affecting hepatic/intestinal enzyme CYP3A4 metabolism. Minor/Significance Unknown
budesonide + furosemide	1	budesonide, furosemide. Mechanism: pharmacodynamic synergism. Minor/Significance Unknown. Risk of hypokalemia, especially with strong glucocorticoid activity
calcium carbonate + manganese	1	calcium carbonate, manganese. Either decreases levels of the other by inhibition of GI absorption. Applies only to oral form of both agents. Minor/Significance Unknown. Separate by 2 hours
captopril + lornoxicam	1	lornoxicam decreases effects of captopril by pharmacodynamic antagonism. Minor/Significance Unknown. NSAIDs decrease prostaglandin synthesis

carbamazepine + flurbiprofen	1	carbamazepine will decrease the level or effect of flurbiprofen by affecting hepatic enzyme CYP2C9/10 metabolism. Minor/Significance Unknown.
carbamazepine + folic acid	1	carbamazepine decreases levels of folic acid by unspecified interaction mechanism. Minor/Significance Unknown
carbamazepine + meloxicam	1	carbamazepine will decrease the level or effect of meloxicam by affecting hepatic enzyme CYP2C9/10 metabolism. Minor/Significance Unknown
carbamazepine + oxcarbazepine	1	carbamazepine decreases levels of oxcarbazepine by increasing metabolism. Minor/Significance Unknown
celecoxib + indapamide	1	indapamide will increase the level or effect of celecoxib by acidic (anionic) drug competition for renal tubular clearance. Minor/Significance Unknown
chlorthalidone + meloxicam	1	chlorthalidone will increase the level or effect of meloxicam by acidic (anionic) drug competition for renal tubular clearance. Minor/Significance Unknown
chlorthalidone + tizanidine	1	tizanidine increases effects of chlorthalidone by pharmacodynamic synergism. Minor/Significance Unknown. Risk of hypotension
cyanocobalamin + esomeprazole	1	esomeprazole
cyanocobalamin + metformin	1	metformin decreases levels of cyanocobalamin by unspecified interaction mechanism. Minor/Significance Unknown. It may take several years of metformin therapy to develop vitamin B12 deficiency
cyanocobalamin + rabeprazole	1	rabeprazole decreases levels of cyanocobalamin by inhibition of GI absorption. Applies only to oral form of both agents. Minor/Significance Unknown
dexamethasone + ciprofloxacin	1	dexamethasone and ciprofloxacin both increase Other (see comment). Use Caution/Monitor. Coadministration of quinolone antibiotics and corticosteroids may increase risk of tendon rupture
dexamethasone + pantoprazole	1	dexamethasone will decrease the level or effect of pantoprazole by affecting hepatic/intestinal enzyme CYP3A4 metabolism. Minor/Significance Unknown
diclofenac + lornoxicam	1	diclofenac will increase the level or effect of lornoxicam by acidic (anionic) drug competition for renal tubular clearance. Minor/Significance Unknown
diclofenac topical + ketoprofen	1	diclofenac topical, ketoprofen. Either increases effects of the other by pharmacodynamic synergism. Minor/Significance Unknown. Although low, there is systemic exposure to diclofenac topical; theoretically, concomitant administration with systemic NSAIDS or aspirin may result in increased NSAID adverse effects
felodipine + loratadine	1	felodipine will increase the level or effect of loratadine by P-glycoprotein (MDR1) efflux transporter. Minor/Significance Unknown
folic acid + furosemide	1	furosemide decreases levels of folic acid by increasing renal clearance. Minor/Significance Unknown
folic acid + metformin	1	metformin decreases levels of folic acid by unspecified interaction mechanism. Minor/Significance Unknown
folic acid + torsemide	1	torsemide decreases levels of folic acid by increasing renal clearance. Minor/Significance Unknown
formoterol + indapamide	1	formoterol, indapamide. Mechanism: pharmacodynamic synergism. Minor/Significance Unknown. Hypokalemia

furosemide + magnesium sulfate	1	furosemide decreases levels of magnesium sulfate by increasing renal clearance. Minor/Significance Unknown
furosemide + spironolactone	1	spironolactone increases and furosemide decreases serum potassium. Effect of interaction is not clear, use caution. Modify Therapy/Monitor Closely
ibuprofen + indapamide	1	indapamide will increase the level or effect of ibuprofen by acidic (anionic) drug competition for renal tubular clearance. Minor/Significance Unknown
indapamide + insulin glargine	1	indapamide decreases effects of insulin glargine by pharmacodynamic antagonism. Minor/Significance Unknown. Thiazide dosage >50 mg/day may increase blood glucose
indapamide + lornoxicam	1	indapamide will increase the level or effect of lornoxicam by acidic (anionic) drug competition for renal tubular clearance. Minor/Significance Unknown
indapamide + naproxen	1	indapamide will increase the level or effect of naproxen by acidic (anionic) drug competition for renal tubular clearance. Minor/Significance Unknown
indapamide + tizanidine	1	tizanidine increases effects of indapamide by pharmacodynamic synergism. Minor/Significance Unknown. Risk of hypotension
lisinopril + lornoxicam	1	lornoxicam decreases effects of lisinopril by pharmacodynamic antagonism. Minor/Significance Unknown. NSAIDs decrease prostaglandin synthesis
lisinopril + tizanidine	1	tizanidine increases effects of lisinopril by pharmacodynamic synergism. Minor/Significance Unknown. Risk of hypotension
metformin + perindopril	1	perindopril increases toxicity of metformin by unspecified interaction mechanism. Use Caution/Monitor. Increases risk for hypoglycemia and lactic acidosis
spironolactone + tizanidine	1	tizanidine increases effects of spironolactone by pharmacodynamic synergism. Minor/Significance Unknown. Risk of hypotension
thiamine + torsemide	1	torsemide decreases levels of thiamine by increasing renal clearance. Minor/Significance Unknown
tizanidine + torsemide	1	tizanidine increases effects of torsemide by pharmacodynamic synergism. Minor/Significance Unknown. Risk of hypotension
